# PRMT1-dependent regulation of RNA metabolism and DNA damage response sustains pancreatic ductal adenocarcinoma

**DOI:** 10.1038/s41467-021-24798-y

**Published:** 2021-07-30

**Authors:** Virginia Giuliani, Meredith A. Miller, Chiu-Yi Liu, Stella R. Hartono, Caleb A. Class, Christopher A. Bristow, Erika Suzuki, Lionel A. Sanz, Guang Gao, Jason P. Gay, Ningping Feng, Johnathon L. Rose, Hideo Tomihara, Joseph R. Daniele, Michael D. Peoples, Jennifer P. Bardenhagen, Mary K. Geck Do, Qing E. Chang, Bhavatarini Vangamudi, Christopher Vellano, Haoqiang Ying, Angela K. Deem, Kim-Anh Do, Giannicola Genovese, Joseph R. Marszalek, Jeffrey J. Kovacs, Michael Kim, Jason B. Fleming, Ernesto Guccione, Andrea Viale, Anirban Maitra, M. Emilia Di Francesco, Timothy A. Yap, Philip Jones, Giulio Draetta, Alessandro Carugo, Frederic Chedin, Timothy P. Heffernan

**Affiliations:** 1 Traction, The University of Texas MD Anderson Cancer Center, Houston, TX USA; 2 Department of Molecular and Cellular Biology and Genome Center, University of California, Davis, CA USA; 3 Department of Biostatistics, The University of Texas MD Anderson Cancer Center, Houston, TX USA; 4 Department of Genomic Medicine, The University of Texas MD Anderson Cancer Center, Houston, TX USA; 5 Institute for Applied Cancer Science, The University of Texas MD Anderson Cancer Center, Houston, TX USA; 6 ORBIT, The University of Texas MD Anderson Cancer Center, Houston, TX USA; 7 Department of Cellular and Molecular Oncology, The University of Texas MD Anderson Cancer Center, Houston, TX USA; 8 Department of Genitourinary Medical Oncology, The University of Texas MD Anderson Cancer Center, Houston, TX USA; 9 Department of Surgical Oncology, The University of Texas MD Anderson Cancer Center, Houston, TX USA; 10 Department of Oncological Sciences and Pharmacological Sciences at Icahn School of Medicine at Mount Sinai, New York, NY USA; 11 Sheikh Ahmed Bin Zayed Al Nahyan Center for Pancreatic Cancer Research, The University of Texas MD Anderson Cancer Center, Houston, TX USA; 12 Department of Investigational Cancer Therapeutics (Phase I Program), The University of Texas MD Anderson Cancer Center, Houston, Texas USA; 13 Present Address: Department of Pharmaceutical Sciences, Butler University, Indianapolis, IN USA; 14 Present Address: Department of Surgery, Kindai University Nara Hospital, Nara, JP USA; 15 Present Address: Exo Therapeutics, Cambridge, MA USA; 16 Present Address: Division of Gastrointestinal Oncology, H. Lee Moffitt Cancer Center, Tampa, FL USA

**Keywords:** Pancreatic cancer, Mechanisms of disease

## Abstract

Pancreatic ductal adenocarcinoma (PDAC) is an aggressive cancer that has remained clinically challenging to manage. Here we employ an RNAi-based in vivo functional genomics platform to determine epigenetic vulnerabilities across a panel of patient-derived PDAC models. Through this, we identify protein arginine methyltransferase 1 (PRMT1) as a critical dependency required for PDAC maintenance. Genetic and pharmacological studies validate the role of PRMT1 in maintaining PDAC growth. Mechanistically, using proteomic and transcriptomic analyses, we demonstrate that global inhibition of asymmetric arginine methylation impairs RNA metabolism, which includes RNA splicing, alternative polyadenylation, and transcription termination. This triggers a robust downregulation of multiple pathways involved in the DNA damage response, thereby promoting genomic instability and inhibiting tumor growth. Taken together, our data support PRMT1 as a compelling target in PDAC and informs a mechanism-based translational strategy for future therapeutic development.

**Statement of significance**

PDAC is a highly lethal cancer with limited therapeutic options. This study identified and characterized PRMT1-dependent regulation of RNA metabolism and coordination of key cellular processes required for PDAC tumor growth, defining a mechanism-based translational hypothesis for PRMT1 inhibitors.

## Abstract (web summary)

Arginine methylation by PRMTs is dysregulated in cancer. Here, the authors use functional genomics screens and identify PRMT1 as a vulnerability in pancreatic ductal adenocarcinoma, and further show that PRMT1 regulates RNA metabolism and coordinates expression of genes in cell cycle progression, maintaining genomic stability and tumour growth.

## Introduction

Pancreatic cancer is projected to become the second leading cause of tumor-related deaths by 2030^1^ due to its increasing incidence, poor overall 5-year survival rate, and limited therapeutic options. Pancreatic ductal adenocarcinoma (PDAC) accounts for more than 80% of pancreatic cancer cases^2^. Next-generation sequencing (NGS) studies have uncovered the molecular mechanisms that contribute to PDAC pathogenesis, including oncogenic mutations in *KRAS* and inactivating mutations in the tumor suppressor genes *TP53*, *SMAD4*, and *CDKN2A*^2^. Unbiased sequencing studies have also identified lower-frequency mutational events impacting core biological networks, which include DNA damage repair (DDR), axon guidance, and epigenetic regulation^3,4^.

Given the penetrance of loss of-function epigenetic mutations, such as alterations in histone-modifying enzymes and chromatin remodeling complexes^3,4^, we aimed to identify specific epigenetic vulnerabilities that can be exploited therapeutically. We thus employed our in vivo target discovery platform, PILOT (Patient-based In vivo Lethality to Optimize Treatment),^5^ to systematically uncover epigenetic dependencies in a panel of patient-derived xenograft (PDX) models of PDAC. Through this unbiased approach, we identified protein arginine methyltransferase 1 (*PRMT1*) as a top-scoring hit and a novel genetic vulnerability in PDAC.

Arginine methylation is a common post-translational modification that regulates multiple cellular processes^6‒8^. Protein arginine methyltransferases (PRMTs), the only enzymes that mediate this reaction, catalyze the transfer of a methyl group from S-adenosyl methionine (SAM) to the arginine residues of histone and nonhistone proteins^9^. All nine PRMT family members mediate the addition of one methyl group to one of the guanidine nitrogens of arginine, generating mono-methylarginine (MMA). PRMT family members are classified into three types based on the final methylarginine product that is generated. Specifically, Type I enzymes catalyze the addition of a second methyl group to the same nitrogen, producing asymmetric di-methylarginine (ADMA); Type II enzymes methylate additional guanidine nitrogen, producing symmetric dimethylarginine (SDMA); and the Type III enzyme, PRMT7, solely catalyzes MMA^6,9^. Interestingly, dysregulation of arginine methylation has been increasingly associated with cancer^10,11^, and PRMTs have thus garnered significant interest as therapeutic targets. Accordingly, several agents targeting PRMTs have been developed, with PRMT Type I and PRMT5 selective inhibitors currently under clinical investigation^12,13^.

In this study, we identify PRMT1 as a novel vulnerability in PDAC PDX models and demonstrate context-specific dependency using both genetic and pharmacological approaches. PRMT1 is the predominant Type I enzyme responsible for more than 85% of ADMA^14^, which regulates a variety of cellular processes^6,7^. However, the mechanistic basis of PRMT1 dependency within specific tissue and genetic contexts, including PDAC, remains poorly understood. Thus, we leveraged orthogonal proteomic and transcriptomic approaches to elucidate the molecular mechanism underlying the role of ADMA in PDAC. Our studies demonstrated that ADMA is required for faithful RNA processing as well as for the expression of multiple genes and pathways required for supporting PDAC maintenance and genome stability, including cell cycle and DDR networks. We also confirmed the requirement of PRMT1 to maintain in vivo growth of PDAC PDXs, suggesting a mechanism-based translational hypothesis for the clinical development of PRMT1 inhibitors.

## Results

### In vivo loss-of-function screens identify PRMT1 as a genetic vulnerability in PDAC

**Fig. 1:**
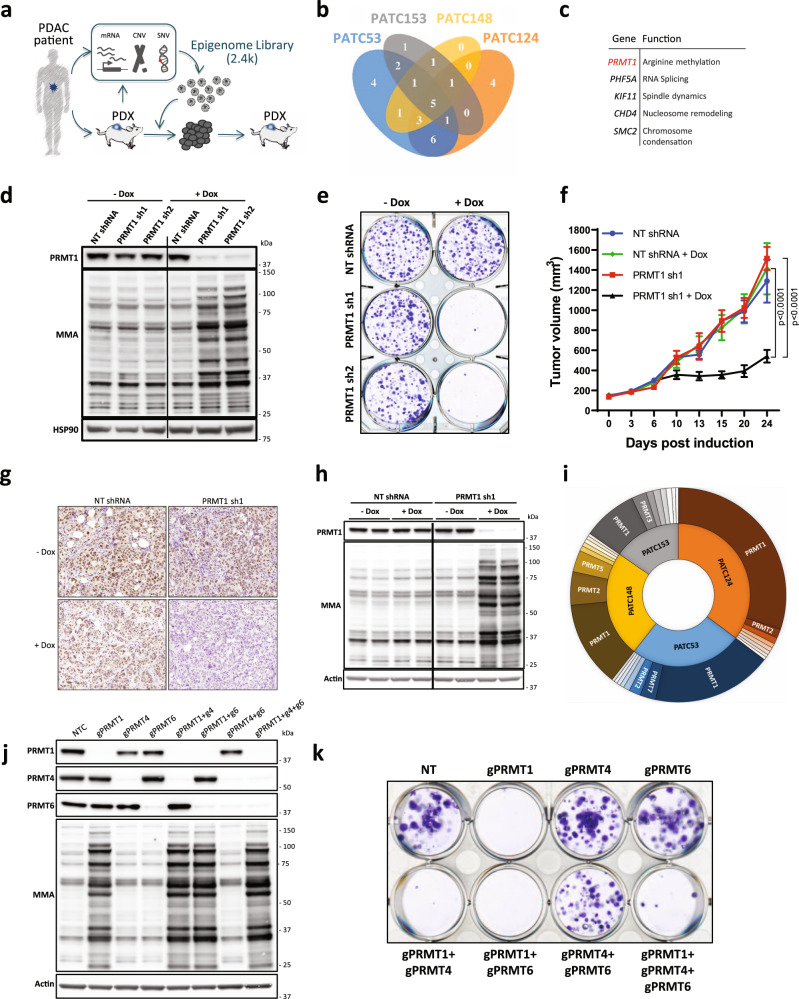
PRMT1 is a critical dependency in PDAC. **a** Schematic representation of the PILOT platform to inform on patient-centric genetic dependencies. **b** Venn diagram (4-ellipses) displaying individual and common top-scoring hits across in patient-derived xenograft (PDX) screens in vivo (RSA LogP ≤ −1.5 in at least one PDX and FDR ≤ 0.3). **c** Gene name and function of the common top 5 scoring hits emerging from the PILOT platform. **d** Western Blot analysis of PRMT1 expression and mono-methylarginine (MMA) changes in PATC53 cells. Cells were engineered with two independent doxycycline (DOX)-inducible *PRMT1*-targeting (sh1, sh2) or non-targeting (NT) shRNA, and treated with or without 0.5 µg/mL DOX for 72 h. **e** Colony formation assay. Representative crystal violet staining image of PATC53 cells engineered with two independent DOX-inducible *PRMT1*-targeting (sh1, sh2) or non-targeting (NT) shRNA and treated with or without 0.5 µg/mL DOX for 14 days. **f** Tumor growth curve (mm^3^) of PATC53 xenografts harboring DOX-inducible *PRMT1*-targeting (sh1) or non-targeting (NT) control (*n* = 6 mice/group). Mice were randomized to either a DOX diet (200 mg/Kg) or control chow upon tumor establishment (150 mm^3^). Data are presented as the mean ± SEM and *p* values are calculated by 2-way ANOVA with multiple comparisons and Tukey’s correction. **g**–**h** Evaluation of PRMT1 expression by immunohistochemistry (scale bar 50 µm, top left corner) (**g**) and Western Blot analysis (**h**) in PATC53 tumor lysates 10 days post-DOX-induction. MMA levels are also shown. **i** Sunburst plot representing the ranked impact, expressed as a percentage of 1/RSA p-value, of the different members of the PRMT family across genetic screens. **j**–**k** Effect of CRISPR/Cas9-mediated knock-down of PRMT1, PRMT4, and PRMT6 individually or in combination on global arginine methylation status (**j**) and on cell growth, as assessed by colony formation assay, in PATC53 cells. Representative crystal violet staining image (**k**). Source data are provided as a Source Data file.

We employed PILOT, an RNAi-based in vivo functional genomics platform, to identify epigenetic vulnerabilities across a panel of fully annotated patient-derived PDAC models with known engraftment efficiency (Supplementary Fig. 1a). These models were confirmed to harbor *KRAS* and *TP53* mutations in addition to lower frequency mutations in known PDAC-associated genes (Supplementary Data 1). Using a previously described approach^5^, we interrogated these models with a high-complexity lentiviral library targeting 237 epigenetic regulators (10 shRNA/gene) (Fig. 1a). Analysis of shRNA densities and fold change in tumors confirmed depletion of shRNAs that targeted genes essential for in vivo tumor growth compared to the cell population pre-transplantation (Supplementary Fig. 1b). Adequate separation of positive (*RPL30*, *PSMA1*) and negative (*LUC*) controls was confirmed (Supplementary Fig. 1c). By applying stringent thresholds (redundant shRNA activity (RSA) LogP ≤ −1.5 in at least one PDX and FDR ≤ 0.3), we captured individual (Supplementary Data 2) and common epigenetic vulnerabilities across PDX models (Fig. 1b and Supplementary Data 3). This analysis confirmed known synthetic lethal interactions, including *ARID1B* depletion in *ARID1A*-mutated PDAC tumors, which have been previously demonstrated in other tumor types^15^ (Supplementary Fig. 1d). These findings thus validate our ability to properly associate gene essentiality with relevant molecular features.

Through this effort, five genes emerged as common lethality factors in all PDAC PDX models (Fig. 1c). Two of these, *PHF5A* and *SMC2*, were consistent with our previous work in PDAC^5^. *KIF11* encodes a motor protein required for proper spindle assembly and has been reported as a top-scoring hit in genetic screens of cell fitness^16^. *CHD4* encodes a chromodomain-containing protein that catalyzes ATP-dependent nucleosome remodeling and was previously identified as a vulnerability in breast cancer^17^. We were particularly interested in *PRMT1*, which encodes for an arginine N-methyltransferase that was a top-scoring gene across all PDAC models (Supplementary Fig. 1e). PRMT1 belongs to a druggable family of enzymes, and PRMT type I inhibitors are currently under clinical investigation^12,13^. PRMT1 has been documented to drive pro-tumorigenic events in multiple tumor types^7,10^, including PDAC^18‒20^. However, there remains great interest in defining the mechanisms by which PRMT1 contributes to PDAC pathogenesis.

### PRMT1 is a critical dependency in PDAC

PRMT1 is the primary methyltransferase that catalyzes asymmetric dimethylation of arginine residues (ADMA). Accordingly, global depletion of ADMA with a concurrent increase of global arginine monomethylation (MMA) is indicative of efficient PRMT1 inhibition^14^. To validate the dependency of PDAC on PRMT1 expression, we engineered PATC53 cells, a patient-derived model used in the PILOT screening, with two independent, doxycycline-inducible shRNAs targeting *PRMT1* or a luciferase non-targeting (NT) control shRNA (Fig. 1d). Using methylarginine-specific antibodies (Supplementary Fig. 2a), we observed decreased levels of ADMA and accumulation of MMA in PRMT1-depleted cells compared to NT shRNA and no-doxycycline negative controls. The banding pattern on Western blots highlighted the large number of protein targets post-transcriptionally modified by PRMT1 (Fig. 1d, MMA; Supplementary Fig. 2b, ADMA). PRMT1 depletion dramatically inhibited in vitro cell growth of PATC53 (Fig. 1e and Supplementary Fig. 2c) as well as of other PDAC cell lines (Supplementary Fig. 3, 4) in colony formation assays, indicative of a strong dependency of PDAC cells on PRMT1. Ectopic overexpression of an shRNA-resistant *PRMT1* cDNA rescued the growth defect and restored ADMA and MMA back to physiological levels (Supplementary Fig. 5). These data suggest that the observed growth phenotype was due to inhibition of PRMT1.

To investigate the requirement for PRMT1 to support PDAC tumor growth in vivo, PATC53 cells harboring inducible *PRMT1* or NT shRNA were transplanted into immune-compromised mice and, upon tumor establishment, animals were randomized to receive doxycycline or control diet. Significant tumor growth inhibition (TGI) was observed over 24 days in animals with tumors derived from cells harboring *PRMT1*-targeting shRNA and treated with doxycycline compared to control groups (*PRMT1* sh1 + Dox versus NT shRNA + Dox: percentage of tumor growth inhibition (% TGI) = 68, ***p < 0.001; *PRMT1* sh1—Dox versus *PRMT1* sh1 + Dox: %TGI = 70, ***p < 0.001) (Fig. 1f and Supplementary Fig. 6a). Depletion of PRMT1 in tumors was confirmed by immunohistochemistry and Western blot analysis of tumor lysates (Figs. 1g and 1h, respectively). Consistent with in vitro results, robust depletion of global ADMA and accumulation of MMA was observed in tumor specimens (Fig. 1h and Supplementary Fig. 6b, c and d). Together, these results confirm a critical dependency of established human PDAC xenografts on PRMT1 expression in vivo.

Although PRMT1 is the primary Type I arginine methyltransferase responsible for a majority of ADMA, two other major Type I methyltranferases, PRMT4 (CARM1) and PRMT6, have also been linked to cancer^10,21^, thus prompting us to investigate a potential dependency of PDAC cells on these enzymes. Despite the functional overlap, neither *PRMT4* nor *PRMT6* emerged as a significantly depleted hit in any of the four models tested (Fig. 1i and Supplementary Data 2). To validate these screening results, we used a CRISPR/Cas9-based approach to knock out all three major Type I PRMTs, either individually or in combination, in PATC53 cells. Multiple guide RNAs (gRNA) induced efficient PRMT1, PRMT4, or PRMT6 depletion without affecting the expression levels of other PRMT family members (Supplementary Fig. 7a, 8a, and 9a). Accumulation of MMA was only observed in the context of PRMT1 deletion, confirming MMA accumulation as a selective biomarker for PRMT1 target engagement (Fig. 1j and Supplementary Fig. 7a, 8a, and 9a). A strong reduction of ADMA was observed in PRMT1-deleted cells, while ADMA was only modestly reduced in PRMT4- or PRMT6-deleted cells (Supplementary Fig. 7a, 8a, and 9a). These findings are consistent with reports from other models that PRMT1 acts as the primary Type I PRMT, catalyzing more than 85% of global ADMA deposition, specifically within the glycine-arginine rich (GAR) domains^9,14^. While a striking growth defect in colony formation assays was induced by PRMT1 deletion (Supplementary Fig. 7b and 7c), only a modest or no significant growth inhibition was observed in PRMT4- or PRMT6-deleted cells, respectively (Supplementary Fig. 8b and 8c; Supplementary Fig. 9b and 9c). The combined deletion of PRMT1, PRMT4, and PRMT6 further confirmed PRMT1 as the driver of the observed phenotypic response (Fig. 1k and Supplementary Fig. 9d). Taken together, these data establish that the evaluated patient-derived PDAC models rely on PRMT1 and are vulnerable to PRMT1 inhibition.

### Pharmacological inhibition of PRMT1 catalytic activity phenocopies genetic depletion and impairs PDAC growth

To complement our genetic studies, we leveraged a recently described potent Type I PRMT inhibitor^22^, GSK3368715 (referred to herein as PRMTi). Extensive characterization was completed to confirm the biochemical potency, selectivity, and cellular activity of PRMTi before deploying this compound in validation studies (Supplementary Fig. 10).

**Fig. 2:**
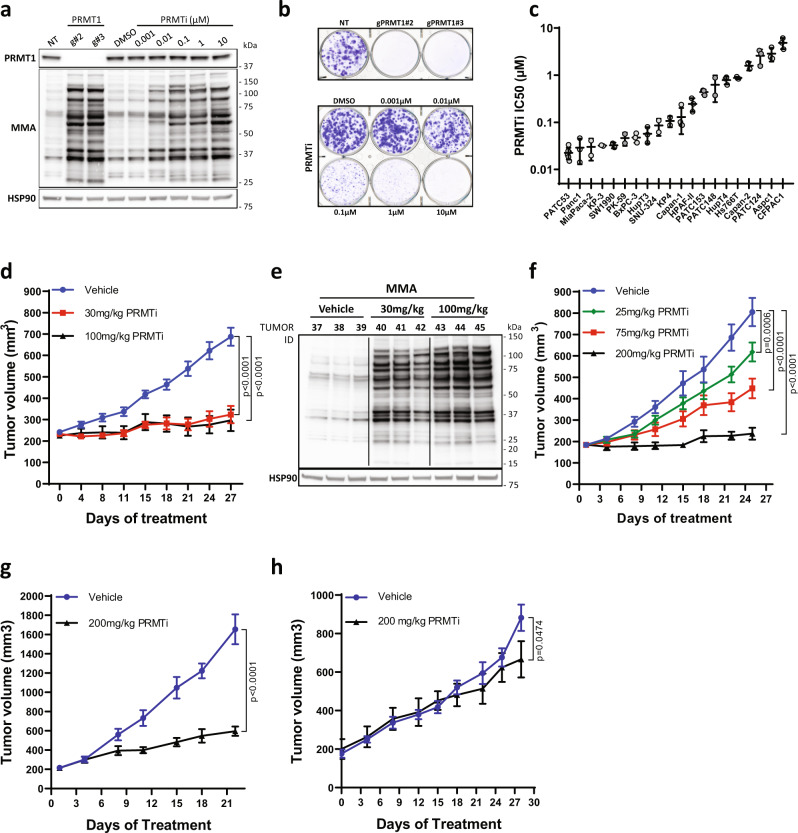
Inhibition of PRMT1 catalytic activity phenocopies genetic depletion and impairs PDAC growth. **a** Western Blot analysis of PRMT1 expression and MMA in PATC53 cells either engineered with two independent guide RNA targeting *PRMT1* or with a non-targeting (NT) control, or treated with DMSO or PRMTi in a dose-dependent manner for 48 h. **b** Colony formation assay. Representative crystal violet staining images of PATC53 cells upon CRISPR-Cas9-mediated knock-down of PRMT1 (upper panel) or treated with PRMTi in a dose-dependent manner (bottom panel). **c** PRMTi IC50 values across a panel of PDAC models as calculated by a dose-response curve in colony formation assay. Individual points represent IC50 values from independent experiments. Data are presented as the mean ± S.D. calculated from at least 2 independent experiments for each cell line. **d** Tumor growth curve (mm^3^) of PATC53 xenografts treated with PRMTi at 30 mg/kg and 100 mg/kg twice daily (BID). Data are presented as the mean ± SEM and *p* values are calculated by 2-way ANOVA with multiple comparisons and Tukey’s correction compared to vehicle control, *n* = 8–10 mice/group. **e** Western Blot analysis of MMA in PATC53 tumor lysates 7 days post-PRMTi treatment. **f** Tumor growth curve (mm^3^) of PANC1 xenografts treated with PRMTi at 25 mg/kg, 75 mg/kg, and 200 mg/kg once daily (QD). Data are presented as the mean ± SEM and *p* values are calculated by 2-way ANOVA with multiple comparisons and Tukey’s correction, compared to vehicle control (*n* = 8 mice/group). **g** Tumor growth curve (mm^3^) of the PATX153 PDX model treated with PRMTi at 200 mg/kg QD, 5on/2off. Data are presented as the mean ± SEM and *p* values are calculated by 2-way ANOVA with multiple comparisons and Sidak correction, compared to vehicle control (*n* = 5 mice/group). **h** Tumor growth curve (mm^3^) of the PATX60 PDX model treated with PRMTi at 200 mg/kg QD, 5on/2off. Data are presented as the mean ± SEM and *p* values are calculated by 2-way ANOVA with multiple comparisons and Sidak correction, compared to vehicle control (*n* = 4 mice/group vehicle; *n* = 3 mice/group PRMTi). Source data are provided as a Source Data file.

We first compared the effect of PRMT1 genetic and pharmacological inhibition, and observed that PRMTi-induced accumulation of MMA and depletion of ADMA were comparable to those observed upon genetic PRMT1 downregulation in PATC53 cells (Fig. 2a and Supplementary Fig. 11a) and other PDAC models (Supplementary Fig. 12a and f). PRMTi also led to robust suppression of in vitro cell growth comparable to genetic protein depletion in multiple PDAC models (Fig. 2b, Supplementary Fig. 11b and c, Supplementary Fig. 12 b–e and g–j), suggesting that PRMT1 catalytic activity is required for tumor cell growth. Of note, PRMTi induced only a modest response in normal diploid cells (Supplementary Fig. 11d–i) upon equivalent modulation of PRMT1 activity (Supplementary Fig. 11j). We next established dose-response curves for PRMTi across an extensive panel of generic and patient-derived PDAC models (Fig. 2c). This identified a range of sensitivity to PRMTi, in which PATC53 (Supplementary Fig. 11k) and PANC1 cells scored as the most sensitive.

To further verify PRMT1 as a critical dependency and, thus, a novel therapeutic vulnerability in PDAC, we investigated the response to PRMTi in vivo. PATC53 cells were implanted subcutaneously and, upon tumor establishment, mice were randomized to receive either vehicle or various doses of PRMTi given orally (PO) twice daily (BID). After four weeks of treatment, TGI was observed at tolerated doses (%TGI at 30 mg/kg = 80, *p* < 0.0001; %TGI at 100 mg/kg = 84, *p* < 0.0001; Fig. 2d and Supplementary Fig. 13a) in the PRMTi group compared to control group. Global arginine methylation was also evaluated in tumor lysates from a cohort of animals seven days post-randomization to drug administration, which confirmed a robust increase in MMA in PRMTi- versus control-treated animals (Fig. 2e) and correlated in vivo efficacy with PRMT1 target modulation. Dose-dependent responses to PRMTi in PANC1 xenografts were consistent with in vitro sensitivity data in PANC1 cells (Fig. 2f and Supplementary Fig. 13b and c).

We next evaluated response to PRMT1 inhibition in PDX models with maintained heterogeneity, host stroma, and tumor architecture ^23^. In PATX153, a model that was included in the initial in vivo loss of function screen, PRMTi resulted in a marked inhibition of tumor growth (Fig. 2g) at tolerated doses (Supplementary Fig. 13d). However, PRMTi treatment of a different PDAC PDX model, PATX60, induced only a minimal response (Fig. 2h and Supplementary Fig. 13e), indicating that response to ADMA depletion is not universal across tumors.

Activating mutations of *KRAS* and loss of *TP53* dominate the PDAC genetic landscape^2^, and we have previously generated genetically engineered mouse models and derivative PDAC cell lines to study PDAC tumorigenesis^24^. Leveraging these genetically defined tools, we further investigated the role of Prmt1 in *LSL-Kras*^*G12D*^, *Tp53*-deficient mouse models. In two independent *LSL-Kras*^*G12D*^ *Tp*^*53*L/+^ mouse PDAC cell lines, we confirmed modulation of Prmt1-dependent arginine methylation comparable to human PDAC models (Supplementary Fig. 14a), as well as significant suppression of in vitro cell growth, upon Prmt1 inhibition (Supplementary Fig. 14b and c), suggesting a similar phenotype in both human and mouse PDAC models. To complement these in vitro data, *LSL-Kras*^*G12D*^ *Tp53*^*L/+*^ cells were implanted subcutaneously into immunodeficient mice. Although the allograft model displayed extremely aggressive growth, PRMTi induced significant TGI at tolerated doses (%TGI at 30 mg/kg = 60, *p* < 0.0001; %TGI at 100 mg/kg = 63; *p* < 0.0001; Supplementary Fig. 14d and e), consistent with observations in human PDAC models. Dose-dependent accumulation of MMA, indicative of Prmt1 modulation, was also confirmed in the murine tumors (Supplementary Fig. 14f). Taken together, these data support PRMT1 as a critical dependency in PDAC and prompted further evaluation of the biological mechanism of response.

### PRMT1 regulates a network of substrates involved in RNA metabolism

**Fig. 3:**
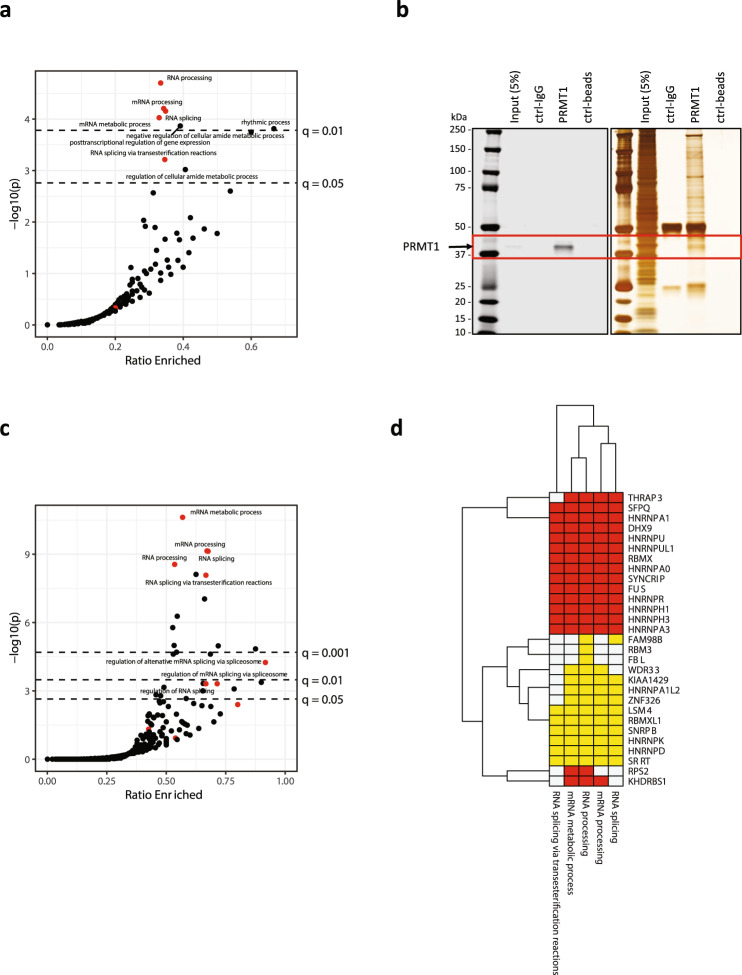
Proteins involved in RNA metabolism emerge as preferential PRMT1 substrates and binding partners. **a** Analysis of PTMScan data from PATC53 cells treated with 1 µM PRMTi or DMSO control for 24 h. Enrichment analysis for gene ontology (GO) biological processes were conducted using Fisher’s exact test for proteins with MMA enrichment upon treatment vs. all proteins analyzed. The *x*-axis indicates the ratio of enriched vs. total genes analyzed per GO term. Dashed lines indicate FDR-adjusted significance thresholds at *q* = 0.05 and *q* = 0.01. GO terms associated with RNA splicing or processing highlighted in red, and four of the five splicing/processing sets were enriched with *q* < 0.05. **b** PRMT1 Western Blot (left) and Silver Staining (right) images of proteins isolated from PATC53 cells (input) and immunoprecipitated with anti-IgG or anti-PRMT1 antibodies. The red square denotes PRMT1 protein. **c** Enrichment analysis of PRMT1 IP-MS data in PATC53 cells. RNA processing and splicing terms are denoted with red points, all others are black. Dashed lines indicate FDR-adjusted significance thresholds at *q* = 0.05, q = 0.01, and *q* = 0.001. Eight of eleven RNA processing/splicing terms are enriched with *q* < 0.05. **d** PTMScan MMA analysis (yellow) shows enrichment of RNA processing gene sets, many of which are supported by both IP-MS and PTMScan data (red). Source data are provided as a Source Data file.

To investigate the molecular mechanisms driving PRMT1-mediated inhibition of tumor growth in PDAC, we first employed a mass spectrometry-based proteomics approach (PTMScan^R^)^25,26^ to identify substrates methylated by PRMT1. Monomethylated and asymmetrically di-methylated peptides from PATC53 cells treated with PRMTi were enriched by immunoaffinity purification using mono-methyl arginine or asymmetric di-methyl arginine antibodies and then resolved by liquid chromatography-tandem mass spectrometry (LC-MS/MS) for identification and quantitative profiles. This analysis revealed a high number of relative changes in peptide abundance between treated and control conditions (Supplementary Fig. 15a and b) (Supplementary Data 4), which were indicative of PRMT1-dependent methylation events. Analysis of the MMA-enriched dataset revealed that 396 out of 943 peptides were differentially monomethylated. To gain insight into the potential cellular functions of PRMT1 substrates, we performed Gene Ontology (GO) analysis after mapping peptides back onto their protein of origin. From 381 proteins, we identified 69 proteins with increased monomethylation of at least one peptide upon treatment with PRMTi. These differentially monomethylated proteins were significantly enriched in nine GO biological processes (*q* < 0.05) corresponding to RNA splicing, RNA metabolism, and rhythmic processes, with the most enriched set represented by RNA processing (*q* = 0.004, 30/87 RNA processing genes enriched) (Fig. 3a). The enrichment for genes involved in RNA processing was also detected upon analysis of the ADMA-enriched dataset (*q* = 0.08, 12/39 genes) (Supplementary Fig. 15c). Many of these differentially methylated targets were RNA-binding proteins (RBPs), including multiple heterogeneous nuclear ribonucleoproteins (hnRNPs) and other proteins harboring glycine-arginine rich (GAR) domains, that are involved in RNA metabolism^27^. This is consistent with prior studies describing arginine methylation as a predominant post-translational modification of RBPs^26,28‒30^. Our dataset also identified proteins previously described to be methylated by PRMT1, such as hnRNPA1^31^, hnRNPK^32^, and hnRNPUL1^33^, thus increasing confidence in our experimental results.

RNA binding proteins regulate multiple aspects of RNA metabolism and form large ribonucleoprotein complexes; thus, we next evaluated whether PRMT1 physically interacts with specific protein complexes. To characterize the PRMT1 interactome, we coupled endogenous PRMT1 immunoprecipitation with LC-MS/MS (Fig. 3b), which identified 247 interacting partners (Supplementary Data 5). These genes encoding the protein interacting partners were over-represented in GO terms for 26 biological processes (Fig. 3c). The most enriched gene sets included mRNA metabolic processes (*q* < 0.001, 91/160 genes) and mRNA processing (*q* < 0.001, 51/76 genes). Overall, the observed enrichment was largely consistent between platforms, as over half of RNA processing genes enriched in the PTMScan analysis were also enriched in the immunoprecipitation-coupled LC-MS/MS analysis (Fig. 3d). Notably, enriched proteins included many hnRNP proteins that constitute a family of RBPs involved in alternative splicing, mRNA stabilization, and transport, as well as regulation of transcription and alternative mRNA polyadenylation^34‒36^.

### PRMT1 inhibition triggers widespread loss of expression of cell cycle, replication, and DNA repair genes

**Fig. 4:**
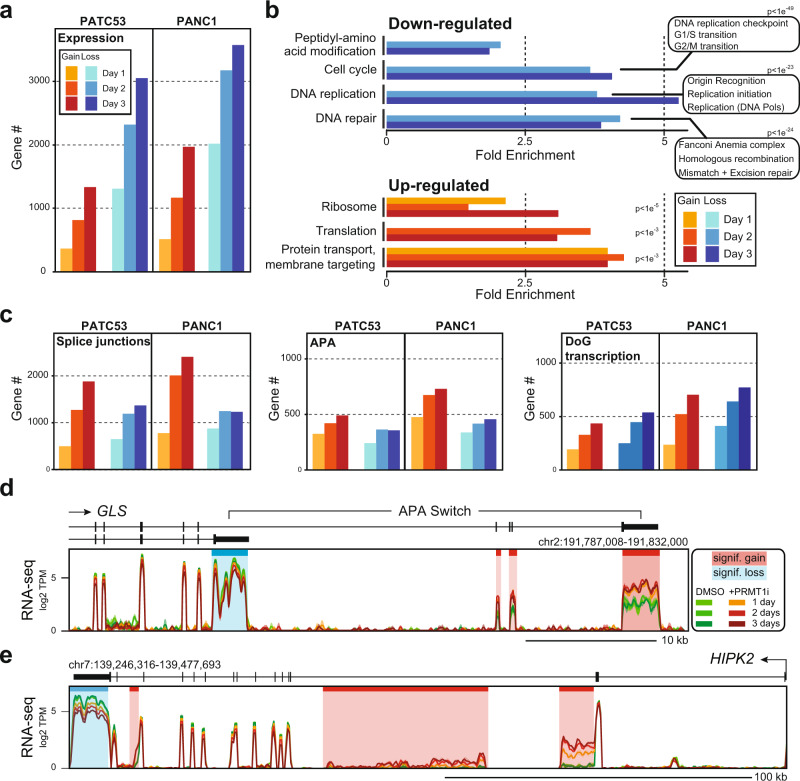
PRMT1 plays a critical role in regulating gene expression and co-transcriptional RNA processing. **a** Number of genes showing significant gain or loss of gene expression in PATC53 and PANC1 cells treated with 1 µM PRMTi for 1, 2, and 3 days. **b** Significantly enriched gene ontology (GO) categories (PANTHER) for down or upregulated genes as a function of time after PRMTi treatment in the cells described in (a). Key GO categories and p-values of enrichment are indicated. **c** Number of genes showing significant gain or loss of splicing junctions, alternative polyadenylation (APA), and downstream of gene (DoG) transcription events in the cells described in (**a**). **d** Representative composite screenshots of RNA-seq data over *GLS*, a gene that undergoes a treatment- and time-specific APA switch between two annotated 3’-UTRs, in PANC1 cells. **e** Representative composite screenshots of RNA-seq data over *HIPK2*, a gene that similarly to *GLS* undergoes significant APA gains over previously unannotated 3’-UTRs (shaded red) and concomitant loss of distal 3’-UTR usage (shaded blue), in PATC53 cells. Data from control and PRMTi-treated cells are color-coded as indicated.

To better understand how PRMT1 regulates RNA metabolism, we performed transcriptome analysis in PATC53 and PANC1 models after one, two, or three days of pharmacological PRMT1 inhibition. Gene expression was significantly perturbed, and a time-dependent increase in affected gene numbers was observed (Fig. 4a). Interestingly, the majority of transcriptomic changes corresponded to gene expression loss, and the responses observed in PATC53 and PANC1 cell lines were highly congruent. Analysis of GO terms underlying the deregulated genes revealed a strong enrichment for specific gene categories highly relevant to the cellular phenotypes triggered by PRMT1 inhibition. Specifically, we observed a strong downregulation of genes in pathways related to cell cycle, DNA replication, and DNA repair after two and three days of PRMT1 inhibition (Fig. 4b). This included many genes such as cyclins, cell division control proteins, and cyclin-dependent kinases that function to ensure high fidelity cell division (Supplementary Data 6). Downregulation of numerous genes involved in origin recognition, DNA replication initiation, and DNA synthesis were responsible for the emergence of DNA replication ontologies. Finally, a large number of genes involved in DNA repair and homologous recombination, including the entire suite of Fanconi Anemia genes, were also downregulated (Supplementary Data 6). Thus, consistent with our phenotypic data, PRMT1 inhibition triggered a dramatic downregulation of pathways required to maintain normal cellular growth (Figs. 1–2). Pathway analysis of genes upregulated after one day of PRMTi identified genes encoding 40S and 60S ribosomal proteins and complexes involved in protein trafficking (Fig. 4b). This suggests that cells may respond to PRMT1 inhibition by upregulating translation capacity. Transcriptomic analysis was also performed in a non-responder cell line, CFPAC1 (Fig. 2c). Consistent with PATC53 and PANC1 models, thousands of genes displayed treatment-specific and time-dependent up and downregulation, but with the majority of genes undergoing events of expression gain (Supplementary Fig. 16a). In striking contrast to observations in the PRMTi-sensitive cell lines, PATC53 and PANC1, GO analysis in CFPAC1 cells did not uncover significant enrichment in any specific functional pathway, with a weak GO enrichment for replication fork progression observed only after three days of treatment (Supplementary Data 6). These results suggest that the downregulation of cell cycle, DNA replication, and DNA repair genes may represent a specific mechanism conferring response to PRMT1 inhibition.

### PRMT1 is required for proper RNA splicing and 3’-UTR usage

PRMT1 methylates and binds to multiple RBPs that regulate RNA splicing, 3’-end RNA processing, as well as RNA stability and localization (Fig. 3). To understand the role of PRMT1 in regulating RNA metabolism, we annotated splice junctions and identified significant up- or down-regulation in junction usage in PATC53 and PANC1 cells. Thousands of genes experienced splicing changes upon PRMT1 inhibition, with a trend towards junction gains indicative of increased usage of alternative exons (Fig. 4c and Supplementary Data 6). Genes experiencing splicing perturbations were enriched for specific GO terms that, in many cases, overlapped with those identified upon surveying gene expression levels (Supplementary Fig. 16b). Cell cycle-related genes, for instance, showed both junction gains and losses, indicative of widespread splicing disruption. Interestingly, cell cycle and DNA repair genes showed significant enrichment for splice junction perturbations as early as one day after treatment, which preceded their observed reduction in gene expression at days two and three post-treatment (Fig. 4b). Thus, PRMT1 inhibition triggers widespread splicing deregulation, which is consistent with our observations that PRMT1 protein interactors and substrates include numerous alternative splicing regulators (Fig. 3a and c).

Detailed RNA-seq analysis revealed that PRMT1 inhibition was associated with additional abnormal RNA processing events. For example, time-dependent read count changes indicated that *GLS*, which encodes the metabolic enzyme glutaminase, switched from using proximal 3’-UTR to a distal one upon treatment with PRMTi (Fig. 4d). Independent RT-qPCR quantification confirmed that the frequency of the switch was significant as early as one day post-treatment, causing a two-fold reduction of the proximal 3’-UTR usage and a concomitant four-fold gain of the distal 3’-UTR usage (Supplementary Fig. 17). A similar alternative polyadenylation switch between these two annotated 3’-UTRs has been previously demonstrated for *GLS* in the context of tumorigenesis and is controlled by the CFIm25 (*NUDT21*) RBP, a subunit of the cleavage and polyadenylation machinery^37,38^. Similar patterns of time-dependent accumulation of RNA-seq read counts over intronic portions and reduced distal 3’-UTR usage were observed for hundreds of genes. For instance, the *HIPK2* gene, which encodes a kinase that regulates cell cycle and apoptosis^39^, showed treatment-specific and time-dependent RNA-seq signal accumulation over two regions of intron 2 and one region of intron 13, as well as reduced use of the most distal 3’-UTR (Fig. 4e). RT-qPCR assays validated such changes and revealed an up to 6-fold increased usage of intronic regions, concomitant with a two-fold reduction in the distal 3’-UTR usage (Supplementary Fig. 18). While the intronic signals in *HIPK2* did not map to annotated alternative polyadenylation (APA) sites, they shared characteristics of alternative 3’-UTRs. Specifically, these intronic signals only covered portions of introns, distinguishing them from simple events of intron retention, and no splice junctions joining these events to downstream exons could be identified, suggesting they marked the end of a transcript. We therefore propose that these intronic signals in *HIPK2* arose from APA events associated with premature transcription termination induced by PRMT1 inhibition.

To assess the degree to which APA contributes to the response of PDAC cells to PRMT1 inhibition, we systematically annotated such events in PATC53 and PANC1 models. Several hundred genes showed evidence of PRMTi-induced APA (Fig. 4c), many of which highlighted novel putative 3’-UTRs. Importantly, gene expression was significantly downregulated for approximately one-third of genes in which gains of APA were annotated after three days of treatment (Supplementary Fig. 16c), suggesting that premature cleavage and polyadenylation are important contributors to the response to PRMT1 inhibition. These observations are consistent with our findings that numerous hnRNP family members with roles in alternative 3’-end processing are direct targets and substrates of PRMT1 (Fig. 3). In addition to APA, we also noted that hundreds of genes showed “downstream of gene (DoG) transcription” in response to PRMT1 inhibition (Fig. 4c, Supplementary Fig. 16d). DoG transcription^40^ are events of read-through transcription caused by reduced transcription termination, as observed under stress conditions^41^. Similar to junction changes, genes undergoing APA or DoG transcription upon PRMTi treatment displayed enrichment for specific GO terms, several of which coincided with gene classes undergoing perturbations in gene expression levels (Supplementary Fig. 16b).

A similar analysis was conducted for the CFPAC1 nonresponder cell line. PRMT1 inhibition also triggered splicing disruptions and APA events, although splicing disruptions were relatively less abundant and far fewer events of 3’-UTR loss were observed compared to sensitive models (Supplementary Fig. 16a). A similar fraction of genes undergoing splicing disruption or APA events displayed loss of gene expression in all three models (Supplementary Fig. 16c and e). However, in contrast to the response observed in PATC53 and PANC1 cells, the response in CFPAC1 cells did not particularly affect genes involved in cell proliferation and DNA repair (Supplementary Data 6). This suggests that, while PRMT1 inhibition triggers similar RNA processing disruptions across cell lines, the anti-proliferative response relies on the perturbation and loss of expression of specific gene networks related to cell cycle control and DNA replication. Overall, our transcriptome analysis suggests that the effects of PRMT1 inhibition are largely due to alterations in co-transcriptional RNA processing at the level of splicing, cleavage and polyadenylation, as well as termination. Given that PRMT1 physically interacts with and post-translationally modifies many protein factors involved in splicing and 3’-UTR usage, this offers a direct mechanism by which PRMT1 may regulate gene expression.

### PRMT1 regulates key pathways involved in cell proliferation and DNA replication

**Fig. 5:**
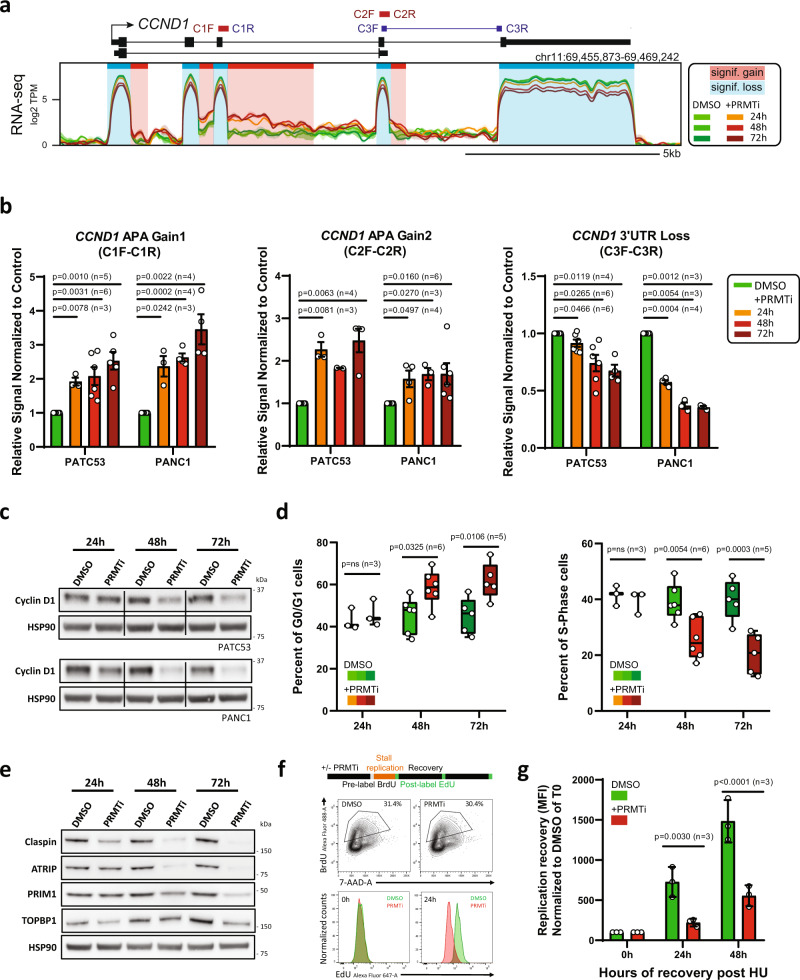
PRMT1 regulates key pathways involved in cell proliferation and DNA replication. **a** Representative composite screenshot of RNA-seq data over *CCND1*, a gene that undergoes significant alternative polyadenylation gains (shaded red) and concomitant expression loss (shaded blue) in PANC1 cells treated with 1 µM PRMTi or DMSO control. **b** RT-qPCR validation of alternative polyadenylation in the *CCND1* gene using primers indicated in (a) in PATC53 and PANC1 cells treated for 24 h, 48 h, or 72 h with 1 µM PRMTi or DMSO control. Data are presented as the mean ± SEM and *p* values are calculated by two-tailed Student’s *t*-test compared to DMSO-treated cells (n=independent experiments). **c** Western Blot analysis of Cyclin D1 in PATC53 and PANC1 cells treated with 1 µM PRMTi or DMSO control for indicated times. **d** Cell cycle profile of PATC53 cells treated with 1 µM PRMTi or DMSO control. Percentage of cells present in each cell cycle phase assessed by EdU or BrdU staining. Box-and-whisker plots in the panels depict 25–75% in the box, whiskers are down to the minimum and up to the maximum value, and median is indicated with a line in the middle of the box; *p* values are calculated by 2-way ANOVA compared to DMSO treated cells for each time point (*n*=independent experiments). **e** Western Blot analysis of indicated proteins in PATC53 cells treated with 1 µM PRMTi for the indicated time. HSP90 is shown as the representative loading control. **f** Replication restart in PATC53 cells treated with 1 µM PRMTi or DMSO control was evaluated by dual-labeling flow cytometry. Top panel: schematic representation of an experimental design. Middle panel: BrdU gating to select replicating cells. Bottom panel: plot showing replication restart as detected by EdU incorporation in BrdU positive cells immediately (0 h) and 24 h (24 h) after HU washout. **g** EdU incorporation in BrdU-positive cells post HU treatment was monitored by flow cytometry. Mean fluorescence intensity (MFI) of EdU is reported and normalized to 100 for PRMTi treated samples and controls at the time of HU washout (0 h). Data are presented as the mean ± S.D. and *p* values are calculated by 2-way ANOVA compared to DMSO treated cells for each time point (*n*=independent experiments). Source data are provided as a Source Data file.

Because cell cycle-related genes were among the most significantly affected (Fig. 4b and Supplementary Fig. 16b), we further evaluated the effect of PRMT1 inhibition on cell cycle progression. We observed that PRMT1 inhibition in responder cell lines triggered multiple APA events over the *CCND1* gene (Fig. 5a), which encodes the critical G1/S cyclin D1 and is known to undergo APA^42^. RT-qPCR assays confirmed that PRMT1 inhibition caused a significant reduction of the full-length isoform in favor of truncated forms in both responder cell lines (Fig. 5b). The use of these premature 3’-UTRs eventually induced robust loss of cyclin D1 expression at both the RNA and protein levels (Figs. 5b and 5c). Consistent with these changes, we observed a significant accumulation of cells in G0/G1 and a simultaneous reduction of cells in S phase starting at 48 h post-treatment (Fig. 5d and Supplementary Fig. 19a). These findings indicate that PRMT1 inhibition triggers defects in cell cycle progression. Importantly, no change in APA or gene expression was observed for *CCND1* in the CFPAC1 cell line (Supplementary Fig. 19b).

To address whether gene expression alterations observed upon PRMT1 inhibition were secondary effects to cell cycle arrest, we profiled gene expression changes of a subset of DNA replication genes starting 24 h post-PRMT1 inhibition, when no significant effect on cell cycle was observed (Fig. 5c). As shown for *CCND1* (Fig. 5b), RT-qPCR data revealed significant downregulation of key DNA replication genes as early as 24 h post-treatment (Supplementary Fig. 19c). Robust time-dependent modulation of these factors was confirmed at the protein level (Fig. 5e), suggesting that gene expression changes precede cell cycle defects and drive the anti-proliferative phenotype. Because PRMT1 inhibition impinges on the expression of DNA replication genes in responder cell lines, we investigated the effect of PRMTi treatment on the ability of replication forks to respond to severe replicative stress. For this, we leveraged a dual-labeling approach^43^ in which actively replicating cells were first labeled with BrdU and then treated with the fork stalling agent hydroxyurea (HU). Active fork recovery or new origin firing was monitored over time by EdU incorporation in BrdU-positive cells (Fig. 5f, top panel). Significant defects in EdU incorporation after HU washout were observed in PRMTi-treated cells compared to controls (Fig. 5f bottom panel and 5 g), suggesting that PRMT1 inhibition impairs replication restart.

### PRMT1 coordinates expression of DNA damage genes and maintains genome stability

**Fig. 6:**
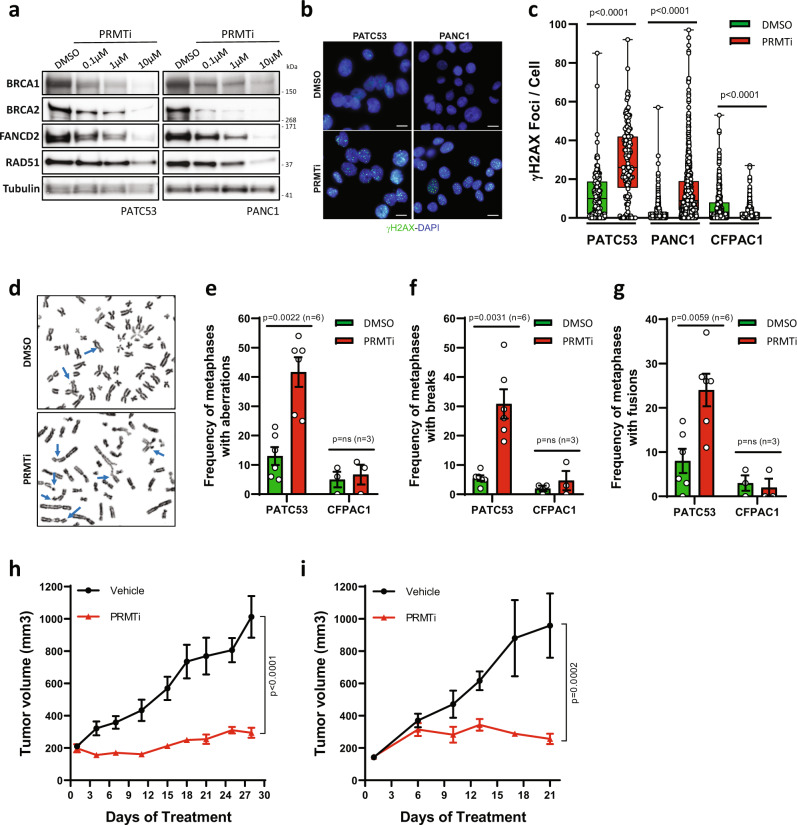
PRMT1 coordinates the expression of DNA damage genes and maintains genome stability. **a** Western Blot analysis of indicated proteins in PATC53 and PANC1 cells treated with PRMTi at different concentrations or with DMSO control. Tubulin is shown as the representative loading control. **b** Representative anti-γH2AX immunofluorescence in PATC53 and PANC1 cells treated with 1 µM PRMTi or DMSO control for 72 h (green: γH2AX; blue: DAPI). Scale bar 10 µm. **c** Box-and-whisker plot showing the distribution of the number of γH2AX foci/cell in PATC53, PANC1, and CFPAC1 cells treated with 1 µM PRMTi or DMSO control for 72 h. The panels depict 25–75% in the box, whiskers are down to the minimum and up to the maximum value, and the median is indicated with a line in the middle of the box. Data presented are from *n* = 180 (DMSO) and *n* = 145 (PRMTi) PATC53 treated cells examined over 2 independent experiments; *n* = 735 (DMSO) and *n* = 659 (PRMTi) PANC1 treated cells examined over 4 independent experiments; n = 504 (DMSO) and n = 565 (PRMTi) CFPAC1 treated cells examined over 3 independent experiments; *p* values are calculated by two-tailed student’s t-test compared to DMSO treated cells. **d** Representative images of Giemsa-stained metaphase spreads of PATC53 cells treated with 1 µM PRMTi or DMSO control for 7 days. Events are indicated by arrowheads. **e**–**g** Frequency of chromosomal aberrations reported as a percentage of total metaphases in PATC53 and CFPAC1 cells treated with 1 µM PRMTi or DMSO control for 7 days. The total number of metaphases with aberrations (**e**), number of metaphases with breaks (f), and number of metaphases with fusions (**g**) are shown. Data are presented as the mean ± SEM and *p* values are calculated by two-tailed Student’s t-test compared to DMSO controls (ns= non-significant; *n*=independent experiments). **h**–**i** Tumor growth curve (mm^3^) of PATX118 (**h**) and PATX45 (**i**) PDX models treated with PRMTi at 200 mg/kg QD, 5on/2off. Data are presented as the mean ± SEM and *p* values are calculated by 2-way ANOVA with multiple comparisons and Sidak correction, compared to vehicle control. For (h), *n* = 5 mice/group vehicle; *n* = 3 mice group PRMTi. For (i), *n* = 3 mice/group. Source data are provided as a Source Data file.

Replication restart involves multiple pathways, with key roles for multiple DNA repair genes, including *BRCA1/2*^44^, *RAD51*^45^, and *FANCD2*^46,47^. Given that our RNA-seq data revealed dramatic downregulation of pathways involved in DNA repair and homologous recombination (HR) upon PRMT1 inhibition (Fig. 4b), we tested whether these key genes also showed reduced expression at the protein level. In both PATC53 and PANC1 cells, PRMT1 inhibition resulted in a marked and dose-dependent reduction in levels of BRCA1/2, FANCD2, and RAD51 protein (Fig. 6a), which we also validated with individual RT-qPCR analysis (Supplementary Fig. 20a). PRMT1-dependent regulation of HR genes was independently confirmed upon CRISPR-Cas9-mediated PRMT1 silencing while, by contrast, PRMT4 and/or PRMT6 silencing showed little to no effect (Supplementary Fig. 20b). Consistent with these findings, we observed a significant accumulation of γH2AX in PATC53 and PANC1 cells, indicating DNA damage upon PRMT1 inhibition in vitro (Figs. 6b and 6c), and in tumors from animals treated with PRMTi (Supplementary Fig. 20c). Moreover, several chromosome abnormalities, including chromatid breaks and fusions, were observed on metaphase spreads from cells exposed to prolonged treatment with PRMTi, confirming the role of PRMT1 in maintaining chromosomal stability (Fig. 6d-g). Similar analyses were conducted with the non-responder CFPAC1 model. Consistent with the transcriptomic data (Supplementary Fig. 16a), PRMTi in CFPAC1 cells neither modulated expression of key DDR genes (Supplementary Fig. 20d) nor induced accumulation of DNA damage (Fig. 6c). By contrast, PRMTi in CFPAC1 cells led to a reduction in the γH2AX signal, suggesting that a potential compensatory mechanism is activated in CFPAC1 cells in response to PRMTi. In addition, no induction of chromosomal instability was observed upon PRMTi in CFPAC1 cells (Fig. 6e–g). Together, these data suggest that alterations in DNA replication and DDR gene expression, as well as DNA damage accumulation and genomic instability, are only observed in responder models and could define context-specific mechanisms of response to PRMT1 inhibition.

Given that PRMT1 inhibition leads to alterations of co-transcriptional RNA processing, DNA damage accumulation, and chromosome instability (Fig. 6b–g), we investigated whether PRMT1 regulates R-loop loads. R-loop structures form co-transcriptionally, and excessive R-loop formation has been invoked as a potential source of genomic instability, including in human cancers^48,49^. Using a novel variant of the DRIP-seq method^50^ to achieve strand-specific high-resolution R-loop profiling (Methods), we generated the first R-loop maps from PATC53 cells with and without PRMT1 inhibition. Treatment with PRMTi resulted in both gains and losses of R-loops (Supplementary Fig. 20e), with the latter dominating as R-loop loads were relatively reduced  in PRMT1-inhibited cells. In keeping with the co-transcriptional origin of R-loops, 68.4% of R-loop loss events mapped to genes for which expression was reduced by PRMTi (Supplementary Fig. 20f, right). A smaller proportion of R-loop gains overlapped with gene expression gains (Supplementary Fig. 20f, left). Interestingly, a significant proportion of genes undergoing APA also exhibited R-loop loss, as illustrated for the *HIPK2* gene (Supplementary Fig. 20g). R-loop loads over the gene fell off downstream of the novel, PRMTi-induced APA regions in a directional manner, supporting the notion that transcription terminates prematurely over these regions upon treatment. Similar observations were made for many other genes (Supplementary Data 6), which suggests that R-loop formation is a sensitive reporter of nascent transcription. However, the finding that PRMT1 inhibition yields an overall reduction in R-loop loads indicates that excessive R-loop formation is unlikely to be the cause of the associated genomic instability. Instead, genome instability is likely driven by the profound perturbations of the cell cycle and DNA replication, combined with a diminished DDR machinery, which is caused by PRMT1 inhibition.

Large-scale whole-genome sequencing has recently characterized the mutational landscape of pancreatic cancer, and these data have highlighted an emerging role for DDR aberrations in PDAC subtypes^3,51,52^. Given the impact of PRMT1 inhibition on multiple DDR networks, we evaluated the in vivo response to PRMT1 inhibition of PDAC PDX models that were deficient in DDR pathways. Models were selected by either the presence of somatic mutations in DDR genes or by being characterized with a high Homologous Recombination Deficiency (HRD) score using the Cosmic Signature 3. Strikingly, PRMTi treatment resulted in significant TGI in both the PATX118 model (Fig. 6h and Supplementary Fig. 21a), which harbors a nonsynonymous *BRCA2* mutation, and the PATX45 model (Fig. 6i and Supplementary Fig. 21b), which is characterized by a high HRD score. These data suggest that DDR-deficient PDAC tumors may be innately sensitive to PRMT1 inhibition, although the further investigation will be required to validate this translational path.

Our data demonstrate that PRMT1 coordinates the expression of key genes involved in the regulation of cell cycle progression, DNA replication, and DDR, required to maintain tumor growth and to promote genomic stability. Taken together, we identified PRMT1 as a novel vulnerability that may be exploited therapeutically for the treatment of pancreatic cancer.

## Discussion

In this study, we identified PRMT1 as a novel vulnerability in PDAC using our in vivo PILOT screening platform to identify genetic drivers of tumorigenesis. The dependency of PDAC on PRMT1 was confirmed through both genetic and pharmacological approaches. Specifically, PRMT1 knock-down and inhibition reduced cellular proliferation and was accompanied by the accumulation of DNA damage and genome instability. These findings were observed in cell line and PDX-derived models both in vitro and in vivo. Thus, PRMT1-catalyzed ADMA is required across multiple PDAC models to maintain tumor growth. These findings are consistent with previous work showing that loss of PRMT1 expression in mouse embryonic fibroblasts induced defects in cell cycle progression and DNA damage, as well as chromosomal abnormalities^53^.

To understand the mechanisms through which PRMT1 maintains cellular proliferation in sensitive PDAC models, we first leveraged proteomic studies to identify PRMT1 interacting partners and substrates. A congruent subset of RBPs and proteins involved in RNA processing were identified, consistent with other recent studies^22,29,54^. During transcription, nascent transcripts are immediately coated by multiple RBPs that dictate transcript maturation and fate by forming large messenger ribonucleoprotein particles called mRNPs^55^. The major post-translational modification of RBPs is the methylation of arginine residues, which regulates binding affinity to RNA^56^ and protein–protein interactions^9,27^. Alterations of RBP methylation status can thus contribute to aberrant mRNA biogenesis. Using transcriptomic approaches, we demonstrated that PRMT1 inhibition induces significant alterations in co-transcriptional RNA processing, including splicing changes, APA events, and termination defects. Importantly, our study revealed that PRMT1 inhibition in sensitive PDAC cells led to the downregulation of a specific gene subset involved in cell cycle progression, DNA replication, and DDR pathways, which is consistent with the cellular phenotypes triggered by PRMT1 inhibition.

Previous studies^57,58^ showed that DDR genes, including *BRCA1*, *BRCA2,* and multiple Fanconi anemia genes, were particularly sensitive to aberrant intronic polyadenylation events. Incomplete suppression of intronic polyadenylation led to the usage of internal APA sites, premature termination, as well as the production of truncated transcripts and shorter protein isoforms. Similar events were shown to occur upon PRMT1 inhibition at hundreds of genes, indicating that PRMT1 plays a critical role in regulating APA in PDAC. At least one-third of genes showing PRMTi-dependent APA showed gene expression loss, suggesting that 3’-UTR switching is accompanied by premature termination and/or negative feedback on transcription. PRMT1 inhibition, in particular, led to the reduced expression of FANCD2, BRCA1, and BRCA2 proteins, which contribute to the protection and restart of stalled replication forks^44,46,59^. RAD51, which is required for HR-mediated repair of collapsed replication forks^45^, was also characterized by the impaired expression upon PRMTi. Given the well-documented role of oncogenes such as *KRAS* in inducing replication stress^60^, it is not surprising that *KRAS*-driven PDACs are vulnerable to the dysfunction of pathways that sense and respond to replication stress. The critical G1/S cell cycle regulator, *CCND1*, along with several DNA replication factors, also showed clear PRMTi-induced APA and loss of gene expression. As a result, we observed that responsive PDAC cells accumulated in G0/G1 and the number of PDAC cells were depleted in S-phase in treatment- and time-dependent fashion. Non-responsive PDAC models, such as CFPAC1, by contrast, did not trigger APA or gene expression loss in *CCND1*, suggesting that the ability to engage with cell cycle genes is key to the anti-proliferative response. Interestingly, APA was recently identified as an important pro-tumorigenic driver in PDAC^61^. Overall, our study confirms that the ability of PDAC to carry out APA represents a key vulnerability and that the aberrant deregulation of APA triggered by PRMT1 inhibition has a strong negative impact on tumorigenesis by impinging on the cell cycle, DNA replication, and DNA damage response pathways.

More generally, our study adds to a growing body of work showing that RBPs are major players in the maintenance of genome stability and are directly involved in the DDR^62‒64^. Arginine methylation was shown to directly regulate the role of specific RBPs in repair mechanisms, as reported for hnRNPK^32^ and hnRNPUL1^33,65^, which were both identified as PRMT1 substrates in our proteomic analysis. Taken together, our findings suggest that PRMT1 is required to ensure the proper activity of multiple DDR-related pathways via its ability to post-translationally modify a variety of key RBPs. While our work highlights that PRMT1-mediated APA regulation is an important mechanism by which PRMT1 exerts its effects, our data suggest that PRMT1 also impacts other aspects of co-transcriptional RNA processing, such as RNA splicing. Specifically, PRMTi treatment caused significant splicing deregulation for a subset of genes with functions in cell cycle control, DNA replication, and DNA repair, among others. PRMTi-induced splicing changes are expected to lead to aberrant expression of protein isoforms and, in the case of intron retention events, to activate the nonsense-mediated decay response. Splicing changes were observed early on during PRMTi treatment, often preceding any change in gene expression, suggesting that RNA processing disruptions drove the cellular response. Interestingly, BCLAF1 and THRAP3 RNA processing factors were identified as PRMT1 substrates (Supplementary Data 4) and binding partners (Supplementary Data 5), and we also observed downregulation of BCLAF1 upon PRMT1 inhibition. Both factors were recently shown to be critical for the processing and export of a subset of transcripts encoding key members of the Fanconi anemia and BRCA-related pathway^66^. This suggests that PRMT1 inhibition may also affect the RNA processing and export function of BCLAF1/THRAP3, thereby impacting the expression of DDR genes.

It is worth noting that the PRMTi response was not solely characterized by reduced gene expression. Indeed, we also observed marked upregulation of pathways related to translation regulation upon PRMT1 inhibition. This observation is consistent with a previous study demonstrating that arginine methylation of ribosomal RBPs is associated with repression of mRNA translation^67^. Here, PRMT1 inhibition and the resulting reduction in arginine methylation led to the up-regulation of a specific gene set involved in ribosome biogenesis, translation, and protein trafficking, likely as an attempt to enhance translation efficiency to compensate for decreased mRNA pools.

The excessive accumulation of R-loop structures has been invoked as contributing to genome instability^48,49^. Yet, here, we show that R-loop loads were decreased by PRMTi, consistent with the reduction of gene expression observed over many genes and with the patterns of APA and early termination induced by PRMT1 inhibition. This most likely excludes R-loop structures as a major source of DNA damage and genomic instability in PDAC. We propose that, instead, PRMT1 promotes genome stability through a combination of molecular and cellular roles that include the regulation of RNA processing and expression of genes endowed with the ability to safeguard proper DNA replication and repair pathways.

Recent data suggest that the activity of PRMT1 may be pleiotropic, with context-specific regulation of core biological networks that can serve as defined molecular predictors of PRMT1 dependency^11^. For example, tumors that harbor chromosomal deletions of the methylthioadenosine phosphorylase gene (*MTAP*)^22^ or leukemias that harbor somatic alterations in core splicing factors^29^ have recently been described as being dependent on arginine methylation. Here, through comprehensive mechanistic studies, we show that PRMT1 inhibition globally impacts multiple DDR networks and induces genome instability, prompting us to propose a differentiated translational hypothesis supported by the mutational landscape of pancreatic tumors that highlights an emerging role for DDR aberrations in PDAC subtypes^4,51^. Building on the concept of synthetic lethality (for example, poly(ADP-ribose) polymerase (PARP) inhibition in HR-deficient tumors^68,69^; ATR inhibition in ATM-null tumors^70‒72^), PRMT1 inhibition may potentially serve as a new therapeutic paradigm to target a wide array of human tumors with defective DDR pathways or tumors that are characterized by enhanced genomic instability. In addition, our results suggest that PRMTi may potentially be exploited therapeutically with combination strategies to enhance the activity of DDR inhibitors that are either FDA approved or under clinical investigation. We provide initial evidence showing in vivo response to PRMT1 inhibition in PDAC models harboring mutations in key DDR genes or that are characterized by high HRD scores; however, further studies are warranted to refine a clinical biomarker and combination strategy.

In summary, our findings reveal the critical role of PRMT1 in pancreatic tumor maintenance to support a complex network of mechanisms that ensure cell cycle progression and genomic stability. Defining a novel mechanism of action of PRMT1 in PDAC, our pre-clinical data uncover a new translational hypothesis to guide the clinical development of PRMT1 inhibitors.

## Methods

### Cell lines

Patient-derived samples were obtained from consented patients under an Institutional Review Board (IRB)-approved protocol LAB07-0854 chaired by J.B.F. (UTMDACC). Patient-derived models PATC53, PATC124, PATC148 and PATC153 were generated as previously described^5^ and kindly provided by Dr Jason Fleming and Dr Michael Kim (MDACC). The cells were routinely maintained in DMEM/F12 Medium (Corning #10-090-CV) supplemented with 10% fetal bovine serum (Sigma #F2442) in a humidified incubator (37 °C, 5% CO_2_). *LSL-Kras*^*G12D*^ *p53*^*L/+*^ mouse PDAC cell lines were kindly provided by Dr Haoqiang Ying and cultured in RPMI 1640 (Gibco #72400-047) supplemented with 10% fetal bovine serum. PK-59 cell line was obtained from Riken Cell Bank and maintained in RPMI 1640 (Gibco #72400-047) supplemented with 10% fetal bovine serum. KP-3 and KP-4 cell lines were obtained from JCRB Cell Bank. Hup-T3 and Hup-T4 were obtained from Sigma. All other PDAC generic cell lines were obtained from ATCC and maintained following ATCC’s recommendations. Cell lines were validated by STR profiling and confirmed to be negative for mycoplasma.

### Functional genomics screens

In vivo functional genomics screens utilizing patient-derived xenografts were executed according to our PILOT platform^5^. PATC lines were infected at a multiplicity-of-infection (MOI) of 0.3 with a pooled shRNA lentiviral library targeting 237 chromatin-remodeling genes (10 independent shRNAs/gene). Upon infection patient-derived cells were transplanted at 3 × 10^6^−5 × 10^6^ cells per mouse ensuring an in vivo representation of 1000–2000 cells/barcode. Reference cells before injections and multiple tumor replicates were collected and processed for genomic DNA extraction, PCR-based barcode amplification and next-generation sequencing. Raw FASTQ files were filtered for a 4 bp spacer (CGAA) starting at 18th base allowing for one mismatch. We then extracted 23–40 bp of the above reads for targeting libraries, and 1–18 bp for a nontargeting library. Recognized barcode sequences were aligned using Bowtie2 (v2.0.2) to their respective libraries (2.35k Epigenome library and 2.7 k shRNA-empty library)^73^. Barcode counts by SAMtools (v0.1.19) were normalized for the amount of sequencing reads retrieved for each sample, using library size normalization. Hairpin fold changes in tumor samples were calculated by dividing the size-normalized counts by the size-normalized counts in the reference pool. These fold changes were transformed into a robust z-score by subtracting the median value and dividing by the median average deviation. The hairpin level z-scores were averaged across three biological replicates tumors, converted to percentiles for RSA analysis, and the 3 top-ranking hairpins for each gene were averaged as an alternative summary statistic. The average of the top 3 hairpins, the rank of the genes by this metric, the RSA logP-values and the rank of the genes by RSA logP are provided in Supplementary Data 2.

### Engineered cell lines development

*PRMT1* sh1 (Millipore-Sigma Lentiviral Mission TRC shRNA #TRCN0000290478; Seq: CCGGCAGTACAAAGACTACAA) and PRMT1 sh2 (seq: GGACATGACATCCAAAGAC^74^) were cloned into the constitutive pLKO.1-TRC cloning vector (Addgene: 8453) according to the manufacturer’s protocol. These same shRNA sequences were cloned into pENTR-THT (Addgene: 55790) and integrated into the inducible pGLTR-X-GFP-Puro (Addgene #58245) and Tet-Puro (Addgene #21915) backbones. For sgRNA experiments, Lentiviral CRISPR vectors (pCLIP-All-EFS-Puro) were ordered from transOMIC (Huntsville, Al) including a non-targeting control (cat# TELA1011), *PRMT1* (sgRNA #2, Tevh-1198183; sgRNA #3, Tevh-1265324), *PRMT4* (CARM1) (sgRNA #1 Tevh-108081; sgRNA #2, Tevh-1175223; sgRNA #3, Tevh-124365) and *PRMT6* (sgRNA #1, TEVH1103341, sgRNA #2, TEVH1170483; sgRNA #3, TEVH1237625). For the rescue experiment, shRNA-resistant cDNA to *PRMT1* Isoform 3 was designed by codon optimization and supplemental silent mutations in shRNA targeted regions. The cDNA fragment was synthesized by a custom kit on the BioXP 3200 System (SGI-DNA; San Diego, Ca) and cloned by a BP reaction (Invitrogen #11789100) into pDONR223 following the manufacturers protocol. The validated cDNA sequences were integrated into the lentiviral expression vector pLenti6.3/V5-Dest (Invitrogen #V53306) by an LR Gateway reaction. For viral transfections, the lentivirus packaging vectors used were psPAX2 (Addgene: 12260) and pMD2.G (Addgene: 12259). 293 T cells (ATCC) were cultured in DMEM (Gibco #10564-011) supplemented with 10% FBS (Sigma #F2442) and transfected using Lipofectamine 2000 (Invitrogen #52887) according to the manufacturer’s instructions. The viral supernatant was collected 48–72 h post-transfection, filtered through 0.45μm low protein binding filters (Corning) and stored at 4 °C. For transduction, fresh viral solutions were added to a cell culture medium containing 8 μg/mL polybrene (Millipore #TR-1003-6) and then replaced with fresh media after 24 h. Cells were then selected accordingly to the selection marker. All inducible lines were cultured in growth media supplemented with TET free FBS (Takara #631101).

### Western blot analysis

Proteins were extracted from cell cultures in lysis buffer containing: 20 mM Tris HCl (pH 7.4), 150 mM NaCl2, 15% glycerol, 1% TritonX-100, 1x Halt protease inhibitor cocktail (Thermo Scientific #78440), 1 mM Phenylmethanesulfonyl fluoride solution (PMSF) (Sigma #93482), 1% SDS, and 0.1% benzonase (Sigma #E1014). Proteins from tumors were extracted using ice cold RIPA buffer (Thermo Scientific #89900) supplemented with 2x Halt protease inhibitor cocktail (Thermo Scientific #78440) and 2X PMSF (Sigma #93482). Tumor extracts were homogenized with stainless steel beads (MedSupply Partners #NA-SSB16) in the Bullet Blender. Protein concentrations were determined using the DC Assay (BioRad). Samples were denatured in NuPAGE LDS sample buffer (Life Technologies) and either run on NuPAGE 4-12% Bis Tris gels with MOPS running buffer or NuPAGE 3-8% Tris-Acetate gels with Tris Acetate running buffer (Invitrogen). Gels were transferred using the Iblot2 system (Invitrogen). Antibodies used: PRMT1 (Cell Signaling Technology (CST) #2449, 1:1000), PRMT4/CARM1 (Bethyl #A300-421A, 1:2000), PRMT5 (CST# 2252, 1:1000), PRMT6 (CST #14641, 1:1000), Mono-Methyl Arginine (MMA-RGG) (CST #8711, 1:1000), Asymmetric Di-Methyl Arginine Motif (ADMA) (CST #13522, 1:1000), Cas9 (CST #14697, 1:1000), Cyclin D1 (CST #2978, 1:1000), BRCA1 (CST # 14823, 1:1000), BRCA2 (CST #10741, 1:1000), FANCD2 (CST #16323, 1:1000), Rad51 (CST #8875, 1:1000), Claspin (Novus Biologicals #NB100-248, 1:1000), ATRIP (Abcam #ab245632, 1:2000), PRIM1 (CST #4725, 1:1000), TOPBP1 (CST #14342, 1:1000), HSP90 (CST #4874, 1:1000), and β-Actin (Sigma #A1978, 1:2000). Membranes were either developed with chemiluminescence using HyGlo Quick Spray Kit (Thermo Fisher Scientific #NC9774344) on the Imagequant LAS 4000 (GE Healthcare) or on the fluorescence based LiCor Odyssey system.

### RNA isolation and quantitative RT-PCR

RNA isolation was performed using Qiashredders (Qiagen #79654) and the RNEasy Mini kit (Qiagen #74104), cDNA was generated using the SuperScript VILO cDNA Synthesis Kit (Invitrogen #11754250) and real-time PCR was performed with Qiagen primers and SYBR GreenER qPCR SuperMix Universal (Invitrogen # 11762500) following manufacturer’s instructions. Qiagen RT2 qPCR primers (product #330001): *BRCA1* - #PPH00322F-200, *BRCA2* - #PPH00321F-200, *FANCD2* - #PPH14413A-200, *RAD51* - #PPH00942F-200, ERCC4 - #PPH01736A-200, *FEN1* - #PPH00502B-200, ATRIP - #PPH66805A-200, *TOPBP1* - # PPH10470A-200, *CDC45* - #PPH00915A-200, *CLSPN* - #PPH13637A-200, *PRIM1* - # PPH60100A-200, and *GAPDH* - #PPH00150F-200 as a housekeeping control. Primers for alternative polyadenylation are reported on Supplementary Data 7.

### Long-term phenotypic assay

Engineered cells were seeded in 6 wells or 12 wells and incubated for 14 days at 37 °C with 5% CO_2_. For compound treatment, cells were seeded in 6 wells or 12 wells and incubated for 24 h at 37 °C at 5% CO_2_. The next day, stock solutions of the test compounds were prepared in 100% DMSO (Sigma #D2650) and serially diluted 1:3 using 100% DMSO. Compounds were additionally diluted in culture medium, and then transferred to the tissue culture plate. Following the compound addition, the plate was incubated at 37 °C and 5% CO_2_ for 14 days. At the endpoint, culture media was removed and the plate was incubated for 10 min with 0.1% crystal violet solution (Sigma #W3500) containing 10% ethanol. After washing three times with water, the plate was dried overnight and then scanned with Epson scanner. For signal quantification, crystal violet dye was solubilized from stained cells with 10% Acetic Acid and then OD read at 590 nm. Data were normalized to DMSO-treated control (CNT) samples, transformed and analyzed with nonlinear regression curve fit to generate IC50 values with GraphPad Prism.

### In vivo studies

All in vivo work was approved by the IACUC of the University of Texas at MD Anderson Cancer Center.

For xenograft and allograft studies, female CD-1 nude (Charles River) or female NSG (Jackson) between 6–12 weeks old were used as recipients. For PATC53 xenograft and LSL-KRAS 875 allograft studies, cells were harvested, counted and resuspended at 1 million cells/100 µl in PBS. The cell suspension was mixed 1:1 with matrigel and a total volume of 200 µl/mouse was injected subcutaneously in the right flank of immune-compromised mice. Tumor growth was first monitored with caliper and tumor volume (TV) calculated using a standard formula: (length × width^2^)/2. For genetic experiments, mice were randomized to either doxycycline diet (200 mg/Kg) or control chow upon tumor establishment (between 100–200 mm^3^). For pharmacological experiments, tumor volume was measured weekly and mice were allocated to different groups according to their tumor volume (between 100–200 mm^3^) to give homogenous mean and median tumor volume in each treatment arm. Treatments were randomly attributed and mice were treated as indicated for each study. The tolerability of the tested compound was evaluated by clinical sign observation and body weight measurement during treatment. For PDX models, tumor fragments were transplanted subcutaneously in NSG mice. Tumor volume was measured as described above and when tumors reached between 150–250 mm^3^, mice were randomized into experimental groups as indicated for each study. Treatment response was determined by percent tumor growth inhibition (%TGI), defined as the percent difference between final median tumor volumes (MTVs) of treated and control groups.

### Treatment agent

GSK3368715 (PRMTi) was prepared according to the described synthetic procedures^22^.

### CST PTMScan

Cells were cultured with 1 µM PRMTi or DMSO control for 24 h and then analyzed using the PTMScan method^25,75^. Briefly, cellular extracts were prepared in urea lysis buffer, sonicated, centrifuged, reduced with DTT, and alkylated with iodoacetamide. 15 mg total protein for each sample was digested with trypsin and purified over C18 columns for enrichment with the Mono-Methyl Arginine Motif Antibody (#12235) and Asymmetric Di-Methyl Arginine Motif Antibody (#13474). Enriched peptides were purified over C18 STAGE tips (Rappsilber), subjected to secondary digest with trypsin and second STAGE tip prior to LC-MS/MS analysis. Enriched peptides were resuspended for 3 injections and replicate injections of each sample were run non-sequentially for each enrichment. Peptides were eluted using a 90 min linear gradient of acetonitrile in 0.125% formic acid delivered at 280 nL/min. Tandem mass spectra were collected in a data-dependent manner with a Thermo Orbitrap Velos™ mass spectrometer using a top-twenty MS/MS method, a dynamic repeat count of one, and a repeat duration of 30 sec. Real-time recalibration of mass error was performed using lock mass (Olsen) with a singly charged polysiloxane ion m/z = 371.101237. MS/MS spectra were evaluated using SEQUEST and the Core platform from Harvard University (Eng, Huttlin, Villen). Files were searched against the SwissProt Homo sapiens FASTA database. Mass accuracy of + /−5 ppm was used for precursor ions and 1 Da for product ions. Enzyme specificity was limited to trypsin, with at least one tryptic (K- or R-containing) terminus required per peptide and up to four mis-cleavages allowed. Cysteine carboxamidomethylation was specified as a static modification, oxidation of methionine and mono- or di-methylation on arginine residues were allowed as variable modifications. Reverse decoy databases were included for all searches to estimate false discovery rates (FDR), and filtered using a 2.5% FDR in the Linear Discriminant module of Core. Peptides were also filtered for the presence of a mono- or di-methyl arginine residue. All quantitative results were generated using Progenesis (Nonlinear Dynamics) and Skyline (MacLean) to extract the integrated peak area of the corresponding peptide assignments. Accuracy of quantitative data was ensured by manual review in Skyline or in the ion chromatogram files.

### Immunoprecipitation LC-MS/MS assay

For PRMT1 immunoprecipitation, cells were lysed in cell lysis buffer containing 10 mM Tris-HCl pH 7.5, 150 mM NaCl, 1% Triton X-100, 5 mM EDTA, and a complete proteinase and phosphatase inhibitor cocktail. Lysates were centrifuged at 4 °C for 20 min at 13,523 g and then supernatant collected. Dynabeads® Protein A (cat# 10002D) were washed three times in lysis buffer and then incubated with PRMT1 antibody (Bethyl Laboratory, A300-722A) or IgG control (CST-#3900) for 10 min. After three washes in lysis buffer, the antibody-bound Dynabeads^®^ was incubated with lysates for 1 h at room temperature. Beads were then washed three times, collected for Western blot analysis and silver staining, and sent for LC-MS/MS analysis to MS Bioworks. Anti-PRMT1 antibody (Abcam, ab12189) was used for western blot analysis. Pulldown samples were eluted from the Dynabeads solid phase by heating at 60 °C in 60 µL of 1.5X LDS for 15 min. Elutions were clarified by centrifugation and using a magnet. 50% of each elution was processed by SDS-PAGE using a 10% Bis-Tris NuPAGE gel (Invitrogen) with the MES buffer system, the gel was run approximately 2 cm. The mobility region was excised into 10 equally sized bands. Bands were processed by in-gel digestion using a robot (ProGest, DigiLab) with the following protocol, washed with 25 mM ammonium bicarbonate followed by acetonitrile; reduced with 10 mM dithiothreitol at 60 °C followed by alkylation with 50 mM iodoacetamide at RT; digested with trypsin (Promega) at 37 °C for 4 h; quenched with formic acid and the supernatant was analyzed directly without further processing. Half of the digested sample was analyzed by nano LC-MS/MS with a Waters NanoAcquity HPLC system interfaced to a ThermoFisher Q Exactive. Peptides were loaded on a trapping column and eluted over a 75 µm analytical column at 350 nL/min; both columns were packed with Luna C18 resin (Phenomenex). The mass spectrometer was operated in data-dependent mode, with the Orbitrap operating at 60,000 FWHM and 17,500 FWHM for MS and MS/MS respectively. The fifteen most abundant ions were selected for MS/MS. Data were searched using a local copy of Mascot with the following parameters: Enzyme: Trypsin/P, Database: SwissProt Human (concatenated forward and reverse plus common contaminants), Fixed modification: Carbamidomethyl (C), Variable modifications: Oxidation (M), Acetyl (N-term), Pyro-Glu (N-term Q), Deamidation (N/Q), Mass values: Monoisotopic, Peptide Mass Tolerance: 10 ppm, Fragment Mass Tolerance: 0.02 Da, Max Missed Cleavages: 2. Mascot DAT files were parsed into Scaffold (Proteome Software) for validation, filtering and to create a nonredundant list per sample. Data were filtered using at 1% protein and peptide FDR and requiring at least two unique peptides per protein.

### Mass spectrometry data analysis

Manufacturer-recommended cutoffs for defining enriched proteins were used. For PTMscan data, enriched proteins peptides were defined as those with a 2-fold change and maximum intensity greater than 1,000,000, or 2.5-fold change and maximum intensity greater than 200,000 (maximum CV < 50% required in either case). For IP-MS data, enriched proteins were defined as proteins with at least 5 spectral counts in the treatment sample and no counts in control, or a ratio greater than 4 for treatment versus control (if detected in both). Overrepresentation of enriched proteins peptides in GO (biological processes) terms was calculated using Fisher’s exact test for both increased and decreased peptides, against a background of all analyzed proteins peptides (PTMScan) or all proteins detected in any PATC53 sample (IP-MS). Only GO terms with at least 10 genes were included in the analysis. For this analysis, proteins peptides were collapsed to gene symbols, and a gene was considered enriched if at least one of the corresponding proteins was enriched. In addition, enriched genes were intersected with the BioGRID database of PRMT1 interactors using Fisher’s exact test.

### R-loop mapping using sonication DRIP-seq (sDRIP-seq)

R-loop mapping was performed following the DRIP protocol^50^ with some modifications. PATC53 cells were treated with 1 µM PRMTi or DMSO as control. One and three days later, DNA was extracted as described^76^, but genome fragmentation was conducted by shearing via sonication using a Diagenode Bioruptor (12 cycles, High, 15’ON 90’OFF). As sonication degrades the single-stranded looped out DNA strand of R-loops, immunoprecipitation with S9.6 enriches mostly for two-stranded RNA: DNA hybrids. To build sequencing libraries, hybrids were transformed back into double-stranded DNA via a second-strand DNA synthesis step using E. coli RNase H1, DNA ligase, DNA polymerase I (New England Biolabs) and a dNTP mix in which dUTP was used instead of dTTP. After checking the quality of the immunoprecipitation by qPCR, the DNA was built into strand-specific sequencing libraries with a UDG DNA glycosylase step before the PCR amplification step to ensure strand specificity^76^. Library quality was checked on an Agilent BioAnalyzer and sequencing performed on an Illumina HiSeq4000 instrument. Mapping was performed on two independent biological replicates.

### Bioinformatics analysis

Raw reads were trimmed with fastq-mcf v2.4.4 and mapped with bowtie2 v2.2.6 (sDRIP-seq) or tophat2 v2.1.0 (RNA-seq), taking only primary aligned, uniquely mapped reads, and in the case of RNA-seq, properly-mapped read pairs. Read duplicates were removed using Samtools v0.1.19. sDRIP-seq peaks were called using a custom-built Hidden Markov Model^77^, and further combined across samples to obtain one uniform set of peaks such that the distribution of reads across these peaks could be measured and tested for all samples. For RNA-seq, we used the Gencode v19 (GRCh37.p13) Appris gene annotation set (http://appristools.org/#/downloads), prioritizing highest principal isoform for each gene. Tophat2-called junctions were also used in calling putative 3’UTR. Total read counts mapping to each region were determined using HTseq-count v0.6.0. DESeq2 v1.10.1 was used to identify differentially expressed genes and determine statistical significance, enforcing a minimal fold change of 2× up or down alongside an adjusted *p*-value of < 0.05. We used PANTHER version 14.1^78^ to analyze gene ontology enrichment for genes with significantly increased and decreased expression, splice junctions, and alternative 3’ isoforms (RNA-seq), or R-loop formation (sDRIP-seq). Default parameters were used and only significant GO terms (adjusted *P*-value < 0.05) were retained.

### Cell cycle analysis

PATC53 cells were treated with DMSO or 1 µM PRMTi and collected after 24, 48 or 72 h of treatment. Cells were pulse-labeled with EdU or BrdU for 2 h before collection and then stained with Click-iT EdU Alexa Fluor 647 assay kit (ThermoFisher #C10424) or FITC BrdU Flow Kit (BD Biosciences #559619) according to the manufacturer’s instructions. Flow cytometry was conducted on a BD LSR Fortessa instrument and data was analyzed using the FlowJo software.

### Replication restart assay

Replication restart assay was performed as previously described^43^. Briefly, PATC53 cells were treated with DMSO or 1 µM PRMTi for 24 h and then pulse-labeled with 10 µM BrdU for 1 h (BD Biosciences # 559619). Cells were then washed with pre-warmed media and replenished with fresh media containing 2 mM hydroxyurea (HU) (Sigma #H8627) and incubated for 12 h. Cells were then washed and pulse-labeled with 10 µM EdU for 1 h at 24 h and 48 h after HU removal using the Click-iT EdU Alexa Fluor 647 assay kit (ThermoFisher #C10424). Cells were trypsinized with 0.25% trypsin then washed with media and PBS. Cells were fixed and frozen according to the instructions in BrdU flow kit (BD Biosciences #559619). Cells were prepared for flow cytometry by following the manufacturer’s instructions (including staining with 7-AAD) for the BrdU kit (BD Biosciences #559619) and for the Click-iT EdU Alexa Fluor 647 assay kit (ThermoFisher #C10424). Flow cytometric analysis was performed on the LSR Fortessa X-20 and data was analyzed using the FlowJo software. DMSO control or PRMTi was maintained throughout the length of the experiment.

### Immunofluorescence and immunohistochemistry

FFPE blocks were sectioned (5 μm), mounted on charged microscope slides (Leica 38002092), and dried at 37 °C overnight. Slides were then baked at 60 °C for 1 h in an oven (Biocare DRY2008US), deparaffinized in 3 changes of xylene, and then rehydrated in 3 changes of 100% ethanol followed by a series of 95, 70, and 50% ethanol and distilled water (5 min. each). Antigen retrieval (10 mM sodium citrate, pH 6.0 with 0.05% Tween 20) was performed by heating slides to 95 °C for 15 min in a microwave (Biogenex EZ Retriever System v.3) followed by a 30 min cool down at room temperature. Slides were then washed with TBST (Thermo TA-999-TT). The area around each section was traced with a PAP pen (ImmEdge H-4000), blocked with Background Sniper (Biocare BS966) for 10 min, and then washed with TBST. A sequential incubation with fluorophore-conjugated primary antibodies was performed (rabbit anti-phospho-Histone H2A.X (S139) Alexa-647 conjugate, CST 9720, 1:100; rabbit anti-HLA A, Abcam ab52922, 1:25, conjugated to Alexa-594 Fab fragments, Thermo Z25370) via the manufacturer-recommended protocol; all these antibodies were diluted in Fluorescence antibody Diluent, “FAD” (Biocare FAD901L), for 1 h at room temperature, followed by a TBST wash. Finally, slides were incubated with Hoechst 34580, 0.2 μg/mL (diluted in TBS), life tech H21486, for 10 min at room temperature. Slides were then washed with a series of TBST, followed by TBS and then distilled H2O. Excess liquid was removed and slides were mounted with ProLong Diamond Gold, Life Tech P36930, and allowed to harden. Image acquisition and Analysis: slides were imaged on a Vectra 3 using the A UPlanSApo 10x/0.40 air objective first. Images were acquired using all available channels with the Vectra software (3.0.5) and the raw data was saved as “qptiff” files. Regions of interest (ROIs) were created using PhenoChart (1.0.10) and these areas were then imaged again at 20X magnification on the Vectra. 20x images were then spectrally unmixed using inForm software and saved as Component TIFFs. Files were opened in Fiji software using the BioFormats plugin. Channels were split and saved individually as TIFFs and then opened in Adobe Photoshop and merged.

For in vitro experiments, PATC53 and PANC1 cells (5000 cells/well) were plated on glass coverslips in a 24-wells plate and treated with DMSO or 1 µM PRMTi for 72 h. Then, cells were washed three times with PBS 1X and fixed using 4.0% formaldehyde for 20 min at room temperature. Fixed cells were washed with PBS 1X and permeabilized by using 0.2% Triton X-100 in PBS 1X for 10 min. After permeabilization, cells were washed 3 times with PBS 1X and then blocked using 5% goat serum albumin in PBS 1X for 60 min at room temperature. Cells were washed three times with PBS 1X and then were incubated with primary antibody (Anti-phospho-Histone H2AX #05-636 Millipore) diluted 1:100 in antibody dilution buffer overnight at 4 °C. Then, cells were washed three times with 1× PBS and incubated with an anti-mouse secondary antibody (Alexa Flour 488-Invitrogen) diluted 1:400 in antibody dilution buffer for 2 h at room temperature in the dark. After three washes with PBS 1X, cells were stained with DAPI (Invitrogen) for 10 min. Cells were then washed with PBS 1X and coverslips where mounted on the microscopy slides with antifade mounting medium (VECTASHIELD) for 24 h. Image acquisition and analysis: fixed images were captured with a Hamamatsu C11440 digital camera, using a wide-field Nikon Eclipse Ni microscope. An APP was created in Visiopharm to segment cells and identify foci. Raw analyzed data were exported as a.CSV file and graphed/statistics run using a combination of Origin and GraphPad PRISM 8 software.

For PRMT1 immunohistochemistry, 5 micron tissue sections were backed and deparaffinized, and antigen retrieval was performed in citrate buffer at 95 °C for 30 min. Endogenous peroxidases were inactivated by 3% hydrogen peroxide for 10 min and nonspecific signals were blocked using a Blocking reagent (Biocare BS966) for 10 min. Samples were then stained with PRMT1 rabbit antibody (Bethyl IHC-00106) 1:500 at room temperature for 30 min. Rabbit-on-Rodent HRP-Polymer (Biocare #RMR622) was used as rabbit secondary detection systems and DAB (Thermo Scientific #TA-060-QHDX) for detection. Slides were then scanned with Pannoramic 250 whole slide digital scanner.

### Analysis of chromosomal aberrations

Analysis of chromosomal aberrations was performed at the Molecular Cytogenetics Facility (MD Anderson Cancer Center) as previously described^79^. Briefly, PATC53 cells were treated with DMSO or 1 µM PRMTi for 7 days. Cells were then treated with colcemid (0.04 µg/mL) for 2 h at 37 °C, trypsinized and collected. After centrifugation at 350 × g for 10 min, cells were resuspended in hypotonic solution (0.075 M KCl) for 15 min at room temperature, fixed in methanol and acetic acid (3:1 vol/vol), and washed three times in the fixative. Air-dried preparations were made and slides were stained with 4% Giemsa. At least 35 metaphases were analyzed from each sample for chromosomal aberrations. Images were captured using a Nikon 80i microscope equipped with karyotyping software from Applied Spectral Imaging, Inc.

### PRMT1 enzyme assay

In order to measure PRMT1 enzymatic activity, the LANCE TR-FRET assay from PerkinElmer was used to follow the methylation of histone H4 at Arg3 using S-adenosyl-L-methionine (SAM) as the methyl group donor. This enzymatic assay was performed in a 384 well, white, low volume plate (PerkinElmer, Catalog 6008289) with assay buffer consisting of 50 mM Hepes (pH 8) (Teknova, Catalog #H1090), 1 mM TCEP (Sigma, Catalog #C4706), and 0.003% Tween-20 (Thermo, Catalog #85114). Stock solutions of the test compounds were prepared in 100% DMSO (Sigma, Catalog #D2650) and serially diluted 1:3 using 100% DMSO. Compounds were additionally diluted at 1:40 in assay buffer, and 2 µL/well were transferred to the assay plate. 4 µL/well (final concentration 1.5 nM) of PRMT1 protein (SignalChem, Catalog #P365-380G) diluted in assay buffer was added to the assay plate followed by a 15 min preincubation at room temperature. 4 µL/well of SAM (Sigma, Catalog #A7007) and biotinylated histone H4 (1-21) (AnaSpec, Catalog #62555) (final concentrations 1 µM and 25 nM, respectively) diluted in assay buffer were then added to the assay plate followed by a 1 h reaction time. Final concentrations of PRMT1, SAM, and histone H4 (1-21) refer to a 10µL volume. Detection of methylated histone H4 (H4R3me) was achieved by combining LANCE Ultra Europium-anti-H4R3me antibody (PerkinElmer, Catalog #TRF04-14), LANCE Ultra ULight-anti-streptavidin antibody (PerkinElmer, Catalog #TRF0102), and sinefungin (Sigma, Catalog #S8559) (final concentrations 2 nM, 50 nM, and 100 µM respectively) in 1× LANCE detection buffer (PerkinElmer, Catalog #CR97-100) and adding 10 µL/well of the detection solution to the assay plate. Detection reagents were allowed to react for 1 h at room temperature. Final concentrations in the detection solution refer to a 20 µL volume. The europium fluorescence signal and the ULight TR-FRET signal were measured using a BioTek Synergy Neo plate reader: excitation at 330 nm, emission at 620 nm and 665 nm respectively, and the ratio of the two signals (665 nm/620 nm) was used for curve fitting. IC50 values were calculated using a four-parameter logistic curve fit using Genedata Screener software.

### PRMT1 cellular target engagement assay

RKO cells were routinely maintained in EMEM media (ATCC, Catalog #30-2003) supplemented with 10% fetal bovine serum (Sigma, Catalog #F2442) using a humidified incubator (37 °C, 5% CO_2_, and ambient O_2_). In preparation for the In-Cell Western assay, cells were harvested and resuspended in EMEM media supplemented with 10% fetal bovine serum. Cells were seeded onto a 384 well, black, clear-bottom, Poly-D-Lysine coated tissue culture plate (Greiner, Catalog #781946) at a density of 1000 cells/well in a volume of 40 µL. The culture plate was incubated for 24 h at 37 °C with 5% CO_2_ and ambient O_2_. Stock solutions of the test compounds were prepared in 100% DMSO (Sigma, Catalog #D2650) and serially diluted 1:3 using 100% DMSO. Compounds were additionally diluted 1:40 in culture medium, and 10 µL/well was transferred to the tissue culture plate. Following the compound addition, the microplate was incubated at 37 °C for 48 h. The media was removed, the plate was washed with 1× PBS (Fisher Bioreagents, Catalog #BP399-20), and cells were fixed for 10 min using 30 µL/well of 4% paraformaldehyde (Electron Microscopy Sciences, Catalog #15710). The paraformaldehyde was removed, the plate was again washed with 1× PBS, and cells were permeabilized for 15 min using 30 µL/well of 1× PBS containing 0.5% Triton-X 100 (Sigma, Catalog #1001748095). The permeabilization buffer was removed, the plate was washed with 1× PBST (Boston BioProducts, Catalog #IBB-171X), and 50µL/well of blocking buffer (LI-COR, Catalog #927-40000) was added followed by a 1 h incubation at room temperature. The blocking buffer was removed, and 20 µL/well of anti-asymmetric di-methyl arginine antibody (Cell Signaling, Catalog #13522 S) diluted 1:1000 in LI-COR blocking buffer was added to the plate and incubated overnight, in the dark at 4 °C. The primary antibody was then removed, and the plate was washed three times with 1× PBST. 20 µL/well of CellTag (LI-COR, Catalog #926-41090) and IRDye 800CW goat anti-rabbit IgG antibody (LI-COR, Catalog #926-32211), each diluted 1:500 in LI-COR blocking buffer supplemented with 0.1% Tween-20 (Thermo Scientific, Catalog #85114), were added to the plate. The plate was then incubated in the dark, at room temperature for 1 h followed by three washes with 1× PBST and one wash with water. The IRDye secondary antibody signal (800 channel) and the CellTag signal (700 channel) were measured using a Licor Odyssey Imager, and the 800 channel signal was then normalized to the 700 channel signal. IC50 values were calculated using a four-parameter logistic curve fit using Genedata Screener software.

### PRMT 1, 4 and 6 rapidfire mass spectrometry selectivity assays

PRMTs catalyze the transfer of the methyl group from the cofactor S-5′-adenosyl-L-methionine (SAM) to arginine residues of a variety of histone and nonhistone proteins. The production of S-(5′-Adenosyl)-L-homocysteine (SAH) was measured using Agilent’s RapidFire 365-Agilent QQQ 6460 to assess selectivity between PRMT1, 4 and 6. Reactions were performed in a 384 well plate (Greiner, catalog #MPG-784201) with assay buffer consisting of: 50 mM TRIS pH 8.0 (Invitrogen, catalog #15568-025), 1 mM TCEP (Sigma #C4706-2G), and 0.0015% Tween-20 (Thermo Scientific, catalog #85114). Full-length human PRMT1 (1-361) was expressed in E. coli and purified. The PRMT1 assay was performed by dispensing 6 µL/well (final concentration of 2 nM) of PRMT1 protein diluted in assay buffer to the plate. Stock solutions of the test compounds were prepared in 100% DMSO (Sigma, Catalog #D2650) and serially diluted 1:3 using 100% DMSO. Compounds were additionally diluted 1:50 in assay buffer, and 6 µL/well was transferred to the assay plate. Plates were allowed to incubate for 15 min at room temperature. 12 µL/well of SAM (Cayman Chemical, Catalog #13956) and biotinylated histone H4 (1-21) (AnaSpec, catalog #62555) (final concentrations of 1 µM and 50 nM respectively) diluted in assay buffer, were added to the plate followed by a 20 min reaction time. A final reaction volume of 20 µL was quenched with the addition of 43 µL of 0.6% (w/v) trifluoroacetic acid solution (Sigma, catalog #302031). Full-length human GST-PRMT4 (SignalChem, catalog#P365-380DG) was expressed by baculovirus in Sf9 insect cells. The PRMT4 assay was performed by dispensing 6 µL/well (final concentration of 0.5 nM) of PRMT4 protein diluted in assay buffer to the plate. Stock solutions of the test compounds were prepared in 100% DMSO (Sigma, Catalog #D2650) and serially diluted 1:3 using 100% DMSO. Compounds were additionally diluted 1:50 in assay buffer, and 6 µL/well was transferred to the assay plate. Plates were allowed to incubate for 15 minutes at room temperature. 12 µL/well of SAM (Cayman Chemical, Catalog #13956) and Histone H3.3 (Reaction Biology, catalog #HMT-11-134) (final concentrations of 0.5 µM and 15 nM respectively) diluted in assay buffer, were added to the plate followed by a 60 min reaction time. A final reaction volume of 20 µL was quenched with the addition of 43 µL of 0.6% (w/v) trifluoroacetic acid solution (Sigma, catalog #302031). Full-length human PRMT6 (1-375) was expressed in E. coli and purified. The PRMT6 assay was performed by dispensing 6 µL/well (final concentration of 10 nM) of PRMT6 protein diluted in assay buffer to the plate. Stock solutions of the test compounds were prepared in 100% DMSO (Sigma, Catalog #D2650) and serially diluted 1:3 using 100% DMSO. Compounds were additionally diluted 1:50 in assay buffer, and 6 µL/well was transferred to the assay plate. Plates were allowed to incubate for 15 min at room temperature. 12 µL/well of SAM (Cayman Chemical, Catalog #13956) and Histone H4 (36-50) K44 Me1 (Rockland, catalog, #000-001-K44) (final concentrations of 3 µM and 250 nM respectively) diluted in assay buffer, were added to the plate followed by a 30 min reaction time. A final reaction volume of 20 µL was quenched with the addition of 43 µL of 0.6% (w/v) trifluoroacetic acid solution (Sigma, catalog #302031). PRMT1, 4, and 6 assay plates were transferred to the RapidFire 365 autosampler coupled to an Agilent QQQ 6460 mass spectrometer. RapidFire buffer A contained water and buffer B was 80% acetonitrile/water. The samples were loaded onto a C18 type C (Agilent, catalog #G9203-80105) cartridge with load/wash time = 3000 ms, elute time = 4000 ms and re-equilibrate time = 500 ms. The flow rates for pumps 1, 2, and 3 are as follows: 1.5, 1.25, and 1.25 mL/min respectively. SAH peaks were integrated using RapidFire peak software and IC50 values were calculated using a four-parameter logistic curve fit using Genedata Screener software.

### Statistics and reproducibility

Statistical analyses were performed using Microsoft Excel or GraphPad Prism 8. For two-group comparisons, statistical significance was determined by a two-tailed Student’s *t*-test. For three or more group comparisons, statistical significance was determined by 1-way ANOVA. When two independent variables were considered, 2-way ANOVA was used. For in vivo studies, statistical significance was calculated by repeated-measures two-way ANOVA with multiple comparisons and Tukey correction, or Sidak correction as indicated. Plotted values are represented as the mean + /− standard deviation (SD) or standard error of the mean (SEM) as indicated. The exact *n* value and entity is reported in figure legends. Experiments included independent samples and were independently repeated at least two times or as stated in the text with consistent results.

### Reporting summary

Further information on research design is available in the Nature Research Reporting Summary linked to this article.

## Supplementary information

Supplementary Information

Description of Additional Supplementary Files

Supplementary Data 1

Supplementary Data 2

Supplementary Data 3

Supplementary Data 4

Supplementary Data 5

Supplementary Data 6

Supplementary Data 7

Reporting Summary

## Data Availability

The authors declare that the data supporting the findings of this study are available within the paper and its supplementary information files. Source data for Figures and Supplementary figures are provided as a Source Data file. All high-throughput sequencing datasets (RNA-seq, DRIP-seq) were deposited on the NCBI GEO website with accession code GSE130242. Source data are provided with this paper.

## Source data

Source Data
