# Supplementary material for: PRMT1-dependent regulation of RNA metabolism and DNA damage response sustains pancreatic ductal adenocarcinoma
Source: Nat Commun. 2021 Jul 30;12:4626. doi: 10.1038/s41467-021-24798-y (PMC8324870; doi:10.1038/s41467-021-24798-y)
Supplement: Supplementary file 1 — Supplementary Information [file 41467_2021_24798_MOESM1_ESM.pdf]

# Supplementary Figure 1

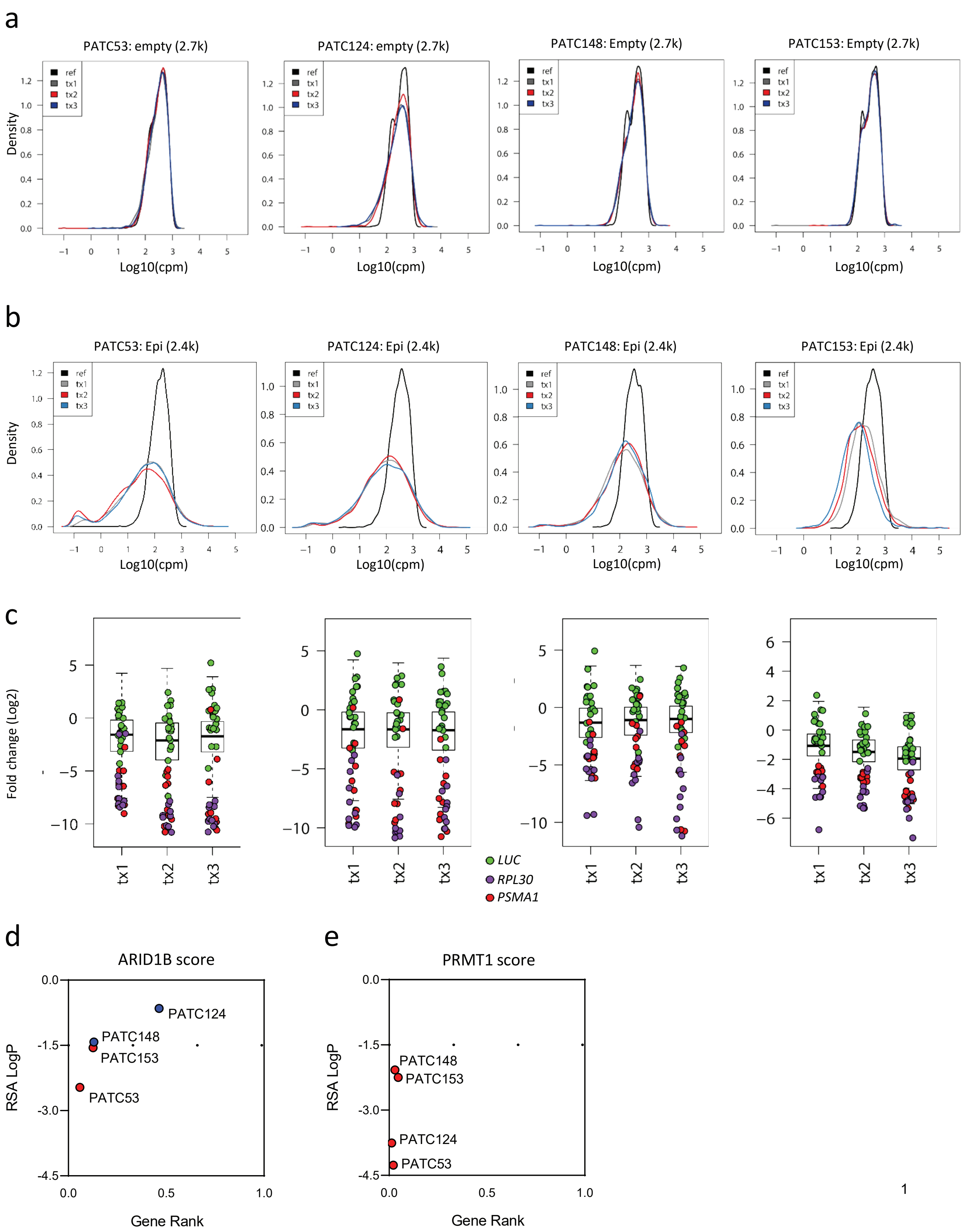

**Supplementary Figure 1. Epigenome screens in patient-derived xenografts (PDX) models of PDAC.**
(a) Complexity assessment in PDAC PDX-derived models (PATC53, PATC124, PATC148 and PATC153) using a non-targeting molecular barcoded lentiviral library (2.7k). Density plots of detected barcodes (Log10 cpm) were generated comparing reference cells pre-injection (ref) with 3 independent PDX tumors (tx1, tx2, tx3). Barcode distribution across samples largely overlaps within each PDX-derived model confirming *in vivo* complexity coverage of this library. (b) Density plots of barcode (shRNA) distribution for PDX transduced reference cells (ref) and three *in vivo* tumor replicates (tx1, tx2, tx3) from four PDAC PDXs infected with the Epigenome shRNA lentiviral library. Reference cells pre-injection showed a very narrow Gaussian distribution while PDX tumors displayed a wide range of barcode distribution, in line with shRNA activity over time. (c) Fold change (Log2) distributions of positive (*PMSA1*, proteasome subunit alpha type-1, and *RPL30*, ribosomal protein L30) and negative (*Luciferase*) controls of PDAC *in vivo* functional genomic screens using the Epigenome shRNA library in all four PDX-derived cell lines. *PSMA1* (red dots) and *RPL30* (purple dots) are covered by 10 shRNAs each. *Luciferase* (green dots) is covered by 20 shRNAs. Fold-change separation of positive and negative controls serves as a performance metric for screen quality. Box-and-whisker plots in these panels depict 25%–75% in the box, whiskers are at the 95 and 5 percentiles, and median is indicated with a line in the middle of the box. (d-e) Gene rank analysis highlighting the behavior of *ARID1B* and *PRMT1* genes in the Epigenome *in vivo* screens that used four PDAC PDX-derived models (shRNA dropout score = RSA logP). ARID1B knock-down showed higher sensitivity in ARID1A mutated tumors than ARID1A wild-type (d; Red = ARID1A mutant, Blue = ARID1A wild-type). PRMT1 knock-down showed to be a top-scoring gene across all the PDAC models (e). RSA LogP value of -1.5 is highlighted by the dotted line.

## Supplementary Figure 2

a

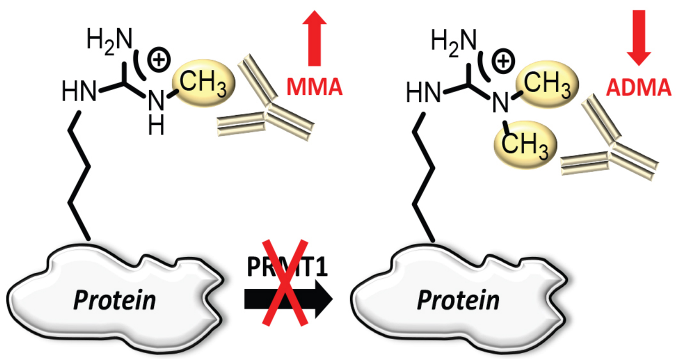


b

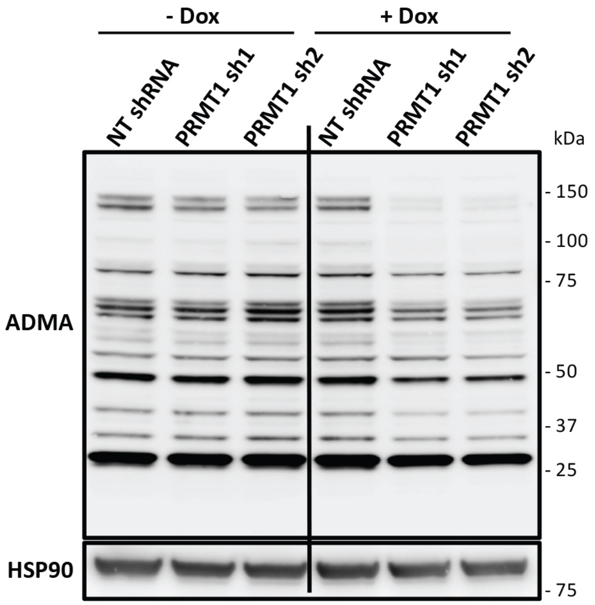


c

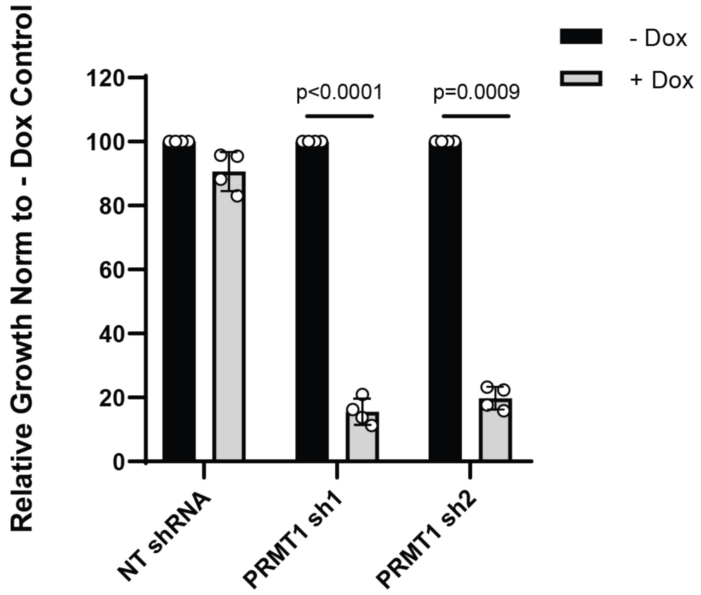

**Supplementary Figure 2. PRMT1 depletion decreases global asymmetric arginine methylation (ADMA) and increases arginine monomethylation (MMA).**

(a) Graphical representation of the effect of PRMT1 depletion on global arginine methylation status. Methylarginine-specific antibodies are used to monitor PRMT1-dependent arginine methylation. (b) ADMA levels in PATC53 cells engineered with doxycycline (DOX)-inducible *PRMT1*-targeting or non-targeting (NT, control) shRNAs. Cells were treated with or without 0.5 µg/mL DOX for 72h and then cell lysates were collected for Western Blot analysis. (c) Quantification of colony formation assays relative to Figure 1e. Data are presented as the mean +/- S.D. and *p* values are calculated by 1-way ANOVA compared to –dox control (n=4 biologically independent samples).

# Supplementary Figure 3

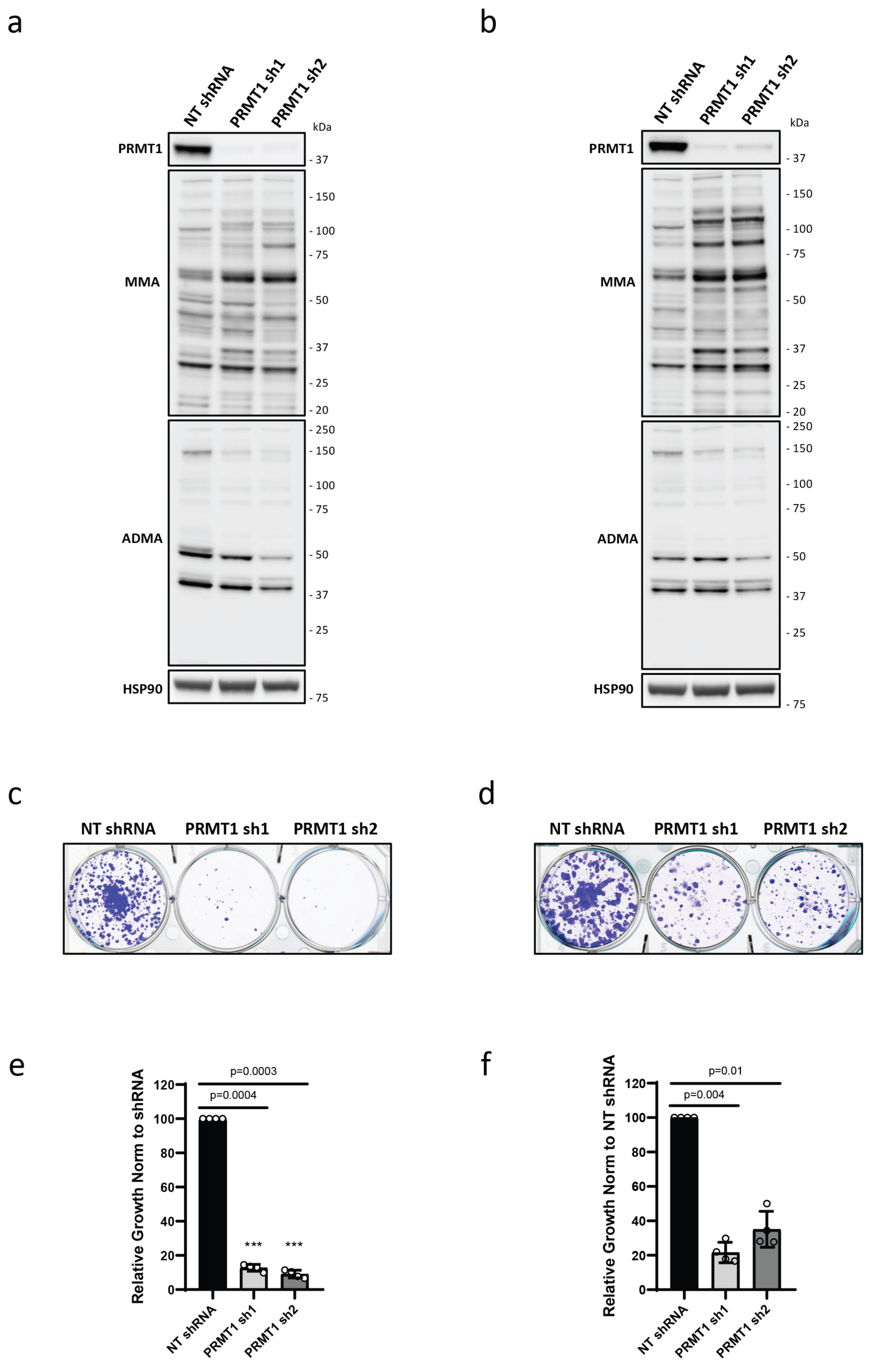

**Supplementary Figure 3. PRMT1 genetic knock-down impairs cell growth of patient-derived cells.**
(a-b) PRMT1 genetic knock-down in PATC153 (a) and PATC148 (b) cells engineered with two independent *PRMT1*-targeting or a non-targeting (NT) shRNA. After puromycin selection, cell lysates were collected to evaluate PRMT1 expression and global methylation status. HSP90 is shown as representative loading control. (c-d) Representative crystal violet staining image of colony formation assays of PATC153 (c) and PATC148 (d) cells engineered as described in (a-b). (e-f) Quantification of colony formation assays of PATC153 (e) and PATC148 (f) cells engineered as described in (a-b). Data are presented as the mean +/- S.D. and *p* values are calculated by 1-way ANOVA compared to NT shRNA controls (n=4 biologically independent samples).

# Supplementary Figure 4

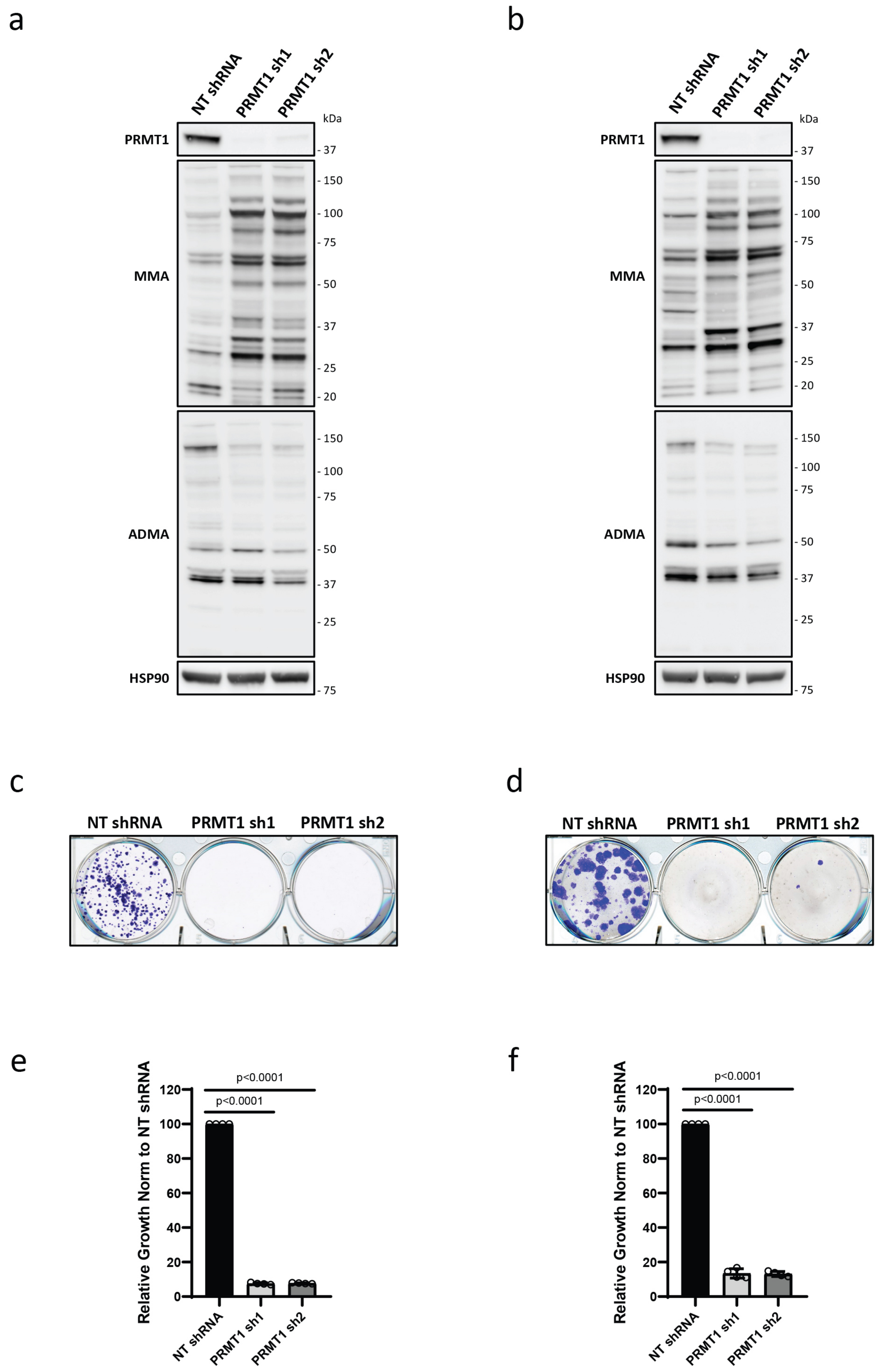

**Supplementary Figure 4. PRMT1 genetic knock-down impairs cell growth of PDAC cell lines.**
(a-b) PRMT1 genetic knock-down in PANC1 (a) and PK59 (b) cells engineered with two independent *PRMT1*-targeting or a non-targeting (NT) shRNA. After puromycin selection, cell lysates were collected to evaluate PRMT1 expression and global methylation status. HSP90 is shown as representative loading control. (c-d) Representative crystal violet staining image of colony formation assays of PANC1 (c) and PK59 (d) cells engineered as described in (a-b). (e-f) Quantification of colony formation assays of PANC1 (e) and PK59 (f) cells engineered as described in (a-b). Data are presented as the mean +/- S.D. and *p* values are calculated by 1-way ANOVA compared to NT shRNA controls (n=4 biologically independent samples).

## Supplementary Figure 5

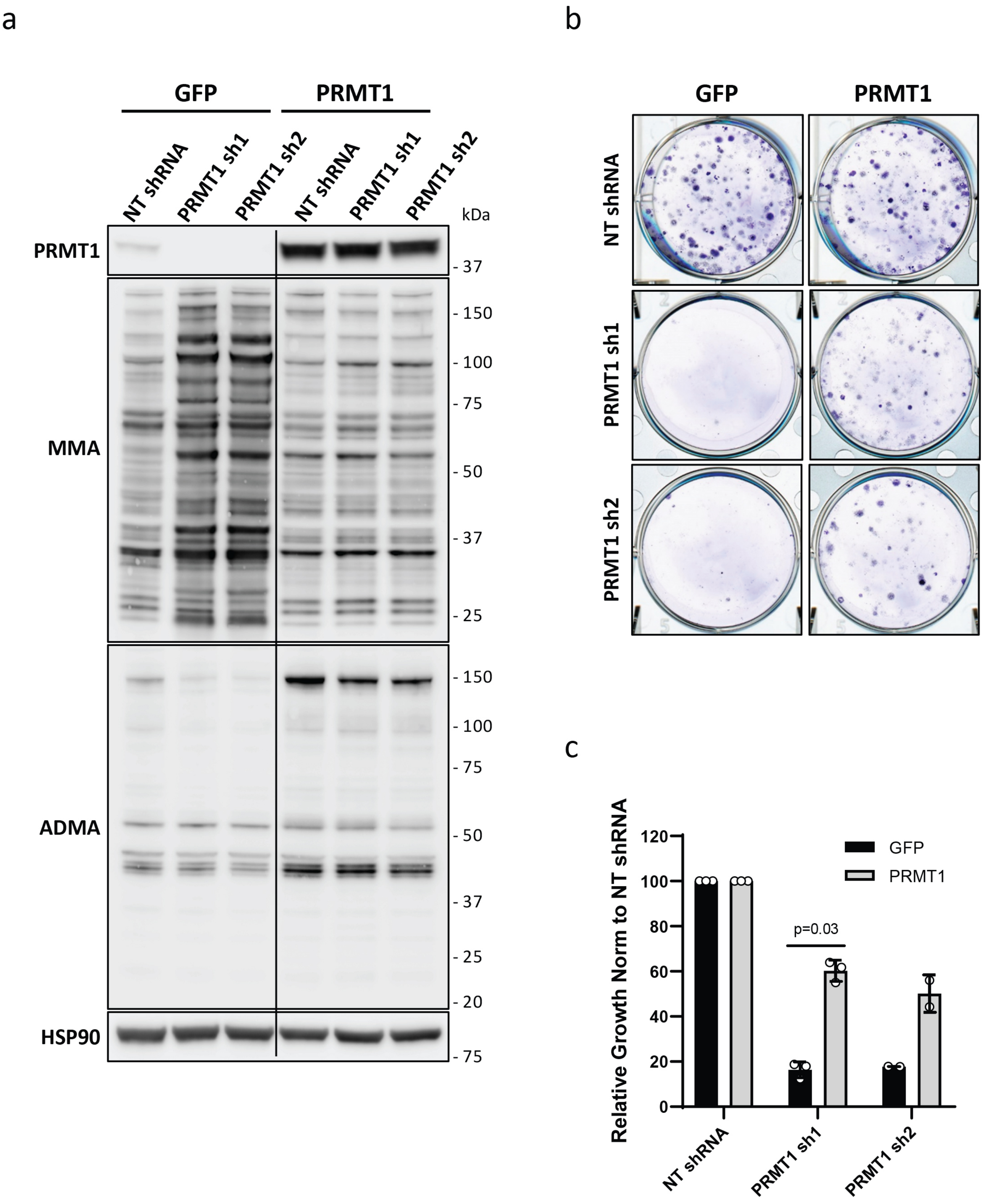

**Supplementary Figure 5. Ectopic expression of PRMT1 restores global methylation status and cell growth.**

PATC53 cells were engineered with pLenti-*GFP* or pLenti-*PRMT1* shRNA-resistant cDNAs and shRNAs targeting endogenous *PRMT1* or a non-targeting (NT) control. (a) Western Blot analysis of PRMT1 expression and global arginine methylation status. HSP90 is shown as representative loading control. (b) Colony formation assay. Representative crystal violet staining image of engineered cells. (c) Quantification of colony formation assays normalized to NT controls. Data are presented as the mean +/- S.D. and *p* values are calculated by two-tailed Student's t-test (n=3 biologically independent samples for *PRMT1* sh1; n=2 biologically independent samples for *PRMT1* sh2).

# Supplementary Figure 6

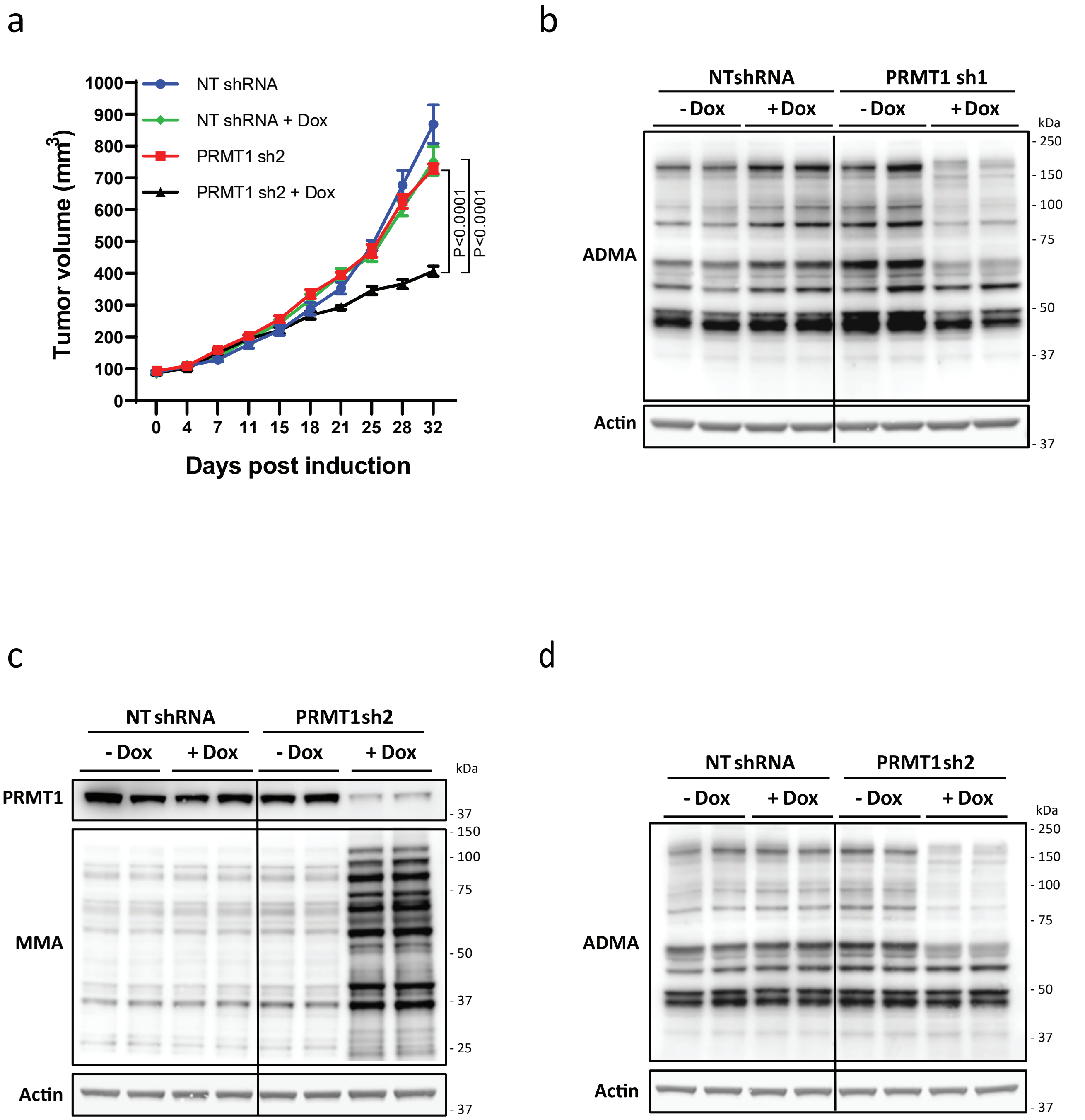

**Supplementary Figure 6. PRMT1 genetic knock-down inhibits tumor growth.**
(a) Tumor growth curve ($mm^3$) of PATC53 xenografts harboring an independent from Fig. 1f doxycycline (DOX)-inducible *PRMT1*-targeting (sh2) or non-targeting (NT) control (n=8 mice/group). Data are presented as the mean +/- SEM and *p* values are calculated by 2-way ANOVA with multiple comparisons and Tukey's correction. This *in vivo* study is an independent study from Figure 1f. (b) Western Blot analysis of ADMA levels from PATC53 tumor lysates described in Fig. 1h. (c and d) Western Blot analysis of PRMT1 and MMA (c) or ADMA (d) levels in PATC53 tumor lysates harboring DOX-inducible *PRMT1*-targeting (sh2) or non-targeting (NT) shRNA 10 days post DOX induction.

# Supplementary Figure 7

a

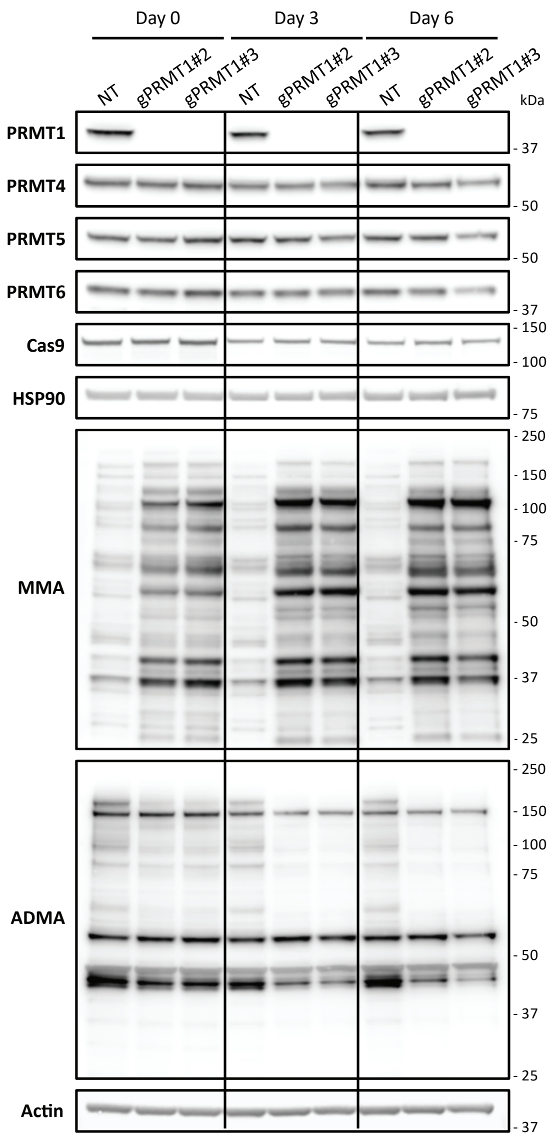


b

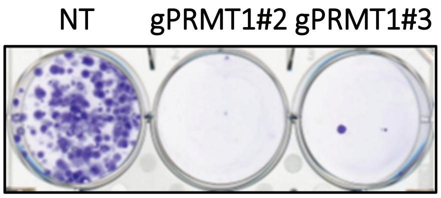


c

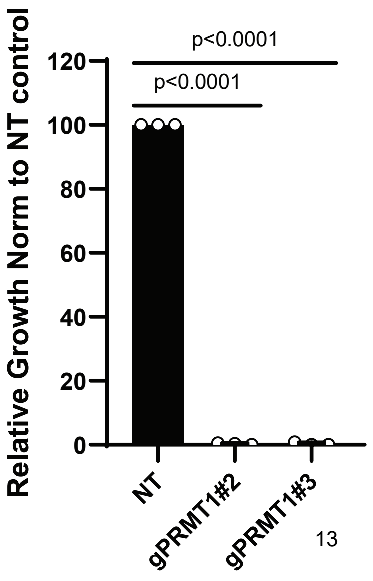

**Supplementary Figure 7. CRISPR/Cas9-mediated PRMT1 knock-down modulates arginine methylation status and impairs cell growth.**

(a) Western blot analysis of the expression of PRMT1, PRMT4, PRMT5 and PRMT6 as well as global arginine methylation status in PATC53 cells engineered with two independent guides RNA targeting *PRMT1*, or with a non-targeting (NT) control. Cell lysates were collected right after selection (Day 0) as well as 3 (Day 3) and 6 (Day 6) days post-selection. (b) Colony formation assay. Representative crystal violet staining image of PATC53 cells engineered as described in (a), plated 3 days post-selection. (c) Quantification of colony formation assays. Data are presented as the mean +/- S.D. and *p* values are calculated by 1-way ANOVA compared to NT control (n=3 biologically independent samples).

# Supplementary Figure 8

a

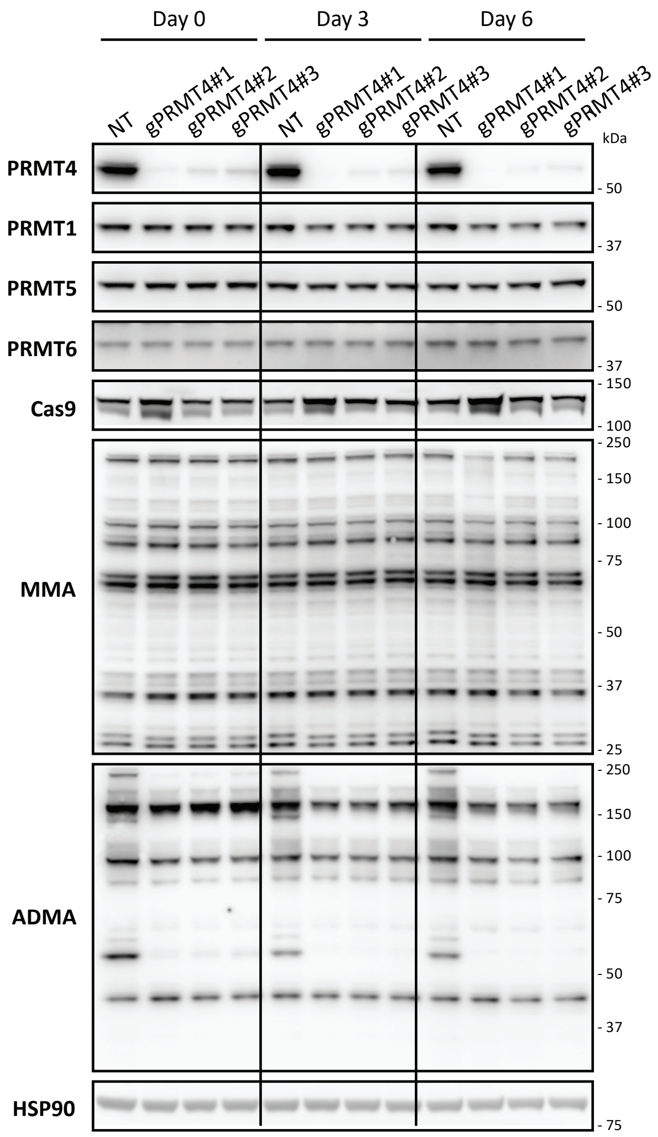


b

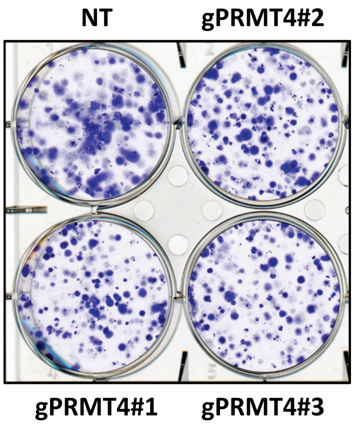


c

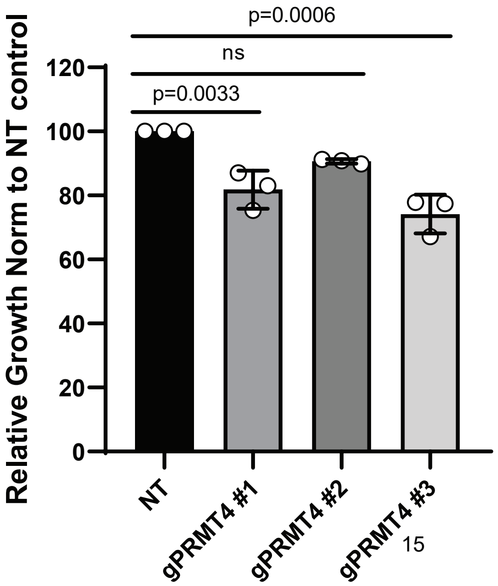

**Supplementary Figure 8. CRISPR/Cas9-mediated PRMT4 knock-down does not induce accumulation of MMA and modestly inhibits cell growth.**

(a) Western blot analysis of expression of PRMT4, PRMT1, PRMT5, PRMT6, as well as global arginine methylation status in PATC53 cells engineered with three independent guides RNA targeting *PRMT4* or with a non-targeting (NT) control. Cell lysates were collected right after selection (Day 0) as well as 3 (Day 3) and 6 (Day 6) days post-selection. (b) Colony formation assay. Representative crystal violet staining image of PATC53 cells engineered as described in (a), plated 6 days post-selection to ensure complete PRMT4 knock-down. (c) Quantification of colony formation assays. Data are presented as the mean +/- S.D. and *p* values are calculated by 1-way ANOVA compared to NT control (n=3 biologically independent samples).

# Supplementary Figure 9

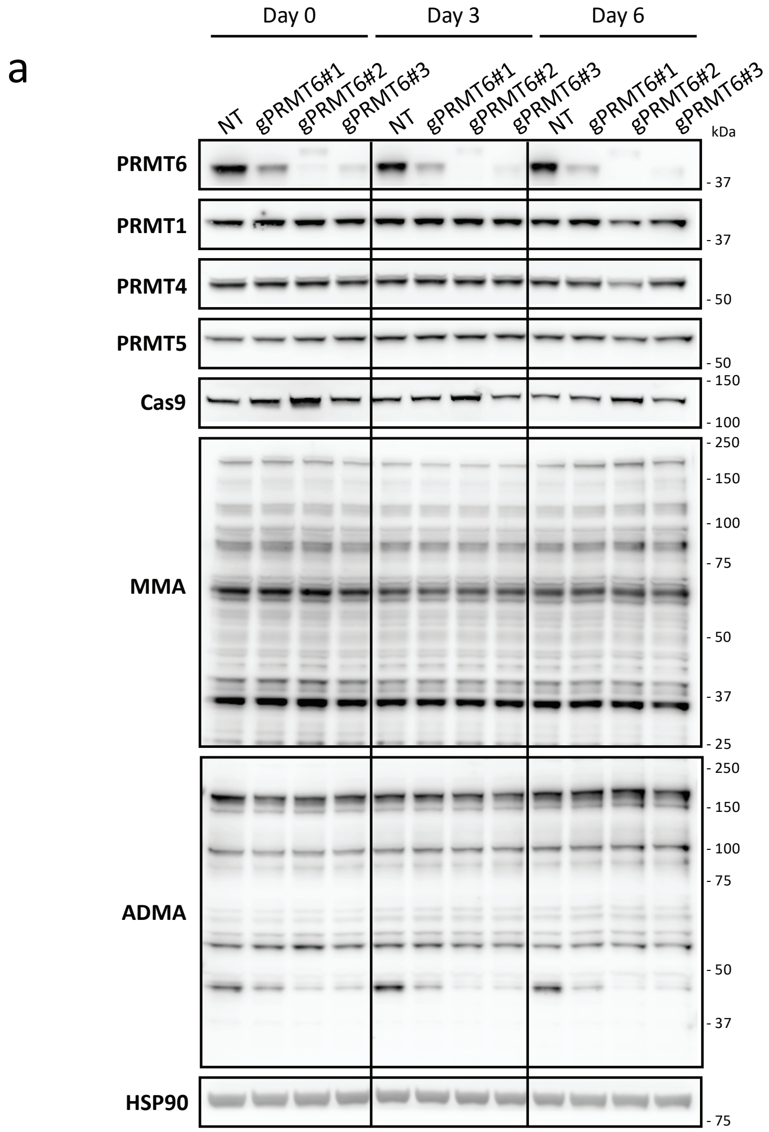


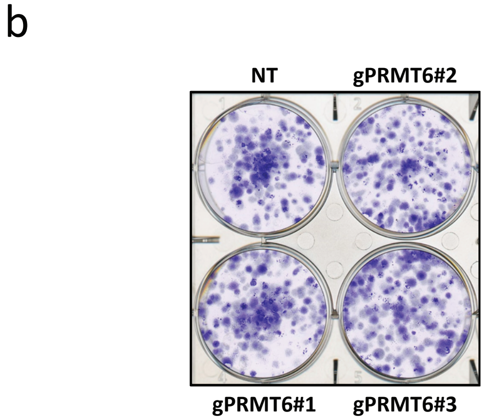


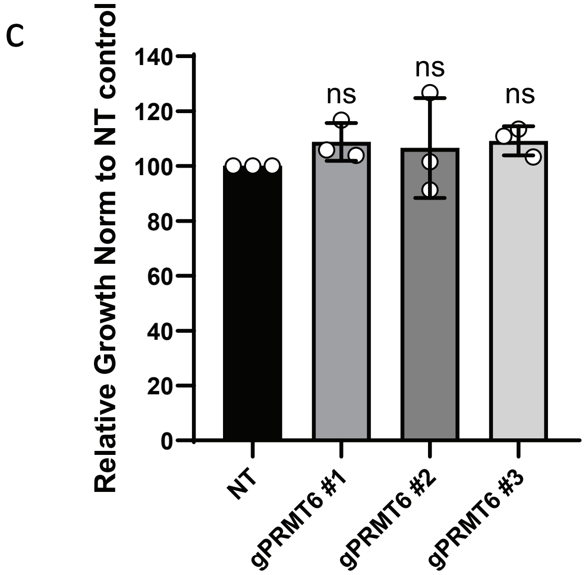


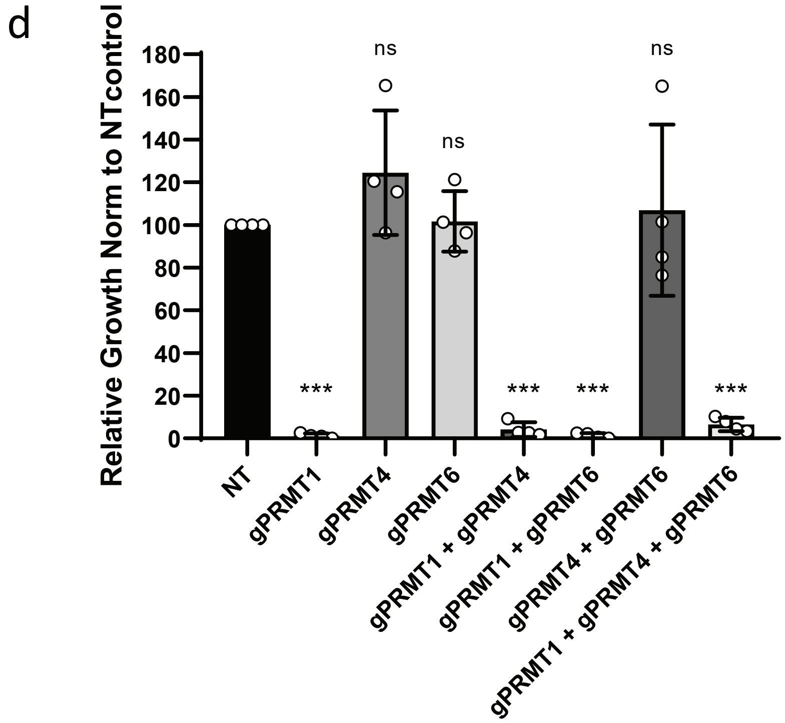

**Supplementary Figure 9. CRISPR/Cas9-mediated PRMT6 knock-down does not induce accumulation of MMA nor inhibits cell growth, consistent with PRMTs knock-down combination studies.**

(a) Western blot analysis of expression of PRMT6, PRMT1, PRMT4, PRMT5, as well as global arginine methylation status in PATC53 cells engineered with three independent guides RNA targeting *PRMT6*, or with a non-targeting (NT) control. Cell lysates were collected right after selection (Day 0) as well as 3 (Day 3) and 6 (Day 6) days post-selection. (b) Colony formation assay. Representative crystal violet staining image of PATC53 cells engineered as described in (a), plated 6 days post selection to ensure the complete PRMT6 knock-down. (c) Quantification of colony formation assays. Data are presented as the mean +/- S.D. and *p* values are calculated by 1-way ANOVA compared to NT control (ns=non-significant; n=3 biologically independent samples). (d) Quantification of colony formation assay relative to Figure 1k. Data are presented as the mean +/- S.D. and *p* values are calculated by 1-way ANOVA compared to NT control. For indicated significant differences (***), exact *p* values are reported from left to right bars: *p*= 0.0005; *p*= 0.0007; *p*= 0.0005; *p*=0.0008; ns= non-significant; n=4 biologically independent samples.

# Supplementary Figure 10

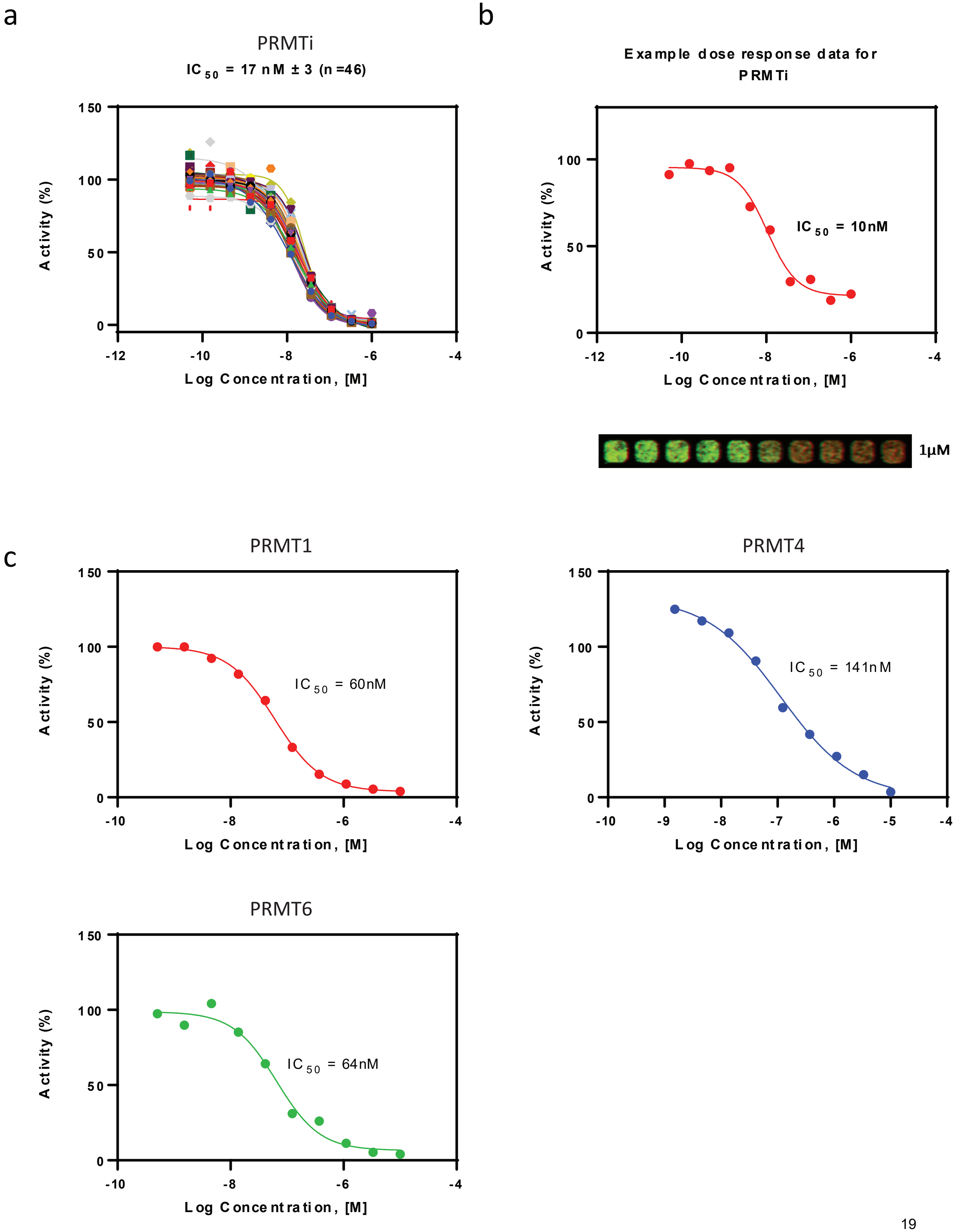

**Supplementary Figure 10: PRMTi profiling**

(a) PRMT1 enzymatic activity was measured using the LANCE TR-FRET assay from PerkinElmer. This assay follows the methylation of histone H4 at Arg3 using S-adenosyl-L-methionine (SAM) as the methyl group donor. The europium fluorescence signal and the ULight TR-FRET signal were measured: excitation at 330nm, emissions at 620nm and 665nm respectively, and the ratio of the two signals (665nm/620nm) were converted to percent of control. The values were then plotted against the log concentration of the compound. The different lines represent the individual dose response for all independent runs (n=46). (b) In-Cell Western assay measuring ADMA (asymmetric dimethyl arginase) used to assess PRMT1 cellular target engagement. ADMA levels were normalized with CellTag700TM (LiCor) and converted to percent of control. Values were plotted against the log concentration of the compound. A representative curve is shown (top). In-Cell Western using two-color detection of ADMA (green) normalized to CellTag700TM (red) in RKO cells is shown. Representative image shows a dose response curve of a 3-fold dilution of PRMTi starting at 1µM -> 50pM (bottom). (c) Selectivity data. Protein arginine methyltransferases (PRMTs) catalyze the transfer of the methyl group from the cofactor S-5′-adenosyl-L-methionine (SAM) to arginine residues of a variety of histone and nonhistone proteins. The production of S-(5′-Adenosyl)-L-homocysteine (SAH) was measured using Agilent's RapidFire 365-Agilent QQQ 6460 to assess selectivity between PRMT1, 4, and 6. SAM concentration was 1µM. PRMTi is a pan Type I inhibitor.

# Supplementary Figure 11

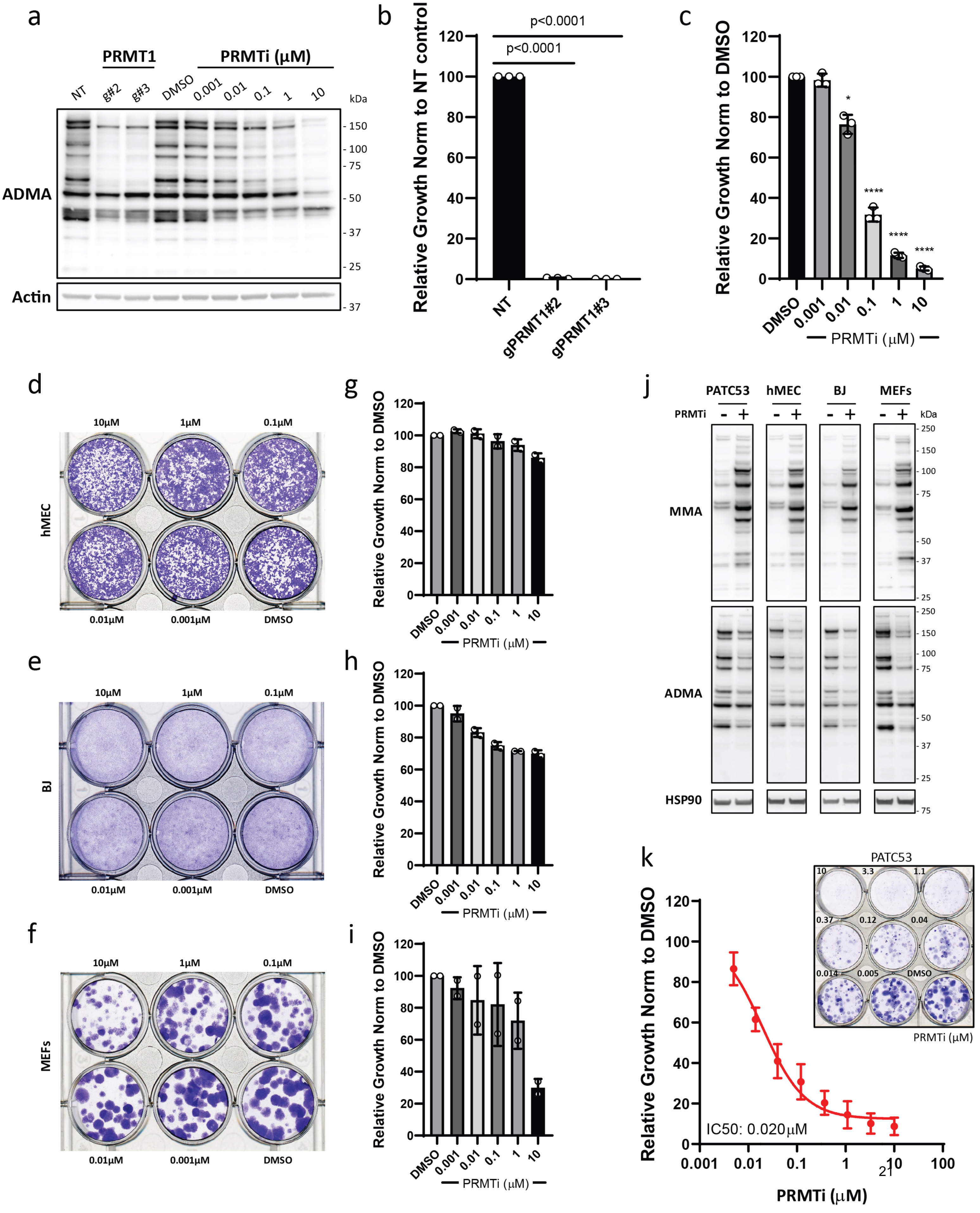

**Supplementary Figure 11. Inhibition of PRMT1 catalytic activity phenocopies genetic depletion in patient-derived cells.**

(a) Western Blot analysis of global ADMA in PATC53 cells either engineered with two independent guide RNA targeting *PRMT1* or with a non-targeting (NT) control, or treated with DMSO or PRMTi in a dose dependent manner for 48h. (b-c) Quantification of colony formation assays relative to Figure 2b. (b) Growth of PATC53 cells with or without CRISPR-Cas9-mediated knock-out of PRMT1. Data are presented as the mean +/- S.D. and *p* values are calculated by 1-way ANOVA compared to NT control (n=3 biologically independent samples). (c) PATC53 cells treated with indicated doses of PRMTi. Data are presented as the mean +/- S.D. and *p* values are calculated by 1-way ANOVA compared to DMSO treated cells. For indicated significant differences, exact *p* values are (*) $p$=0.0159; (****) $p$<0.0001; n=3 biologically independent samples. (d-i) Colony formation assays of normal diploid cells. (d-f) Representative crystal violet staining images of hMEC (d), BJ (e) and MEFs (f) cells treated with indicated concentrations of PRMTi. (g-i) Quantification of colony formation assays for hMEC (g), BJ (h) and MEFs (i). Data are presented as the mean +/- S.D. of n=2 independent experiments. (j) Western blot analysis of global MMA and ADMA across indicated cells treated with 1 µM PRMTi for 24h. (k) Representative dose response curve in colony formation assay of PATC53 cells treated with PRMTi for 14 days. Cellular growth is quantified measuring the absorption of crystal violet dye solubilized from stained cells. Data are presented as the mean +/- S.D., n=6 independent experiments. Representative images of crystal violet staining are also shown.

# Supplementary Figure 12

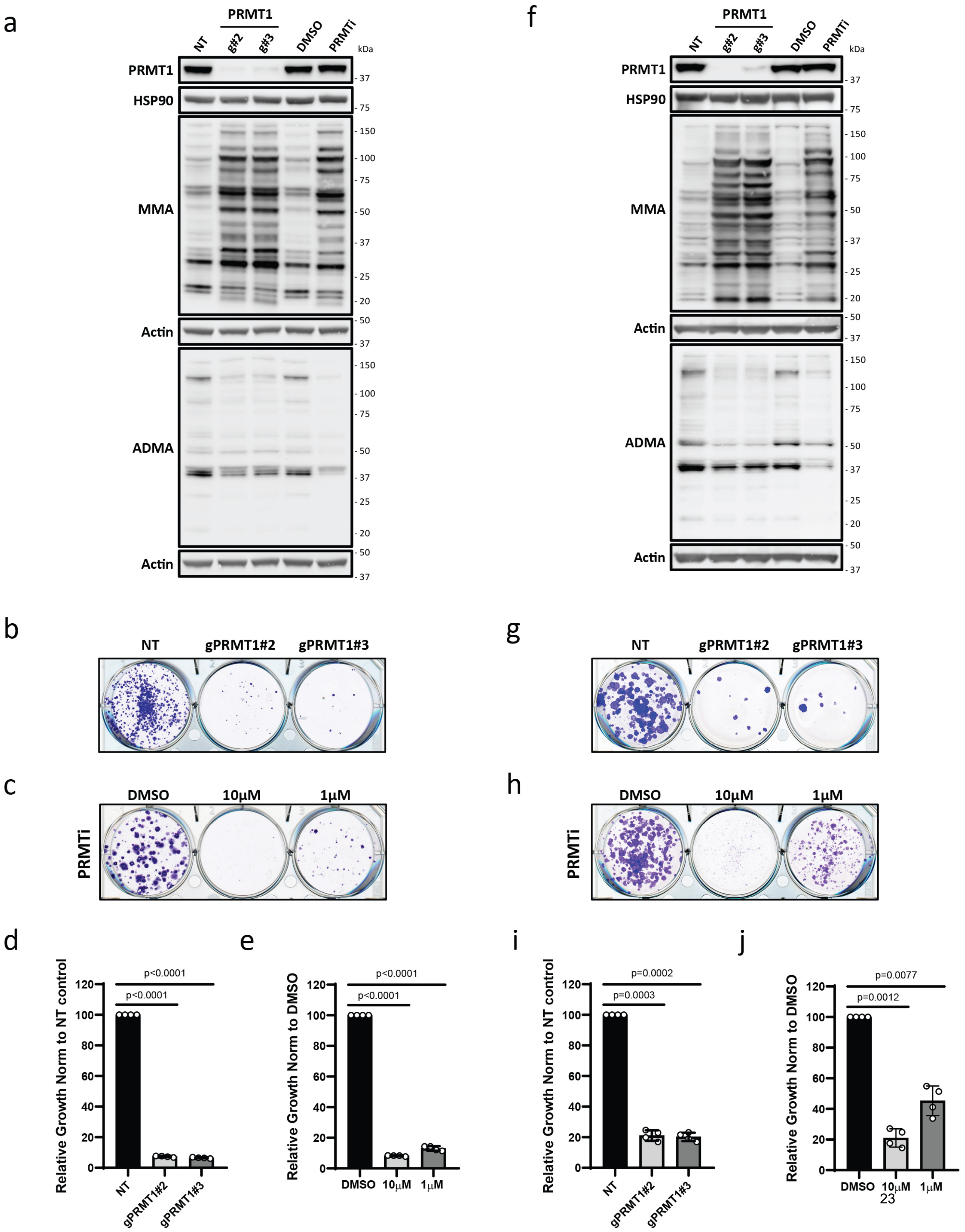

**Supplementary Figure 12. Inhibition of PRMT1 catalytic activity phenocopies genetic depletion in PDAC cell lines.**

(a) Western Blot analysis of PRMT1 expression and global MMA and ADMA in PANC1 cells either engineered with two independent guides RNA targeting *PRMT1* or with a non-targeting (NT) control, or treated with DMSO control or PRMTi at 1 µM for 48h. (b-e) Colony formation assays. Representative crystal violet staining image and colony assays quantification of PANC1 cells upon PRMT1 CRISPR-Cas9-mediated knock-down (b and d, respectively) or treated with PRMTi at 10 µM or 1 µM for 14 days (c and e, respectively). Data are presented as the mean +/- S.D. and *p* values are calculated by 1-way ANOVA compared to respective control (n=4 biologically independent samples). (f) Western Blot analysis of PRMT1 expression and global MMA and ADMA in PK59 cells either engineered with two independent guides RNA targeting *PRMT1* or with a non-targeting (NT) control, or treated with PRMTi at 1 µM for 48h. (g-j) Colony formation assays. Representative crystal violet staining image and colony assays quantification of PK59 cells upon PRMT1 CRISPR-Cas9-mediated knock-down (g and i, respectively) or treated with PRMTi at 10 µM or 1 µM for 14 days (h and j, respectively). Data are presented as the mean +/- S.D. and *p* values are calculated by 1-way ANOVA compared to respective control (n=4 biologically independent samples).

# Supplementary Figure 13

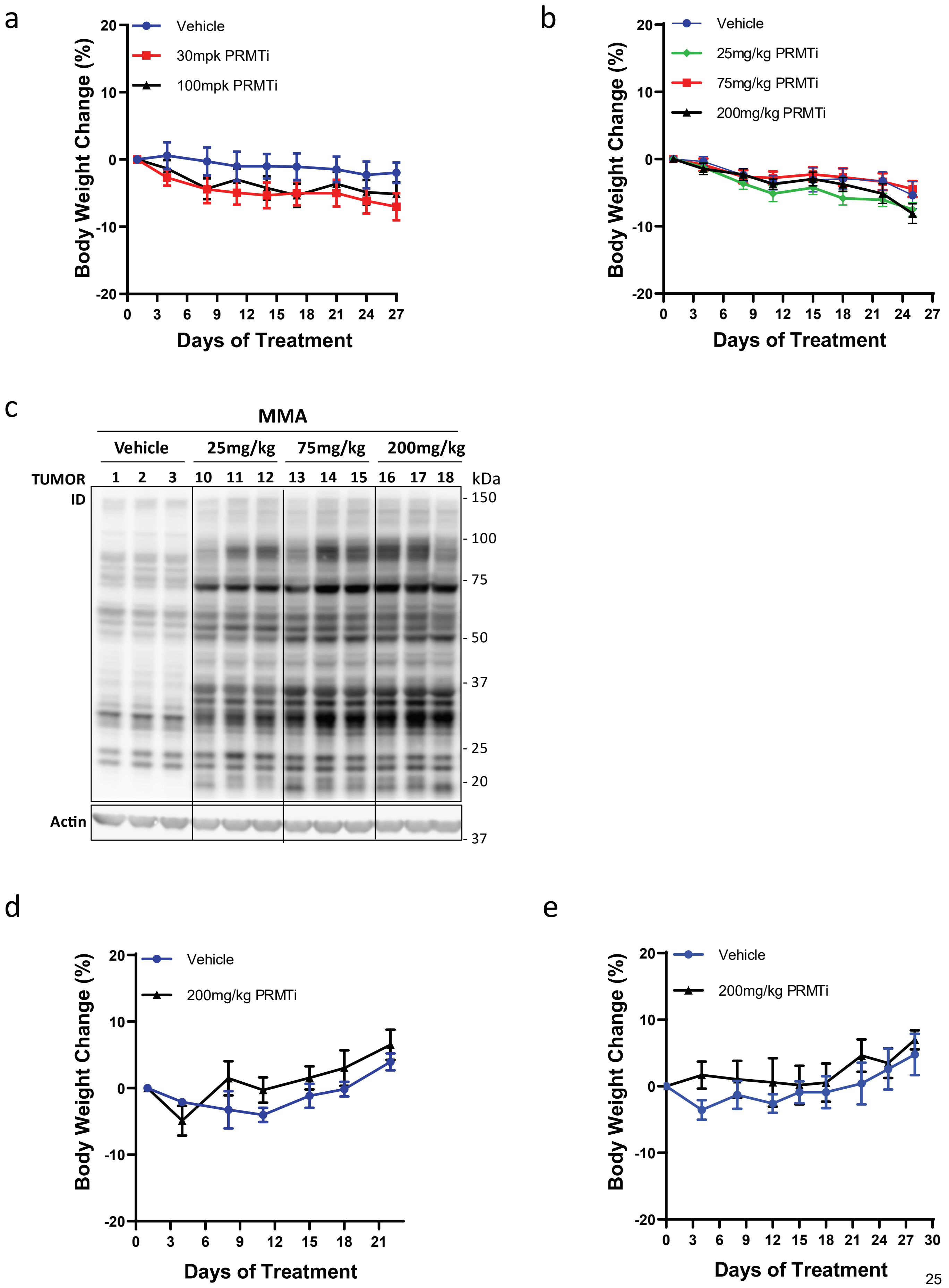

**Supplementary Figure 13**. **PRMT1 catalytic activity is required to maintain tumor growth in human PDAC models.**

(a) Percentage change in mean body weight (BW) from Day 1 for mice bearing PATC53 xenografts treated with PRMTi at 30 mg/kg and 100 mg/kg BID for 4 weeks. Data are presented as the mean +/- SEM, n=8-10 mice/group. (b) Percentage change in mean BW from Day 1 for mice bearing PANC1 xenografts treated with PRMTi at 25 mg/kg, 75 mg/kg, and 200 mg/kg once daily (QD) for 4 weeks. Data are presented as the mean +/- SEM, n=8 mice/group. (c) Western Blot analysis of MMA in PANC1 tumor lysates 4 days post PRMTi treatment. (d-e) Percentage change in mean BW from Day 1 for mice bearing PATX153 (d) or PATX60 (e) xenografts treated with PRMTi at 200 mg/kg QD, 5on/2off, for 3 weeks or 4 weeks, respectively. Data are presented as the mean +/- SEM, n=5 mice/group (d). For (e) n=4 mice/group vehicle; n=3 mice/group PRMTi.

# Supplementary Figure 14

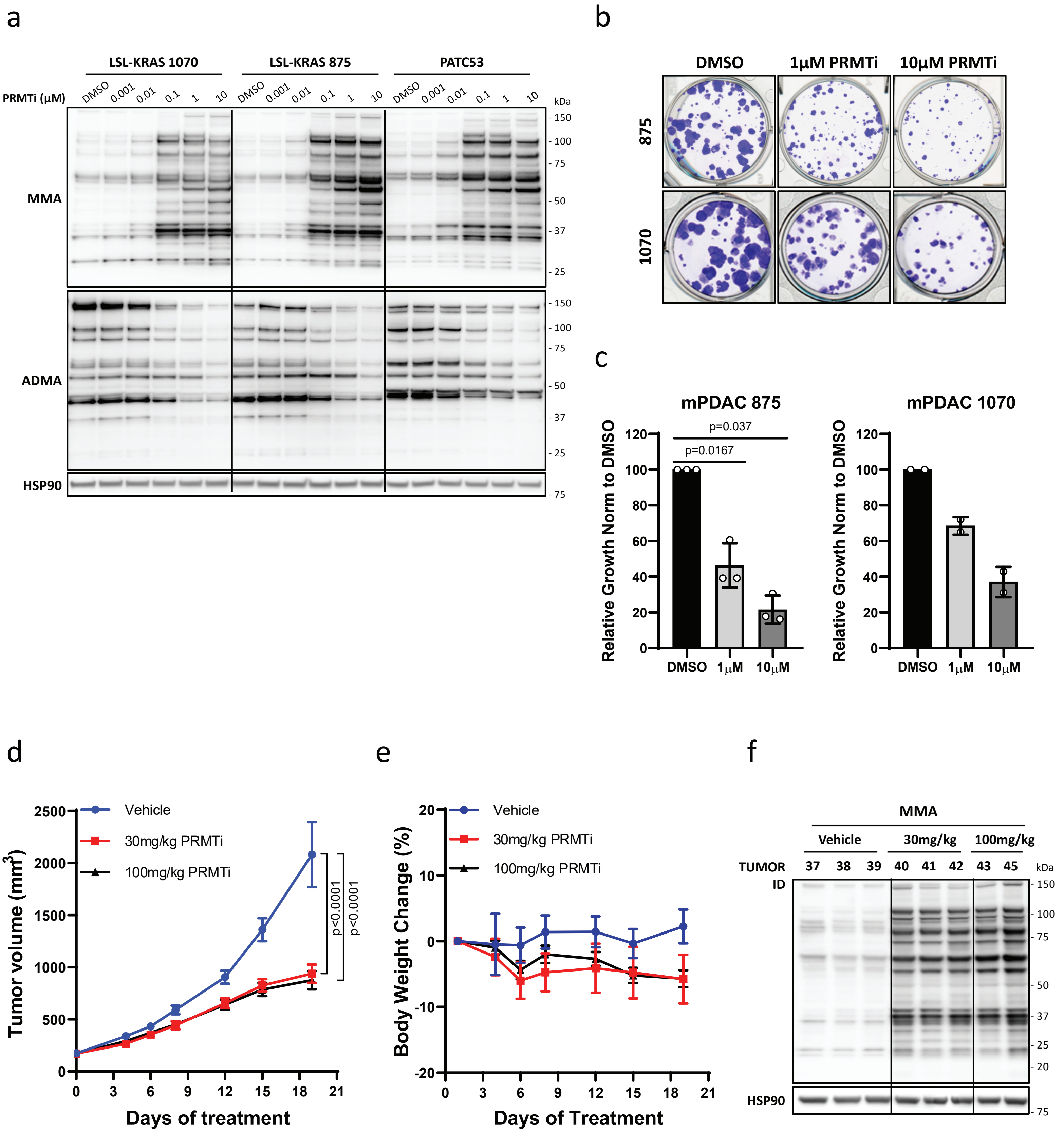

**Supplementary Figure 14**. **Prmt1 is required to maintain tumor growth in mouse PDAC models.**
(a) Western Blot analysis of global MMA and ADMA status in two mouse (875 and 1070) and one human (PATC53) KRAS/p53 mutant PDAC models treated with PRMTi at the indicated concentrations or with DMSO control for 24h. (b) Colony formation assay. Representative crystal violet staining images of mouse KRAS/p53 mutant PDAC models, 875 and 1070, treated with DMSO control or PRMTi at 1 µM and 10 µM for 14 days. (c) Quantification of colony formation assays in (b). Data are presented as the mean +/- S.D. and *p* values are calculated by 1-way ANOVA compared to DMSO control. 875, n=3 biologically independent samples; 1070, n=2 biologically independent samples. (d) Tumor growth curve ($mm^3$) of mouse KRAS/p53 mutant PDAC 875 allografts treated with PRMTi at 30 mg/kg and 100 mg/kg BID for 19 days. Data are presented as the mean +/- SEM and *p* values are calculated by 2-way ANOVA with multiple comparisons and Tukey's correction, compared to vehicle control (n=10 mice/group). (e) Percentage change in mean BW from Day 1 for mice described in (d). Data are presented as the mean +/- SEM, n=10 mice/group). (f) Western Blot analysis of MMA in 875 tumor lysates 7 days post PRMTi treatment.

# Supplementary Figure 15

a

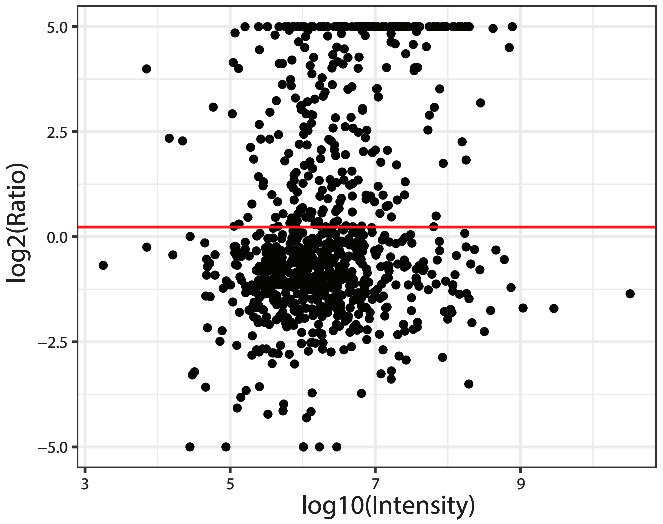


b

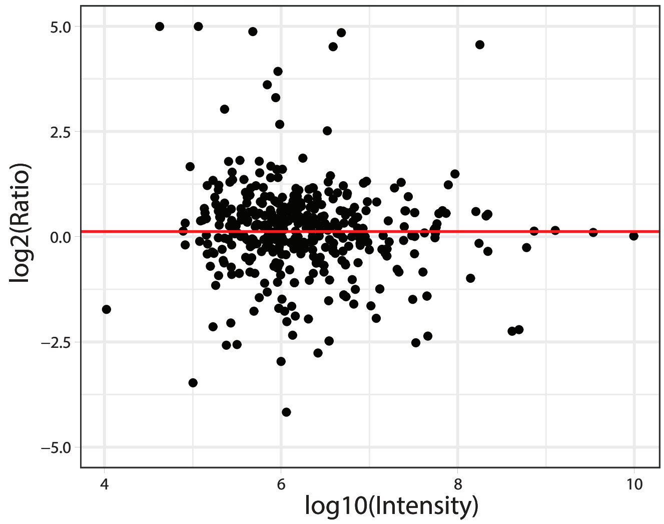


c

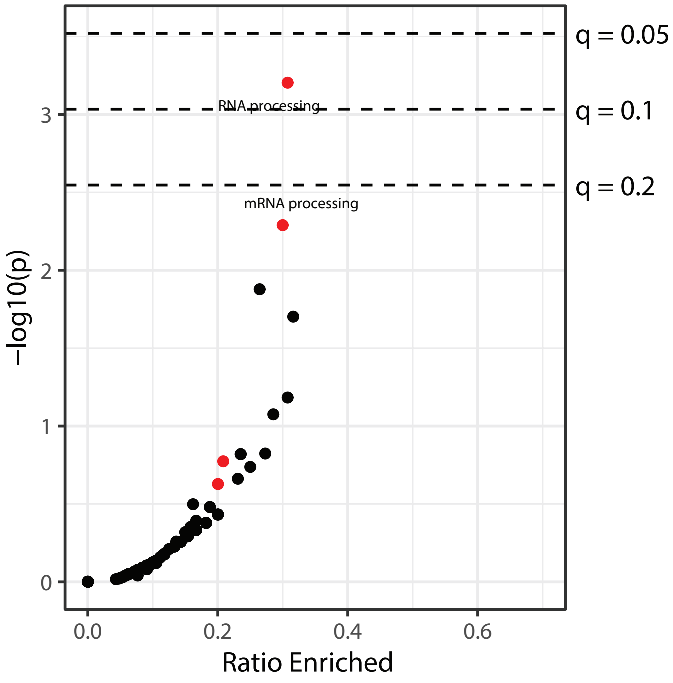

**Supplementary Figure 15. Identification of PRMT1 substrates and binding partners.**
(a-b) PATC53 cells were treated with 1 μM PRMTi or DMSO control for 24h and modified peptide were enriched by immunoaffinity purification with methylarginine-specific antibodies for MMA (a) or ADMA (b) and resolved by LC-MS/MS. MA plots of log2 fold change of peptide abundance between treated and control conditions against the log average intensity for each peptide. Average log2 fold change of all peptides denoted by a horizontal red line. (c) Enrichment analysis for gene ontology (GO) biological processes was conducted using a Fisher's exact test for proteins with ADMA with decreased intensity on treatment vs. all proteins analyzed. The x-axis indicates the ratio of enriched vs. total genes analyzed per GO term. Dashed lines indicate FDR-adjusted significance thresholds at $q = 0.2$, $q = 0.1$, and $q = 0.05$. RNA processing terms are most enriched, but no GO terms were enriched with $q < 0.05$.

# Supplementary Figure 16

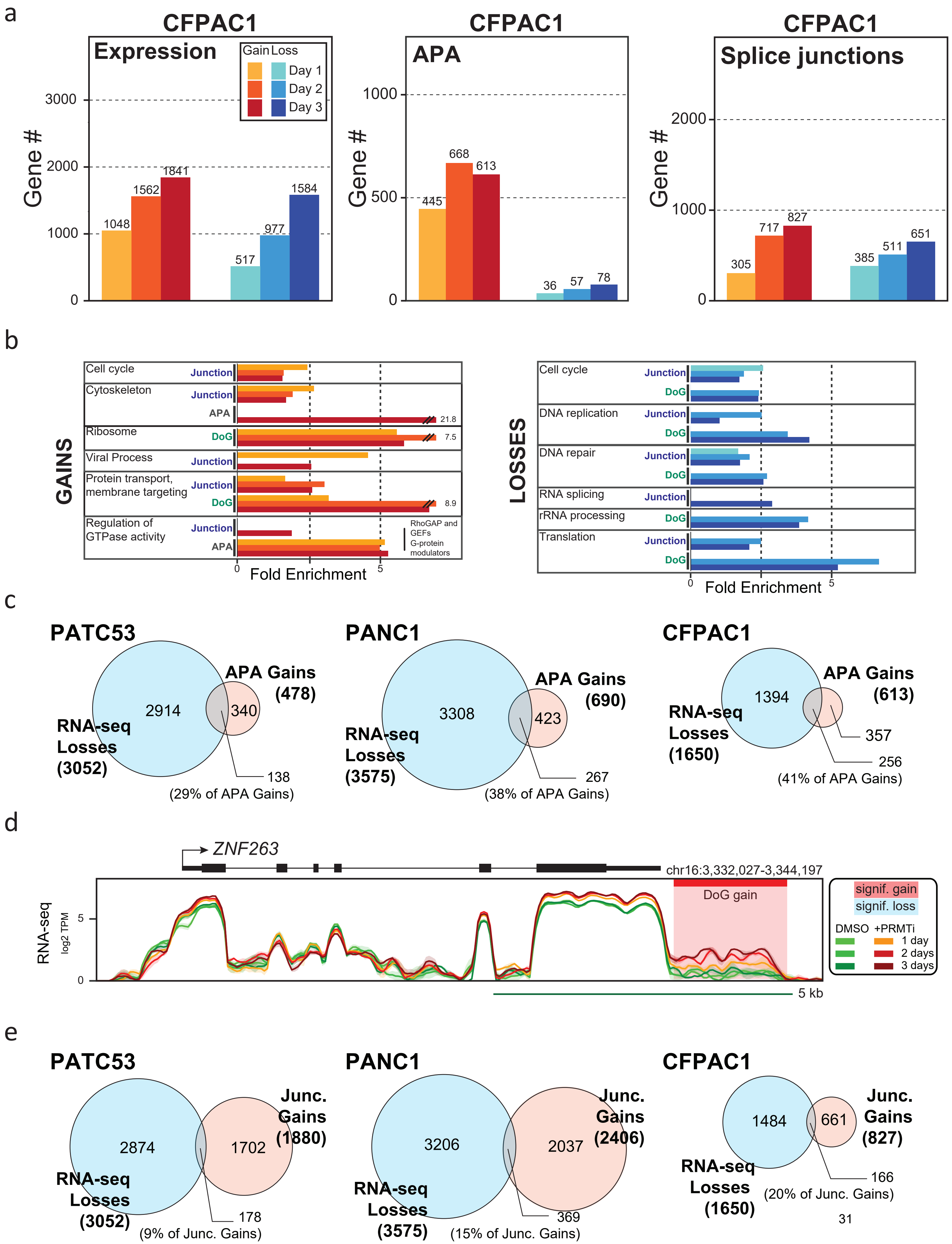

**Supplementary Figure 16. PRMT1 plays a critical role in regulating gene expression and co-transcriptional RNA processing.**

(a) Number of genes showing significant gain or loss of expression (left), alternative polyadenylation (APA) (middle), and splicing junctions (right) in CFPAC1 cells after 1, 2, and 3 days of 1 μM PRMTi. (b) Summary of significant GO ontologies (PANTHER) observed for genes undergoing significant gains (left) and losses (right) in co-transcriptional processing events. Changes in junctions, downstream of gene (DoG) transcription, and APA upon 1 μM PRMTi treatment are presented as a function of time in PATC53 and PANC1 cells (color code is as defined in panel (a)). (c) Venn diagrams show overlap between genes undergoing significant APA gain and gene expression loss for PATC53 (left), PANC1 (middle), and CFPAC1 (right) cell lines treated with 1 μM PRMTi for 3 days. (d) Representative screenshot of RNA-seq data over *ZNF263*, a gene that undergoes a gain of DoG transcription upon 1 μM PRMTi treatment in PANC1 cells, for the indicated time points. (e) Venn diagrams as described in (c) except for genes undergoing splice junction gains.

# Supplementary Figure 17

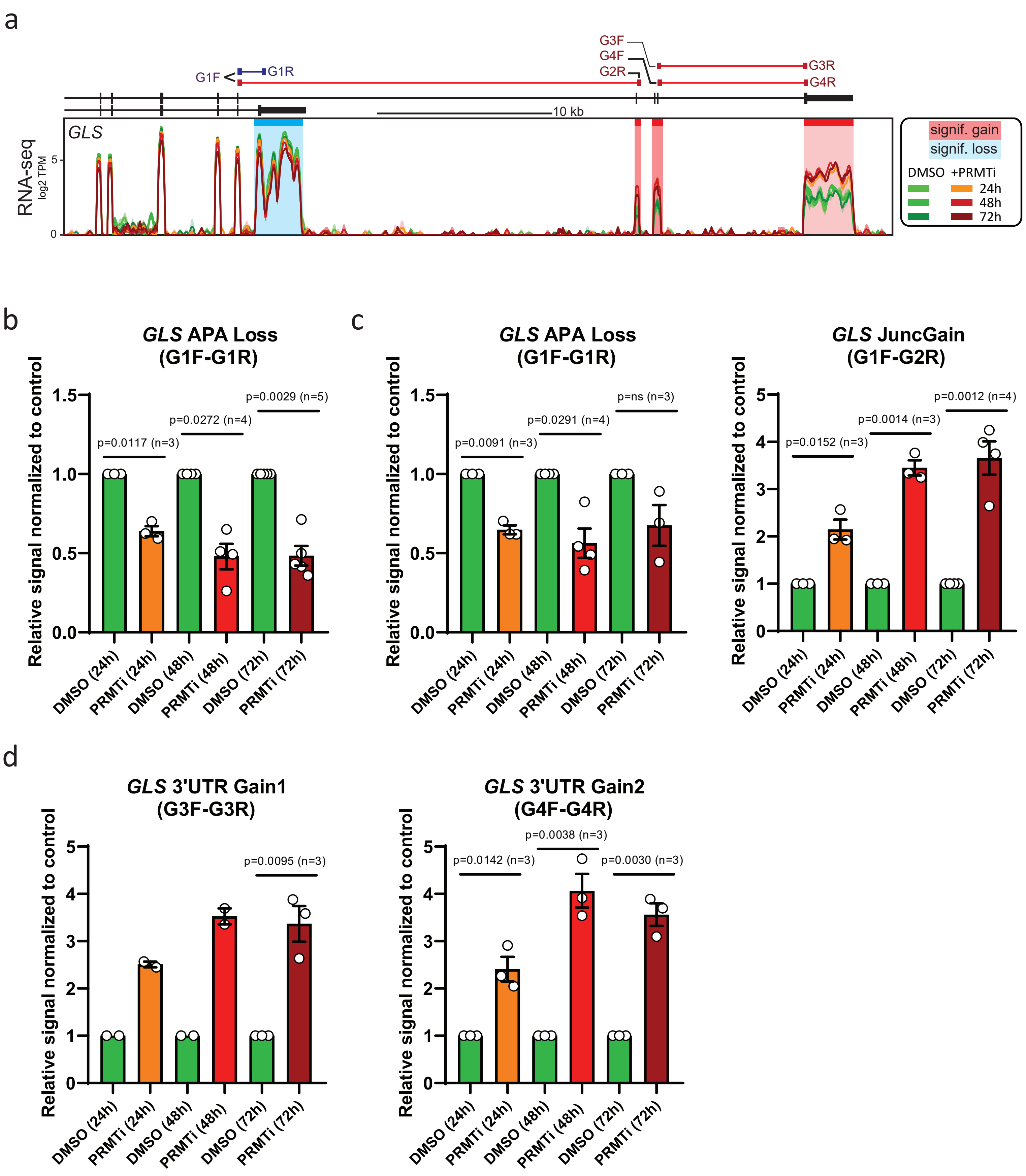

**Supplementary Figure 17. RT-qPCR validation of *GLS* alternative polyadenylation.**

(a) Schematic representation of RT-qPCR primers visualized over a composite screenshot of RNA-seq data over *GLS* gene in PANC1 cells. Data from treatment with DMSO or 1 µM PRMTi are color-coded as indicated. (b-d) RT-qPCR validation of alternative polyadenylation in *GLS* using indicated primers in PATC53 (b) and PANC1 (c, d) cells treated for 24h, 48h, or 72h with DMSO control or 1 µM PRMTi. Data are presented as the mean +/- SEM and *p* values are calculated by two-tailed Student's t-test compared to DMSO-treated cells (n=independent experiments).

# Supplementary Figure 18

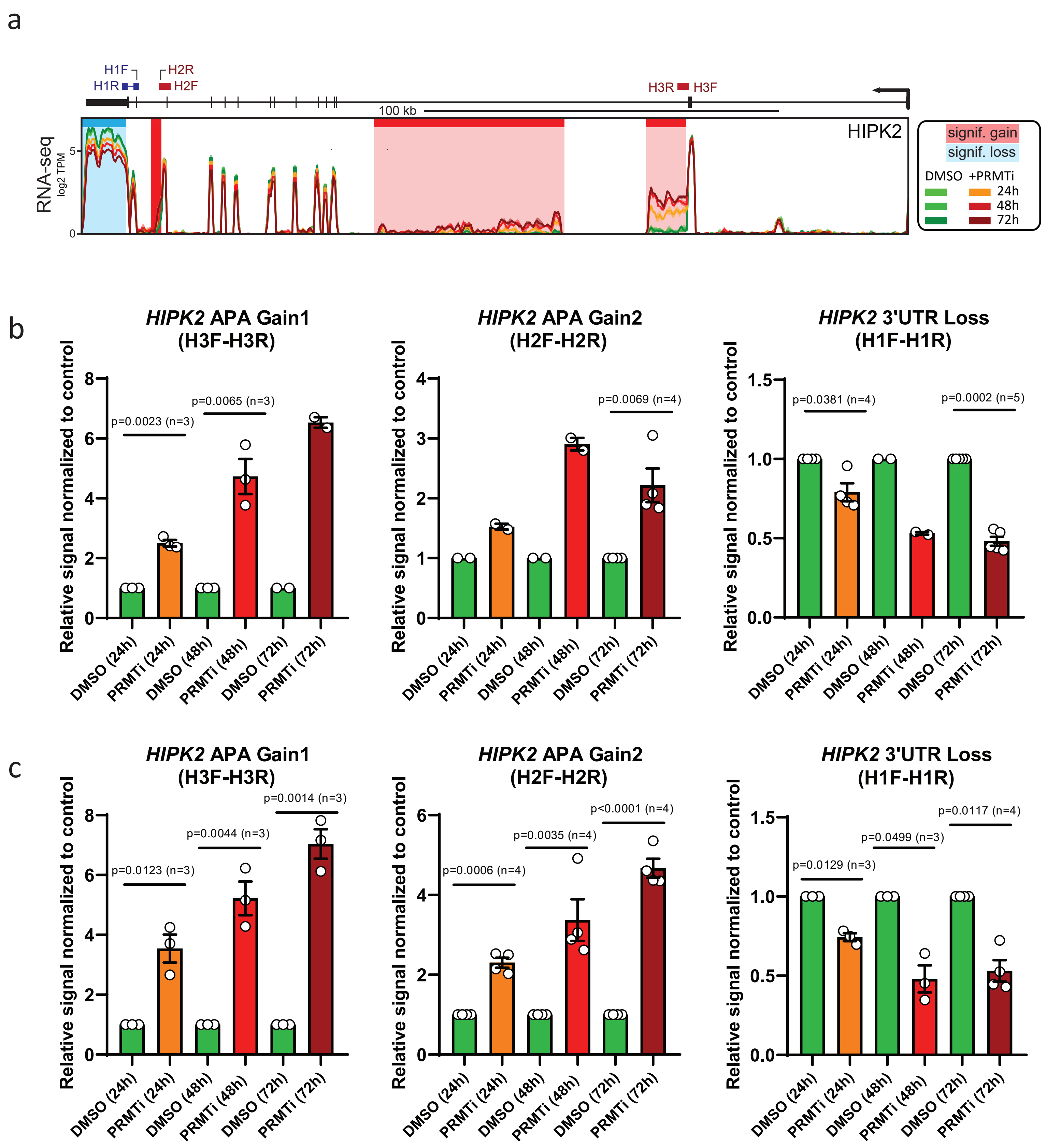

**Supplementary Figure 18. RT-qPCR validation of *HIPK2* alternative polyadenylation.**

(a) Schematic representation of RT-qPCR primers visualized over a composite screenshot of RNA-seq data over *HIPK2* gene in PATC53 cells. Data from treatment with DMSO or 1 µM PRMTi are color-coded as indicated. (b-c) RT-qPCR validation of alternative polyadenylation in *HIPK2* using indicated primers in PATC53 (b) and PANC1 (c) cells treated for 24h, 48h, or 72h with DMSO control or 1 µM PRMTi. Data are presented as the mean +/- SEM and *p* values are calculated by two-tailed Student's t-test compared to DMSO-treated cells (n=independent experiments).

# Supplementary Figure 19

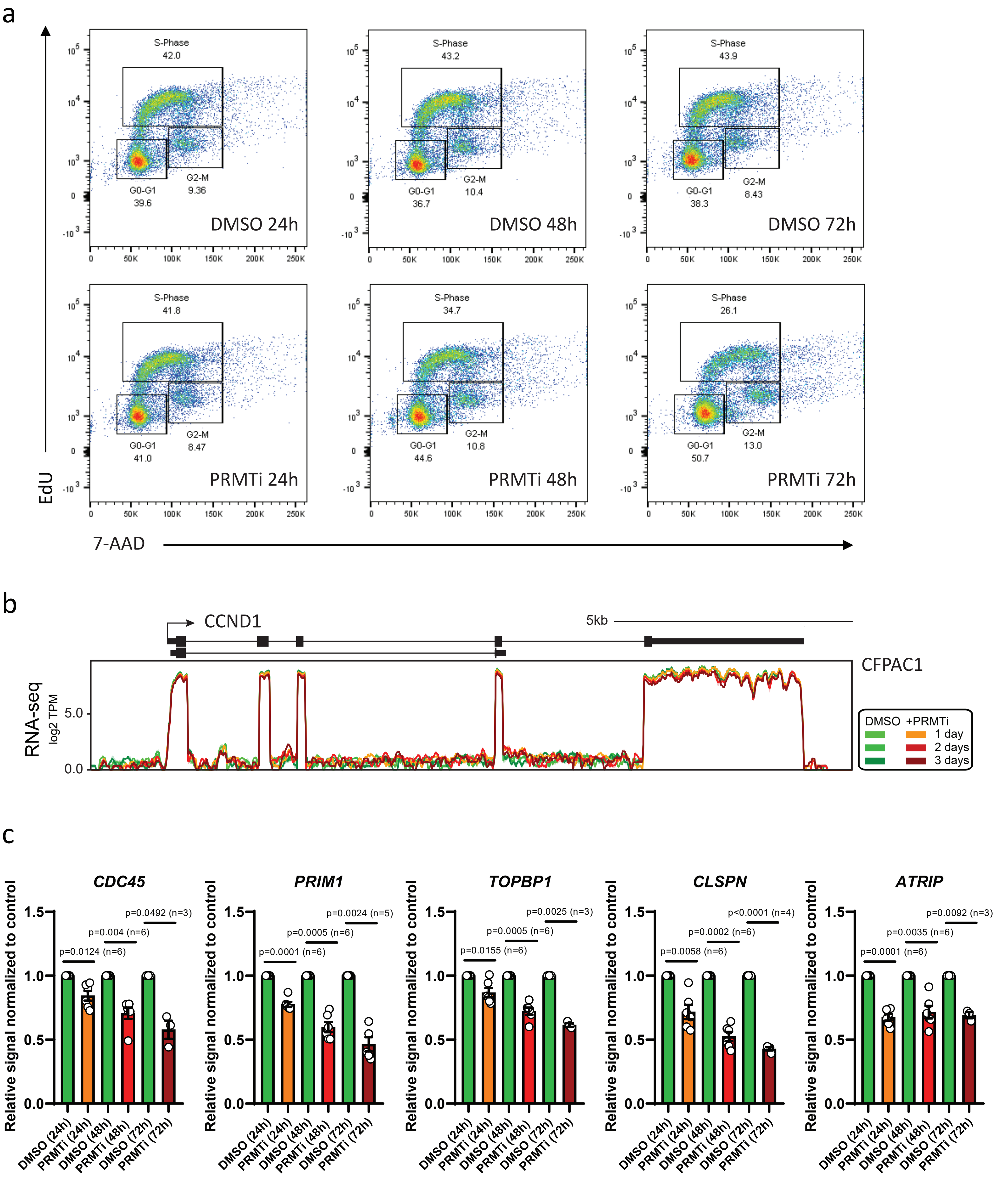

**Supplementary Figure 19. PRMT1 regulates key pathways involved in cell proliferation and DNA replication.**

(a) Cell cycle profile by flow cytometry for EdU incorporation in PATC53 cells treated with 1 µM PRMTi or DMSO control for 24h, 48h, and 72h. 7-AAD was used to estimate DNA content. Gating strategy provided as a Source Data file. (b) Representative composite screenshot of RNA-seq data over *CCND1* in CFPAC1 cells. Data from treatment with DMSO or 1 µM PRMTi are color-coded as indicated. (c) Analysis of gene expression for indicated genes by RT-qPCR in PATC53 cells treated with 1 µM PRMTi or DMSO control for 24h, 48h, and 72h. Data are presented as the mean +/- SEM and *p* values are calculated by two-tailed Student's t-test compared to DMSO treated cells (n=independent experiments).

# Supplementary Figure 20

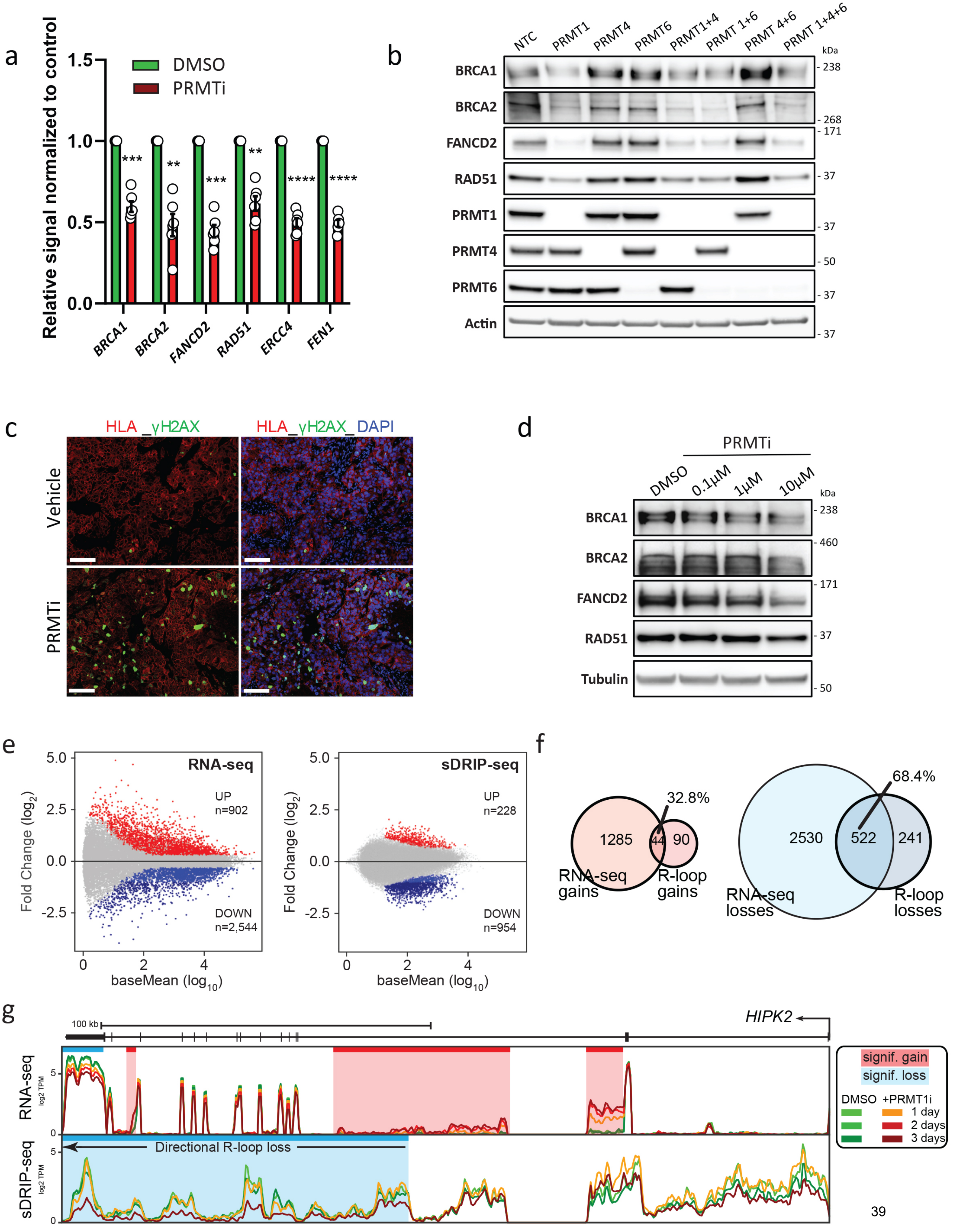

**Supplementary Figure 20. PRMT1 coordinates expression of DNA damage repair genes and maintains genome stability.**

(a) Analysis of gene expression for indicate genes by RT-qPCR in PATC53 cells treated with 1 µM PRMTi or DMSO control for 72h. Data are presented as the mean +/- SEM and *p* values are calculated by two-tailed Student's t-test compared to DMSO controls. For indicated significant differences, exact *p* values are reported from left to right bars: *p*=0.0002 (*BRCA1*); *p*=0.0060 (*BRCA2*); *p*=0.0002 (*FANCD2*); *p*=0.0011 (*RAD51*); *p*<0.0001 (*ERCC4*); *p*<0.0001 (*FEN1*); n=biologically independent samples. (b) Western Blot analysis of indicated proteins in PATC53 cells upon CRISPR/Cas9-mediated knock-down of PRMT1, PRMT4, and PRMT6 individually or in combination. Actin is shown as representative loading control. (c) Immunofluorescence on formalin-fixed paraffin embedded section of PATC53 tumors treated with vehicle or PRMTi at 200 mg/kg QD for 4 days (γH2AX: green; HLA: red; DAPI: blue; scale bar 100µm). (d) Western Blot analysis of indicated proteins in CFPAC1 cells treated with PRMTi at different concentrations. Tubulin is shown as representative loading control. (e) MA plots for RNA-seq (left) and sDRIP-seq (right) highlighting genes with significantly increased or decreased signal in PATC53 cells treated with 1 µM PRMTi for 3 days. (f) Venn diagrams show overlap between genes with significant increases (red) or decreases (blue) in R-loops and expression in PATC53 cells treated with 1 µM PRMTi for 3 days. (g) Representative screenshots of RNA-seq and sDRIP-seq data in PATC53 cells over *HIPK2* gene undergoing directional R-loop losses downstream of APA gains upon 1 µM PRMTi treatment for the indicated timepoints. Top graph represents the RNA-seq data. Bottom graph represents the sDRIP-seq R-loop mapping signal with the line representing the median signal.

# Supplementary Figure 21

A

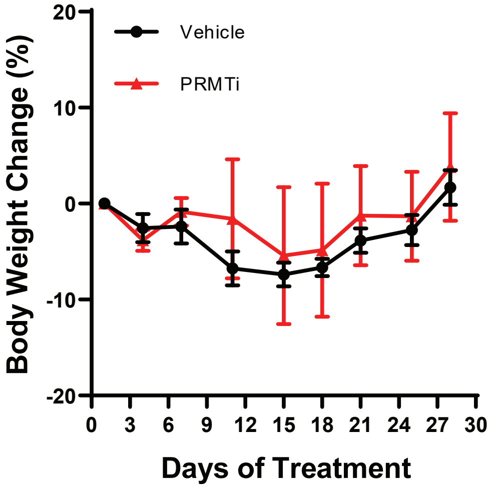


B

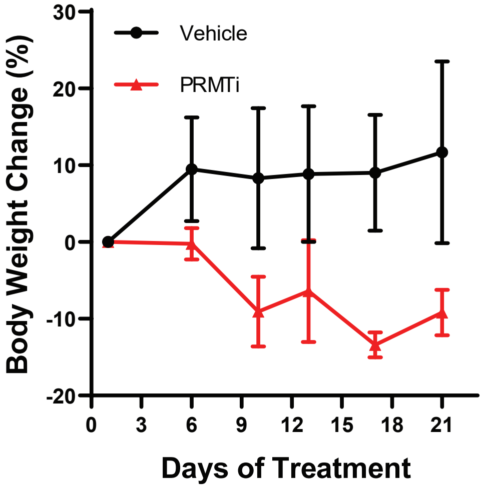

**Supplementary Figure 21. PRMT1 inhibition impairs *in vivo* tumor growth of DNA damage repair deficient PDAC PDXs.**

(a-b) Percentage change of mean body weight (BW) from Day 1 for mice bearing PATX118 (a) or PATX45 (b) xenografts treated with PRMTi at 200 mg/kg QD, 5on/2off for the indicated time. Data are presented as the mean +/- SEM; for (a), n=5 mice/group vehicle; n=3 mice/group PRMTi. For (b), n=3 mice/group.
