# Supplementary material for: PRMT1-dependent regulation of RNA metabolism and DNA damage response sustains pancreatic ductal adenocarcinoma
Source: Nat Commun. 2021 Jul 30;12:4626. doi: 10.1038/s41467-021-24798-y (PMC8324870; doi:10.1038/s41467-021-24798-y)
Supplement: Supplementary file 2 — Description of Additional Supplementary Files [file 41467_2021_24798_MOESM2_ESM.pdf]

Description of Additional Supplementary Files

Supplementary Data 1. Pancreatic cancer cell line mutation profiles.
Genomics variants that are annotated by SNPeffect software to have a high or moderate impact and are observed in cancer gene census genes. For each variant the following attributes are listed: annotated effect (SNPEFF_EFFECT), coding base position (SNPEFF_CDS), amino acid change (SNPEFF_AA), and variant allele percentage (VAF[%]).

Supplementary Data 2. Top3 shRNA and RSA scores for Epigenome screens in PDX models of PDAC.
Scores and ranks for top3 shRNA normalized fold-change and redundant shRNA activity (RSA) are reported for each gene in the Epigenome library in 4 PDX PDAC screens. Top 50 scoring genes are highlighted in light green.

Supplementary Data 3. List of top 30 genes for Epigenome screens in PDX models of PDAC.
Redundant shRNA activity (RSA) and false discovery rate (FDR) are reported for top 30 scoring genes in 4 PDX PDAC screens using the Epigenome library. RSA LogP values less than or equal to -1.5 are highlighted in light red.

Supplementary Data 4. Arginine methylation changes after PRMTi treatment.
PTMscan summary report for Mono-Methyl Arginine (MMA) and Asymmetric Di-Methyl Arginine Antibody (ADMA) changes in PATC53 cells after treatment with 1µM PRMTi for 24h compared to DMSO treatment control. A 2.5-fold cutoff is used as significant for fold changes. Changes >2.5-fold are color coded in the table in green (greater than 2.5-fold) and red (less than -2.5-fold).

Supplementary Data 5. Protein complex mapping for PRMT1 through immunoprecipitation (IP) and mass-spectrometry.
Spectral counts (SpC) and normalized spectral abundance factors (NSAF) are reported for the pull down samples with PRMT1 and Immunoglobulin G (IgG) antibodies, respectively, as is the ratio of the NSAF values for these two conditions (Fold Change).

Supplementary Data 6. Transcriptomic profiling.
First tab (DE Changes) is a table containing a list of genes that undergo significant increase (gain) or decrease (loss) in gene expression (RNA-seq), R-loops (sDRIP-seq), downstream of genes transcription (DoG), and various other gene features changes (Junction and 3'UTR) in PATC53, PANC1, and CFPAC1 cells after treatment with 1µM PRMTi for 1, 2, and 3 days. The total number of genes is depicted at the bottom of the table. Gene expression changes include the whole mRNA region and those showing changes only at the 3'UTR. **Second tab (DE Changes - log2FC)** is a table showing log2 fold change of significantly increased and/or decreased expression of genes and gene features depicted in the first tab. In the event of a gene feature showing multiple increases (or decreases), their values are averaged. **Third tab (PATC53 and PANC1 GO Summary)** and **fourth tab (CFPAC1 GO Summary)** show gene ontology enrichment analyses of significantly increased and decreased genes depicted in the first tab; the third tab shows those that occur in both PATC53 and PANC1 (the enrichment values are averaged between those two) and the fourth tab shows those that occur only in CFPAC1.

Supplementary Data 7. RT-qPCR primers for alternative polyadenylation validation.
