# Supplementary material for: PRMT1-dependent regulation of RNA metabolism and DNA damage response sustains pancreatic ductal adenocarcinoma
Source: Nat Commun. 2021 Jul 30;12:4626. doi: 10.1038/s41467-021-24798-y (PMC8324870; doi:10.1038/s41467-021-24798-y)
Supplement: Supplementary file 10 — Reporting Summary [file 41467_2021_24798_MOESM10_ESM.pdf]

nature research

Corresponding author(s): Virginia Giuliani; Timothy P. Heffernan

Last updated by author(s): Jun 10, 2021

Reporting Summary

Nature Research wishes to improve the reproducibility of the work that we publish. This form provides structure for consistency and transparency in reporting. For further information on Nature Research policies, see our Editorial Policies and the Editorial Policy Checklist.

Statistics

For all statistical analyses, confirm that the following items are present in the figure legend, table legend, main text, or Methods section.

n/a	Confirmed	
☐	☒	The exact sample size (*n*) for each experimental group/condition, given as a discrete number and unit of measurement
☐	☒	A statement on whether measurements were taken from distinct samples or whether the same sample was measured repeatedly
☐	☒	The statistical test(s) used AND whether they are one- or two-sided<br>*Only common tests should be described solely by name; describe more complex techniques in the Methods section.*
☒	☐	A description of all covariates tested
☐	☒	A description of any assumptions or corrections, such as tests of normality and adjustment for multiple comparisons
☐	☒	A full description of the statistical parameters including central tendency (e.g. means) or other basic estimates (e.g. regression coefficient) AND variation (e.g. standard deviation) or associated estimates of uncertainty (e.g. confidence intervals)
☐	☒	For null hypothesis testing, the test statistic (e.g. *F*, *t*, *r*) with confidence intervals, effect sizes, degrees of freedom and *P* value noted<br>*Give P values as exact values whenever suitable.*
☒	☐	For Bayesian analysis, information on the choice of priors and Markov chain Monte Carlo settings
☒	☐	For hierarchical and complex designs, identification of the appropriate level for tests and full reporting of outcomes
☒	☐	Estimates of effect sizes (e.g. Cohen's *d*, Pearson's *r*), indicating how they were calculated

Our web collection on statistics for biologists contains articles on many of the points above.

Software and code

Policy information about availability of computer code

Data collection	RNA sequencing data from NGS sequencers were trimmed using fastq-mcf v.2.4.4 and aligned using bowtie2 v.2.2.6, tophat2 v.2.1.0; Barcode sequencing data from NGS sequencers were processed with bowie2 v2.0.2 and SAMtools v0.1.19; Visiopharm was used for image acquisition; BD FACSDiva 8.0.1 was used to collect flow cytometry data; RT-qPCR data were collected using CFX96 Touch Real-Time PCR Detection System; Western blot images were captured with ImageQuant LAS 4000 (GE Healthcare) or LiCor Odyssey; PHERAstar FS Microplate Reader was used to measure absorbance.
Data analysis	For bioinformatic analysis the following software were used: fastq-mcf v2.4.4, bowtie2 v2.0.2, bowtie2 v2.2.6, tophat2 v2.1.0, SAMtools v0.1.19, bedtools v2.25.0, StochHMM v0.36, HTseq-count v0.6.0, DESeq2 v1.10.1, PANTHER v14.182, wigToBigWig v4, bedToBigBed v2.7 Microsoft Office (version 2105), UCSC Genome Browser; Microsoft Excel or GraphPad Prism 8 were used for plotting and statistical analyses; BioRad CFX Manager 3.1 software was used for RT-qPCR data analysis; MARS Data Analysis Software from BMG LABTECH was used for microplate reader data analysis; FlowJo_v10.7.1 software was used for flow cytometry data analysis.

For manuscripts utilizing custom algorithms or software that are central to the research but not yet described in published literature, software must be made available to editors and reviewers. We strongly encourage code deposition in a community repository (e.g. GitHub). See the Nature Research guidelines for submitting code & software for further information.

Data

Policy information about availability of data

All manuscripts must include a data availability statement. This statement should provide the following information, where applicable:

- Accession codes, unique identifiers, or web links for publicly available datasets
- A list of figures that have associated raw data
- A description of any restrictions on data availability

All data supporting the findings of this study are available within the paper and its supplementary information files. Source data for Figures and Supplementary figures are provided as a Source Data file. All high-throughput sequencing datasets (RNA-seq, DRIP-seq) were deposited on the NCBI GEO website with accession code GSE130242 (https://www.ncbi.nlm.nih.gov/geo/query/acc.cgi?acc=GSE130242). GO Biological Process gene sets were extracted from MSigDB v6.0.

Field-specific reporting

Please select the one below that is the best fit for your research. If you are not sure, read the appropriate sections before making your selection.

[x] Life sciences   [ ] Behavioural & social sciences   [ ] Ecological, evolutionary & environmental sciences

For a reference copy of the document with all sections, see nature.com/documents/nr-reporting-summary-flat.pdf

Life sciences study design

All studies must disclose on these points even when the disclosure is negative.

Sample size	Statistical methods were not used to predetermine sample size. For the analysis of chromosomal aberrations, at least 35 metaphases were analyzed from each condition; for the analysis of fluorescence microscopy images, at least 145 cells were analyzed for each condition providing sufficient power to discriminate between experimental conditions. For in vivo studies, the exact number of mice used in each group is reported in the corresponding figure legend and was sufficient to discern statistically significant differences.
Data exclusions	There was no data exclusion.
Replication	Experiments included independent samples and were independently repeated at least two times or as stated in the text with consistent results.
Randomization	When tumors reached appropriate volume as reported in the Methods section, mice were randomized into indicated treatment groups. For all other experiments, samples were randomly allocated to the experimental groups.
Blinding	For the analysis of chromosomal aberrations and proteomic studies, the researchers were blinded to the identity of the material. For all other experiments, blinding was not possible as experimental set-up and analysis were performed by the same researchers.

Reporting for specific materials, systems and methods

We require information from authors about some types of materials, experimental systems and methods used in many studies. Here, indicate whether each material, system or method listed is relevant to your study. If you are not sure if a list item applies to your research, read the appropriate section before selecting a response.

Materials & experimental systems

n/a	Involved in the study	
[ ]	[x]	Antibodies
[ ]	[x]	Eukaryotic cell lines
[x]	[ ]	Palaeontology and archaeology
[ ]	[x]	Animals and other organisms
[ ]	[x]	Human research participants
[x]	[ ]	Clinical data
[x]	[ ]	Dual use research of concern

Methods

n/a	Involved in the study	
[x]	[ ]	ChIP-seq
[ ]	[x]	Flow cytometry
[x]	[ ]	MRI-based neuroimaging

Antibodies

Antibodies used	PRMT1 (2449; Cell Signaling Technology; 1:1000)<br>PRMT4/CARM1 (A300-421A; Bethyl; 1:2000)<br>PRMT5 (2252; Cell Signaling Technology; 1:1000)<br>PRMT6 (14641; Cell Signaling Technology; 1:1000)<br>Mono-Methyl Arginine (MMA-RGG) (8711; Cell Signaling Technology; 1:1000)

	Asymmetric Di-Methyl Arginine Motif (ADMA) (13522; Cell Signaling Technology; 1:1000)<br>Cas9 (14697; Cell Signaling Technology; 1:1000)<br>Cyclin D1 (2978; Cell Signaling Technology; 1:1000)<br>BRCA1 (14823; Cell Signaling Technology; 1:1000)<br>BRCA2 (10741; Cell Signaling Technology; 1:1000)<br>FANCD2 (16323; Cell Signaling Technology; 1:1000)<br>Rad51 (8875; Cell Signaling Technology; 1:1000)<br>Claspin (NB100-248; Novus Biologicals; 1:1000)<br>ATRIP (ab245632; Abcam; 1:2000)<br>PRIM1 (4725; Cell Signaling Technology; 1:1000)<br>TOPBP1 (14342; Cell Signaling Technology; 1:1000)<br>HSP90 (4874; Cell Signaling Technology; 1:1000)<br>β-Actin (A1978; Sigma; 1:2000)<br>GAPDH (2118; Cell Signaling Technology; 1:1000)<br>Phospho-Histone H2A.X (Ser139) (20E3) Rabbit mAb (Alexa Fluor® 647 Conjugate)(9720; Cell Signaling Technology; 1:100)<br>Anti-phospho-Histone H2A.X (Ser139) Antibody (05-636; Millipore; 1:100)
Validation	All antibodies were purchased from commercial vendors who provide validation information on their website, including published references. In addition, the following antibodies were validated using genetic tools in our laboratory: PRMT1, PRMT4, PRMT6, Mono-Methyl Arginine and Asymmetric Di-Methyl Arginine antibodies.

Eukaryotic cell lines

Policy information about cell lines

Cell line source(s)	PK-59 cell line was obtained from Riken Cell Bank; KP-3 and KP-4 cell lines were obtained from JCRB Cell Bank; Hup-T3 and Hup-T4 were obtained from Sigma; SNU-324 cell line was obtained from Korean Cell Line Bank; Panc1, MiaPaca-2, SW1990, BxPC-3, Capan-1, HPAF-II, Hs766T, Capan-2, Aspc1 and CFPAC1 cell lines were obtained from ATCC; PATC53, PATC124, PATC148 and PATC153 were kindly provided by Dr Jason Fleming and Dr Michael Kim (MDACC); LSL-KrasG12D p53L/+ mouse PDAC cell lines were kindly provided by Dr Haoqiang Ying (MDACC).
Authentication	All cell lines were validated by STR profiling.
Mycoplasma contamination	All cell lines confirmed to be negative for mycoplasma.
Commonly misidentified lines<br>(See ICLAC register)	No commonly misidentified cell lines were used in the study.

Animals and other organisms

Policy information about studies involving animals; ARRIVE guidelines recommended for reporting animal research

Laboratory animals	For xenograft and allograft studies, female CD-1 nude (Charles River) or female NSG (Jackson) between 6-12 weeks old were used as recipients. Mice were housed in ventilated cage enclosures in an environment maintained at 50% humidity (+5%/-10% deviation) with ambient temperature of 72°F (+/-2F° deviation) and 12 hours light/dark cycles.
Wild animals	No wild animals were used in the study.
Field-collected samples	No field-collected samples were used in the study.
Ethics oversight	All animal experiments were approved by the Institutional Animal Care and Use Committee at The University of Texas MD Anderson Cancer Center.

Note that full information on the approval of the study protocol must also be provided in the manuscript.

Human research participants

Policy information about studies involving human research participants

Population characteristics	Patients diagnosed with pancreatic ductal adenocarcinoma (PDAC).
Recruitment	Patients that were deemed surgical cases were consented to LAB07-0854 chaired by J.B.F. (UTMDACC) and agreed to provide tumor tissue for the purpose of model derivation.
Ethics oversight	LAB07-0854 is approved by the UTMDACC Institutional Review Board (IRB) and is reviewed at least annually.

Note that full information on the approval of the study protocol must also be provided in the manuscript.

Flow Cytometry

Plots

Confirm that:

- [x] The axis labels state the marker and fluorochrome used (e.g. CD4-FITC).
- [x] The axis scales are clearly visible. Include numbers along axes only for bottom left plot of group (a 'group' is an analysis of identical markers).
- [x] All plots are contour plots with outliers or pseudocolor plots.
- [x] A numerical value for number of cells or percentage (with statistics) is provided.

Methodology

Sample preparation	At the time of collection, cells were trypsinized with 0.25% trypsin then washed with media and PBS. Cells were then fixed and processed according to the manufacturer's kit instructions.
Instrument	BD LSR Fortessa Analyzer or LSR Fortessa X-20 Analyzer.
Software	BD FACSDiva 8.0.1 software was used to collect data and FlowJo_v10.7.1 software was used for data analysis.
Cell population abundance	For each sample, 10000 events were collected and over 80% of events were identified as singlets.
Gating strategy	Cell were first gated on forward scatter (FSC) and side scatter (SSC) to identify the cell population of interest and exclude debris. Singlets were then gated by FSC-H vs FSC-A and doublets excluded. Two parameter density plots were then used to identify BrdU or EdU positive cells.

- [x] Tick this box to confirm that a figure exemplifying the gating strategy is provided in the Supplementary Information.
